# Efficient constitution of a library of rotenoid analogs active against *Trypanosoma cruzi* from a digitalized plant extract collection†

**DOI:** 10.1039/d4ra08652j

**Published:** 2025-05-09

**Authors:** Arnaud Gaudry, Laurence Marcourt, Marcel Kaiser, Julien Flückiger, Bruno David, Antonio Grondin, Jean-Robert Ioset, Pascal Mäser, Emerson Ferreira Queiroz, Pierre-Marie Allard, Jean-Luc Wolfender

**Affiliations:** a Institute of Pharmaceutical Sciences of Western Switzerland, University of Geneva 1211 Geneva 4 Switzerland arnaud.gaudry@unige.ch jean-luc.wolfender@unige.ch laurence.marcourt@unige.ch fluckiger.julien@gmail.com emerson.ferrerira@unige.ch; b School of Pharmaceutical Sciences, University of Geneva 1211 Geneva 4 Switzerland; c Department of Medical and Parasitology and Infection Biology, Swiss Tropical and Public Health Institute 4123 Allschwil Switzerland marcel.kaiser@swisstph.ch pascal.maeser@swisstph.ch; d Faculty of Science, University of Basel 4002 Basel Switzerland; e Green Mission Pierre Fabre, Institut de Recherche Pierre Fabre 3 Avenue Hubert Curien 31562 Toulouse France brunoxdavid@gmail.com antonio.grondin@pierre-fabre.com; f Drugs for Neglected Diseases Initiative (DNDi) 1202 Geneva Switzerland jrioset@dndi.org; g Department of Biology, University of Fribourg 1700 Fribourg Switzerland pierre-marie.allard@unifr.ch

## Abstract

Natural products (NP) have proven to be a rich source of potentially bioactive compounds, and metabolomics is the current method of choice for characterizing natural extracts. To integrate the vast amount of data and information produced by modern metabolomics workflows, we recently developed a sample-centric approach for the semantic enrichment and alignment of metabolomics datasets. The resulting Experimental Natural Products Knowledge Graph (ENPKG) is queryable and integrates both newly acquired digitalized experimental data and information, and previously reported knowledge. It allows the highlighting of putative bioactive compounds at the extract level by comparing, for example, the occurrence of compounds of a given chemical class with bioactivity results. Using this approach, we recently described potent anti-*Trypanosoma cruzi* activity of two rotenoids, deguelin and rotenone. These compounds were identified in six active extracts from four plant species: *Cnestis palala* (Connaraceae), *Chadsia grevei*, *Pachyrhizus erosus*, and *Desmodium heterophylum* (Fabaceae). In this work, we present the results of the phytochemical investigation of four of these extracts and the establishment of a library of structural analogs for *in vitro* bioactivity testing. This work led to the isolation, characterization, and biological evaluation of the anti-*T. cruzi* potential of 41 compounds, including 11 rotenoids and seven compounds reported for the first time. Thanks to modern metabolite annotation and single-step isolation procedures, this work also demonstrates the possibility of considering natural extract libraries as a reservoir of rapidly accessible pure NPs. This perspective could increase the options for NP research and help accelerate NP drug discovery efforts.

## Abstract 2

Branching out into knowledge! We build a KG to enrich and align metabolomics datasets, revealing bioactive compounds from plant extracts. We uncover anti-*T. cruzi* activity of rotenoids from 4 plant species, and isolate and characterize 41 analogs.

## Graphical abstract

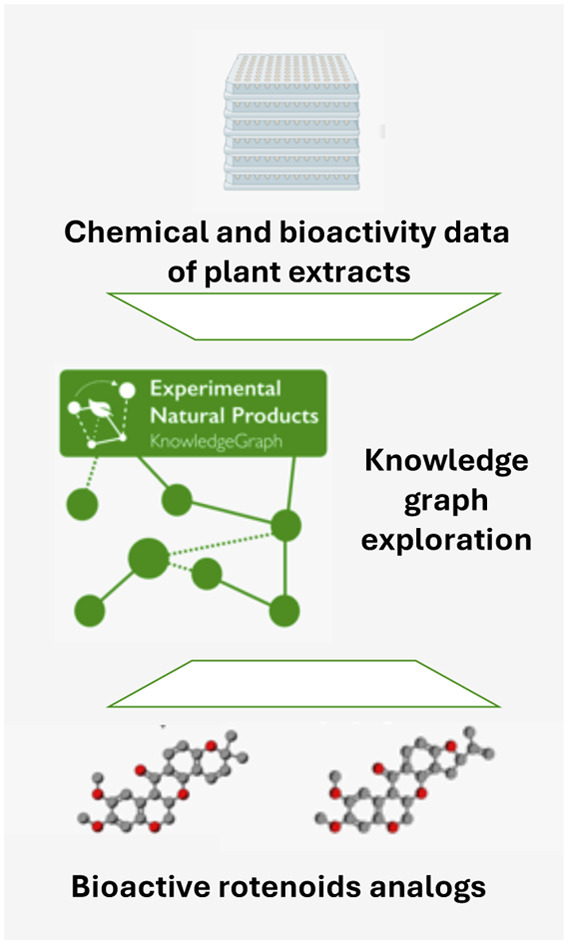

## Introduction

Chagas disease, or American trypanosomiasis, is a potentially fatal infection caused by *Trypanosoma cruzi*.^1^ Six to seven million people are estimated to be chronically infected with *T. cruzi*, mostly in Latin America, and are at risk of developing severe complications such as arrhythmia, dilated heart, or dilated colon, making Chagas disease an important public health issue.^2–4^ Current treatments – benznidazole and nifurtimox – are sometimes associated with severe adverse effects and suboptimal efficacy.^5^ There is therefore a need for new drugs targeting asymptomatic infections before complications appear.^6^

Plants are an important resource for discovering new antiparasitic molecules, with the well-known example of artemisinin isolated from *Artemisia annua*.^7^ Because of their particular properties as distinct from synthetic compounds (generally with more sp^3^ carbons, chiral centers, or oxygen atoms), NPs are more pertinent to biological targets and have higher hit rates in drug discovery screening programs compared to compounds of synthetic origin.^8–13^ However, obtaining new drug candidates from natural sources also comes with challenges: their structural complexity makes NPs difficult to synthesize, and the quantity and diversity of molecules in natural extracts (NE) make them challenging to comprehensively characterize.

Modern metabolomics methods have been effectively used to decipher NE content.^14^ These methods are usually based on ultra-high-performance liquid chromatography coupled with data-dependant acquisition (DDA) tandem high-resolution mass spectrometry (UHPLC-HRMS^2^) analysis, typically followed by molecular networking (MN) and spectral annotation.^15,16^ Different spectral annotation tools exist for labelling unknown spectra at the structural level, such as SIRIUS/CSI:FingerID or spectral matching, or CANOPUS at the chemical class level, for example.^16–19^ On the other hand, the same spectral and structural datasets can also be leveraged within research projects for the discovery of bioactive compounds. Several computational approaches have been developed, such as multi-informative MN, bioactivity-based MN, or Compound Activity Mapping, which allow the integration of bioassay screening results and metabolomics data to highlight potentially active compounds in NE prior to any physical isolation processes.^20–22^

These computational developments led to increasing data volumes in NP research and triggered the need for advanced data analytics methods. We, therefore, recently developed a sample-centric framework to semantically enrich and align LC-MS data from many samples into a single knowledge graph, the Experimental Natural Products Knowledge Graph (ENPKG). We used this framework to explore the results from antiparasitic screening and metabolomic profiling of 1600 plant extracts, and anticipated two putative structural scaffolds that could be responsible for the anti-*T. cruzi* bioactivities observed, namely rotenoids and quinolones.^23–27^ Among the eight extracts active against *T. cruzi*, the activity of one of them (*Melochia umbellata* (Houtt.) Stapf stems) could be explained by the presence of quinolone derivatives (query). Due to the extensive work already done on the anti-*T. cruzi* activity of this scaffold, it was not deemed necessary to carry out phytochemical work on this extract.^25,26,28,29^ For six other active extracts, the activity was linked to rotenoid derivatives, particularly deguelin and rotenone. Biological evaluation of the commercial standards of both compounds showed nanomolar IC_50_ against the amastigote form of the parasite without toxicity to the host cell.^27^ Rotenoid derivatives demonstrated significant potential as antiparasitic agents in other studies, particularly against nematodes and protozoan parasites. Deguelin and rotenone, exhibit potent nematocidal activity against *Haemonchus contortus*, with deguelin showing selective inhibition of larval motility without high cytotoxicity.^30^ In another study, modified rotenone derivatives have also displayed antiplasmodial activity against *Plasmodium falciparum* (EC_50_ values <50 μM) and moderate effects against *Leishmania panamensis*, though cytotoxicity remains a challenge for some compounds.^31^ Since, to the best of our knowledge, the anti-*T. cruzi* activity of rotenoids had not been reported, we went on to investigate the phytochemistry of the six rotenoid-containing active extracts. We used an optimized single-step chromatography isolation workflow to efficiently and rapidly generate a library of rotenoid and related isoflavone analogs and to test them *in vitro*. This approach enabled an efficient translation from metabolite profiling results to targeted isolation of selected compounds and assessment of their *in vitro* bioactivity.

## Results and discussion

**Fig. 1:**
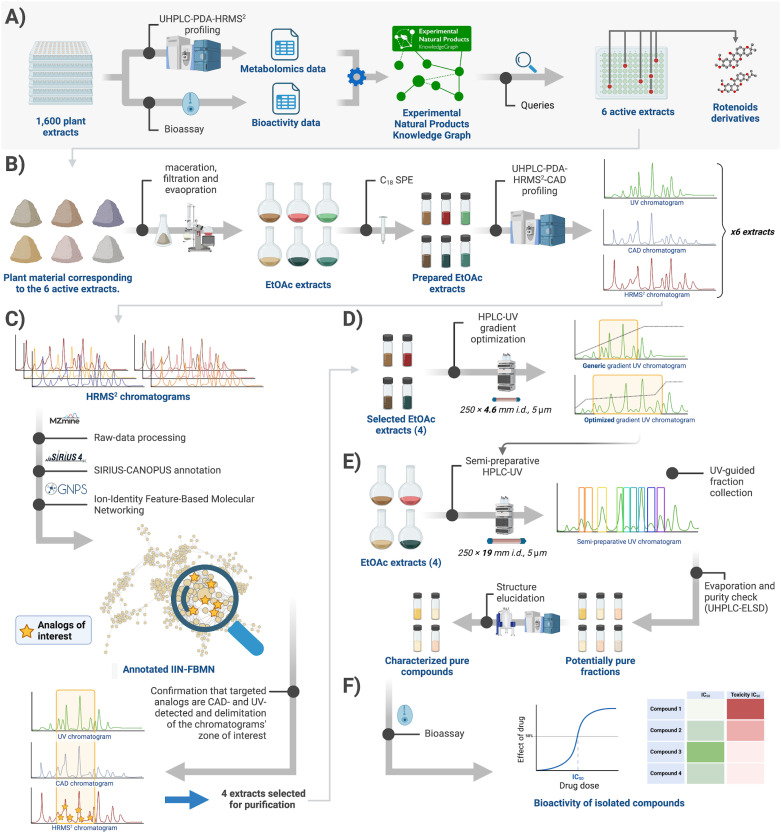
Experimental workflow of the present study. (A) Identification of rotenoids as responsible for the activity of six extracts against *T. cruzi* using the Experimental Natural Products Knowledge Graph framework.^27^ (B) Plant material corresponding to the six active extracts containing rotenoids [*Desmodium heterophyllum* underground parts, *Chadsia grevei* roots, and bark, *Pachyrhizus erosus* leaves, and *Cnestis palala* woody stems and roots (see ref. 27 for details)] was reextracted by maceration. The obtained EtOAc extracts were prepared using SPE and profiled on a UHPLC-PDA-HRMS^2^-CAD platform. (C) The LC-HRMS^2^ data were processed using MZmine 3, SIRIUS-CANOPUS, and GNPS to generate an annotated Ion-Identity Feature-Based Molecular Network (IIN-FBMN). LC-MS peaks corresponding to rotenoids were retrieved using the CANOPUS chemical class annotations. Using the PDA and CAD data, we could confirm they were UV-reactive and present in a relative amount sufficient for isolation. (D) The HPLC gradient was optimized for better separating the targeted compounds for the four selected extracts, one by species. (E) Finally, targeted compounds were obtained using semi-preparative HPLC in a single chromatography step. Pure compounds' structural elucidation was performed using NMR, HRMS, and chiroptical methods, and finally (F) their bioactivity against *T. cruzi* was assessed *in vitro*.

By integrating metabolite profiling and bioactivity data into the ENPKG (https://enpkg.commons-lab.org/graphdb/), we were able to discover that six of the eight extracts active against *T. cruzi* in the initial screening contained many potential rotenoid derivatives. This was done by analyzing the CANOPUS annotations of the different active extracts at the NPClassifier class level (query: https://enpkg.commons-lab.org/graphdb/sparql?savedQueryName=count_features_canopus_rotenoids_in_samples&owner=admin) and the individual structural annotations (query: https://enpkg.commons-lab.org/graphdb/sparql?savedQueryName=count_annotation_occurence_in_selection_vs_dataset&owner=admin).^19,27,32^ These extracts were obtained from the following species: *Desmodium heterophyllum* (Willd.) DC., *Chadsia grevei* Drake, *Pachyrhizus erosus* (L.) Urb. (Leguminosae), and *Cnestis palala* (Lour.) Merr. (Connaraceae).^27^ For two species, *C. grevei* and *C. palala*, two extracts from two different plant parts were active (Fig. 1A). The ENPKG allowed us to highlight the chemical similarity between these six extracts that are not closely related taxonomically: three Fabaceae from different genera and one Connaraceae.^27^

As the extracts used in the initial screening were not available in sufficient quantity for further fractionation, dried plant material from the six active plant parts was extracted at a larger scale using maceration with solvents of increasing polarities (hexane, ethyl acetate, and methanol). Ethyl acetate extracts were used for the remainder of the work, as this was the solvent used to generate the initial 1600-extract library.^23^ To confirm the presence of rotenone and deguelin analogs in the newly obtained extracts, we used UHPLC-DDA-HRMS^2^-based profiling for metabolite annotation. The analysis of the new extracts was performed using a better chromatographic resolution than was used when obtaining the initial screening metabolomics data (100 mm *vs.* 50 mm column, longer elution gradient), and charged aerosol detector (CAD) detection was added to obtain semi-quantitative information about the composition of the extracts (Fig. 1B).^33,34^ The data obtained from this improved metabolite profiling were subjected to Ion-Identity (IIN) Feature-Based Molecular Networking (FBMN) combined with CANOPUS chemical class annotation to highlight compounds of interest^9–13^ (Fig. 1C). Four extracts covering a maximum of the chemical diversity from the six active extracts, one per species, were selected to further isolate the targeted compounds. These were isolated using one-step high-resolution semi-preparative HPLC and characterized using HRMS, NMR, and chiroptical methods (Fig. 1D and E).^35^ Finally, the cytotoxicity of the compounds and their activity against *T. cruzi* were evaluated *in vitro* (Fig. 1F).

### Ion-identity molecular networking and SIRIUS-CANOPUS annotation of the selected extracts

The extracts UHPLC-HRMS^2^ profiling data in positive ionization (PI) mode were processed using the Ion Identity-FBMN workflow^36^ and SIRIUS/CSI:FingerID and CANOPUS^17–19^ were used to annotate the content of the extracts and confirm the presence of rotenoids. The IIN-FBMN allows both (1) the grouping and annotating of features corresponding to the same molecular species (adducts, in-source fragments, *etc.*, for each chromatographic peak) using the MS^1^ feature peak-shape correlation and the difference in *m*/*z* and (2) visualization of spectral similarities among features.^36^ CANOPUS, on the other hand, is a computational tool that allows the annotation of unknown MS^2^ spectra at the pathway, superclass, and class level following the NPClassifier taxonomy.^32^ Because a chemical class can be assigned even if the structure is not reported in any database, it is a particularly relevant tool for annotating analogs of a given chemical class, such as, in this case, rotenoids.

**Fig. 2:**
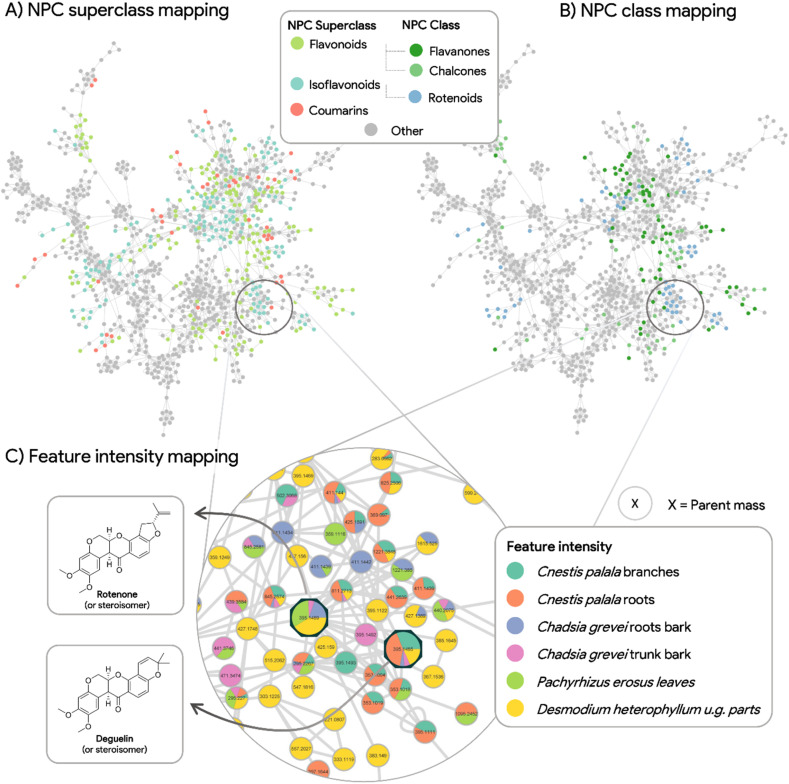
Principal cluster (representing 1014 out of 1990 nodes) of the collapsed IIN-FBMN analysis performed on the six active extracts. Using CANOPUS annotations at the superclass level (A) indicates that the three main chemical superclasses present are isoflavonoids, flavonoids, and coumarins. At the chemical class level (B), the main classes annotated by CANOPUS are chalcones, flavanones, and rotenoids. Together with identifying either deguelin or rotenone (C) in all six extracts, these data confirm that these extracts are interesting sources of potential isoflavones/rotenoids analogs.

In the annotated IIN-FBMN from the six newly obtained extracts, after feature alignment a total of 2639 features were deconvoluted among the extracts, and this number was reduced to 1990 collapsed features after IIN grouping, with 680 of them corresponding to an IIN unique molecular species (*i.e.*, at least two correlated adducts/in-source fragments detected). The IIN-MN clustering (cosine and adducts links) returned a main cluster of 1014 nodes containing most of the compounds of interest (see Fig. 2 for this main cluster and ESI Fig. 7† for the whole MN). Among the 1990 features of the whole MN, at the NPClassifier superclass level, 391 were annotated as flavonoids, 297 as isoflavonoids, and 134 as coumarins by CANOPUS (Fig. 2A and ESI Fig. 7A†).^19,32^ At the more scaffold-specific defined NPClassifier class level, the main classes are flavanones (141 features), chalcones (123), and rotenoids (116) (Fig. 2B and ESI Fig. 7B†), with coumarins not appearing in the top 3. This analysis at the chemical class level confirms that the selected extracts are rich in rotenoids and other (iso)flavone derivatives, as expected from the ENPKG results. Using standards, we could also unambiguously identify rotenone and deguelin in the MN (Fig. 2C). By visualizing the relative feature intensity mapping on the FBMN nodes (Fig. 2C), we observed that deguelin was detected in all extracts except *P. erosus* leaf extract, and rotenone in all extracts except the two *C. palala* extracts. Other potential derivatives were highlighted by their close relationship in the MN, and the relative feature intensity mapping shows that they were either specific to a given species or shared among different extracts (Fig. 2C). These data confirm that the newly obtained extracts are rich in isoflavone and rotenoid derivatives and, therefore, were used to generate a library of isolated rotenoid analogs.

### Targeted isolation of rotenoids and isoflavonoids derivatives

**Fig. 3:**
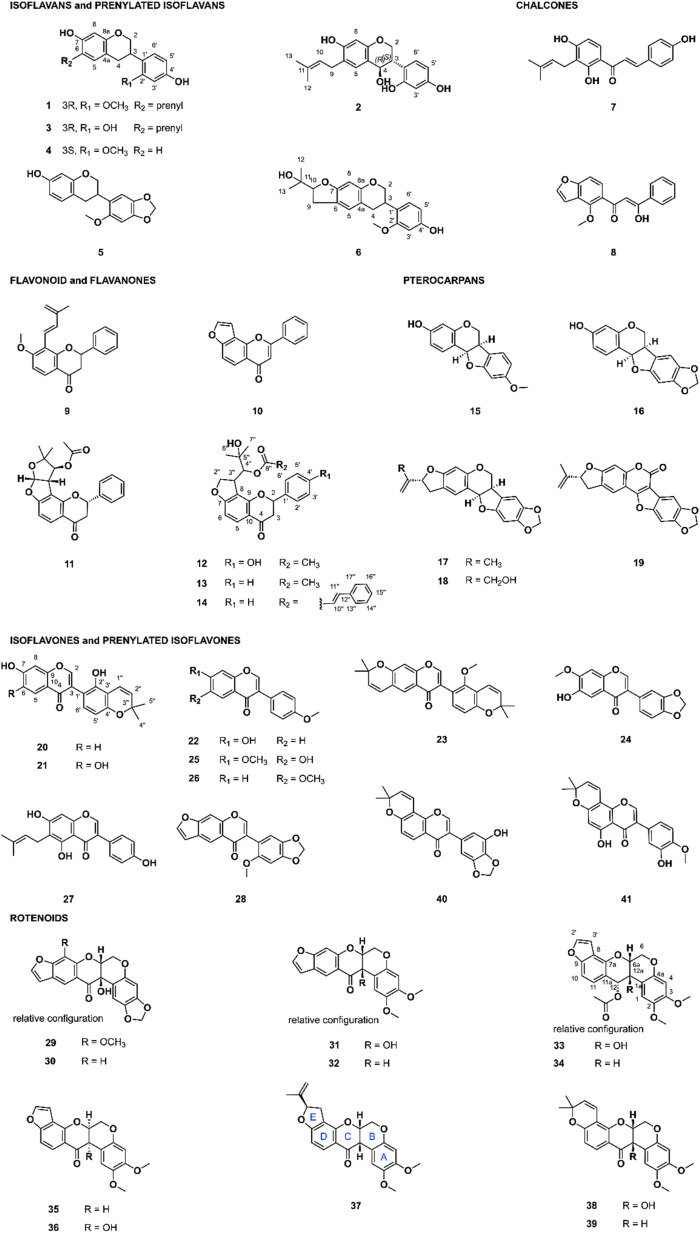
Isolated compounds from *Cnestis palala*, *Chadsia grevei*, *Pachyrhizus erosus*, and *Desmodium heterophyllum* (6 isoflavans and prenylated isoflavans, 2 chalcones, 6 flavonoid/flavanones, 5 pterocarpans, 11 isoflavones/prenylated isoflavones, and 11 rotenoids). For rotenone (37), the ring nomenclature has been added to ease the structure–activity discussion.

The FBMN indicated that the retention time (RT) of the above-mentioned features annotated as rotenoid and isoflavonoid derivatives was mainly between 4.5 and 9.5 min. In addition, the semi-quantitative CAD signal confirmed that targeted compounds were apparently present in sufficient quantity to isolate amounts suitable for characterization and bioactivity evaluation starting from about 120 mg of extracts (ESI Fig. 1–6†). Because both *Chadsia grevei* and *Cnestis palala* extracts presented similar chromatographic profiles, only one extract from each species (*C. grevei* roots bark and *C. palala* roots, respectively) was selected for isolation. We performed gradient optimization to maximize the resolution in the chromatographic area of interest using analytical HPLC coupled to a photodiode array (PDA) detector (4.6 × 250 mm I.D., 5 μm, C_18_ column). The obtained conditions were transferred to semi-preparative HPLC (19 × 250 mm I.D., 5 μm, C_18_ column) coupled to dry-load injection (see ESI Fig. 8 and 9†) using a geometrical gradient transfer.^35^ Since all targeted compounds share a characteristic UV-PDA chromophore, their isolation could be easily monitored using UV detection alone. Following this process, compounds of interest were isolated in a single high-resolution semi-preparative chromatography step. This led to the isolation of 9 compounds from *Cnestis palala*, 11 from *Chadsia grevei*, 17 from *Pachyrhizus erosus*, and 9 from *Desmodium heterophyllum*. This represents a total of 41 different pure NPs (Fig. 3), some being isolated in multiple extracts (see ESI Table 5† for the details about the originating extract(s) for each compound).

Of the 41 isolated compounds, 34 had already been reported and identified through their NMR and chiroptical data, while seven (1, 6, 12, 13, 14, 21, and 33) were not reported to our knowledge. Regarding scaffolds, 6 were isoflavans or prenylated isoflavans, 2 were chalcones, 6 were flavonoids or flavanones, 5 were pterocarpans, 11 were isoflavones or prenylated isoflavones, and 11 were rotenoids. The chemical classes of the isolated compounds were consistent with that expected from the CANOPUS annotations.

The 34 known compounds were identified based on their HRMS and NMR data as bavaisoflavanol (2), manuifolin H (3), 7,4′-dihydroxy-2′-methoxyisoflavan (4), astraciceran (5), isobavachalcone (7), pongamol (8), dehydroisoderricin (9), lanceolatin B (10), (−)-purpurin (11), medicarpin (15), maackiain (16), emoroidocarpan (17), (2′*R*)-4′-hydroxyemoroidocarpan (18), tephcalostan (19), glabrone (20), formononetin (22), munetone (23), acicerone (24), alfalone (25), afrormosin (26), wighteone (27), dehydroneotenone (28), 12*a*-hydroxypachyrhizone (29), 12*a*-hydroxydolineone (30), 12*a*-hydroxyerosone (31), erosone (32), 12-deoxo-12α-acetoxyelliptone (34), elliptone (35), 12*a*-hydroxyelliptone (36), rotenone (37), tephrosin (38), deguelin (39), norisojamaicin (40) and 3′-hydroxy-4′-*O*-metyhlerrone (41). The original references used for identification are available in ESI.†

The seven unreported compounds were identified as 2′-*O*-methylmanuifolin H (1), 4-(2-(2-hydroxypropan-2-yl)-2,3,6,7-tetrahydro-5*H*-furo[3,2-*g*]chromen-6-yl)-3-methoxyphenol (6), 2-hydroxy-1-(2-(4-hydroxyphenyl)-4-oxo-3,4,8,9-tetrahydro-2*H*-furo[2,3-*h*]chromen-9-yl)-2-methylpropyl acetate (12), 2-hydroxy-2-methyl-1-(4-oxo-2-phenyl-3,4,8,9-tetrahydro-2*H*-furo[2,3-*h*]chromen-9-yl)propyl acetate (13), 2-hydroxy-2-methyl-1-(4-oxo-2-phenyl-3,4,8,9-tetrahydro-2*H*-furo[2,3-*h*]chromen-9-yl)propyl cinnamate (14), 5′,6,7-trihydroxy-2′,2′-dimethyl-2′*H*,4*H*-[3,6′-bichromen]-4-one (21), and 12-deoxo-12α-acetoxy-12*a*-β-hydroxyelliptone (33). Details about the structural elucidation are shown in ESI.†

Among these isolated compounds, deguelin (39)^37^ had already been reported in *C. grevei* and rotenone (37),^38^ erosone (32),^39^ 12*a*-hydroxypachyrrhizone (29),^38,40^ 12*a*-hydroxydolineone (30)^40,41^ and dehydroneotenone (28)^41^ in *P. erosus*. To our knowledge, none of these isolated compounds had been reported for *D. heterophyllum* and *C. palala*. While the occurrence of these flavone derivatives in Leguminosae species was expected,^42^ the Connaraceae family (*C. palala*) has not been subject to extensive phytochemical investigation and this is the first reported occurrence of rotenoids in this family.

### Bioactivity of isolated compounds

The bioactivity of isolated compounds against intracellular *T. cruzi* amastigotes grown in rat L6 cardiomyocytes is presented in Table 1. Only tephrosin (38) showed activity in the nanomolar range (IC_50_ of 0.04 μM) comparable to that of deguelin (39, 0.02 μM) and rotenone (37, 0.01 μM). Interestingly, the eight other rotenoids tested (compounds 29–36) were not active against *T. cruzi*, demonstrating that the activity is very specific even for compounds of the same chemical class. Rotenone and deguelin are known to be strong mitochondrial complex I (also known as NADH:ubiquinone oxidoreductase) inhibitors,^43–45^ while different activities between closely related rotenoids (rotenone, 5′-α-epirotenone, and 5′-β-epirotenone) has already been shown. The activity of rotenone on mitochondrial complex I was found to be dependent on the bent form of the rotenoid at the B/C ring junction, E ring substitution, ligand flexibility, and 2,3 dimethoxy substitution.^46–49^ Evaluation of the activity of a series of rotenone and deguelin analogs on the NADH:ubiquinone oxidoreductase also demonstrated important differences in activity.^50^ It is evident from the various examples presented that the activities of rotenoids on mitochondrial complex I can vary significantly, even with minor structural changes. The variation in activity observed on our rapidly generated series of structural analogs points in the same direction. As shown in Fig. 3 and Table 1, the cyclic and bent form of the rotenoid at the B/C ring junction is essential for activity, since compounds 40 and 41 were not active. The lack of activity of compounds 33–36 highlights the central role of an aliphatic substitution on the E ring for bioactivity.

**Table 1:** Bioactivity of isolated compounds against *T. cruzi* amastigotes

Compound	IC_50_^a^ (μM)		Selectivity index^c^
	*T. cruzi* amastigotes	Cytotoxicity^b^	
1	28.19	99.00	3.5
2	43.08	49.51	1.1
3	28.31	106.01	3.7
4	247.34	358.06	1.4
5	94.07	75.42	0.8
6	202.58	153.62	0.8
7	60.12	103.59	1.7
8	26.83	73.56	2.7
9	66.17	95.36	1.4
10	9.58	130.21	13.6
11	1.78	6.25	3.5
12	58.80	99.41	1.7
13	122.72	130.54	1.1
14	15.97	81.62	5.1
15	201.83	226.25	1.1
16	118.91	155.84	1.3
17	47.81	84.77	1.8
18	40.12	118.19	2.9
19	65.59	118.70	1.8
20	149.40	186.27	1.2
21	56.76	76.63	1.4
22	216.58	224.04	1.0
23	7.35	38.82	5.3
24	86.78	186.38	2.1
25	240.87	263.17	1.1
26	205.84	255.46	1.2
27	46.55	103.59	2.2
28	3.59	18.27	5.1
29	93.25	124.63	1.3
30	195.01	141.08	0.7
31	19.74	113.35	5.7
32	39.31	114.52	2.9
33	65.11	98.21	1.5
34	27.12	103.94	3.8
35	18.25	99.05	5.4
36	5.42	100.18	18.5
37	0.01	0.48	51.7
38	0.04	72.37	1937.0
39	0.02	19.40	1148.5
40	21.83	123.37	5.7
41	8.04	55.68	6.9
Benznidazole	3.14		
Podophyllotoxin		0.02	

a The IC_50_ are the means of two independent assays.

b Rat skeletal myoblast (L6 cells).

c Selectivity index (SI) = IC_50_ cytotoxicity/IC_50_ against *T. cruzi*.

In *T. cruzi* epimastigotes, rotenone has been observed to have a weak effect on NADH-fumarate reductase, NADH-cytochrome *c* reductase, succinate–cytochrome *c* reductase, and *sn*-glycerol-phosphate cytochrome *c* reductase.^51^ In addition, *T. cruzi* kDNA mutants with deletions in complex I subunits demonstrated no changes in mitochondrial bioenergetics, production of ROS, or redox state when compared to their wild-type counterparts, implying a restricted role for complex I in the functioning of *T. cruzi* epimastigotes.^52^ However, no studies have been performed on the amastigote form of the parasite to our knowledge; therefore, the role of complex I in the metabolism of this form remains unclear.^53^ The very selective anti-*T. cruzi* activity observed for compounds 37–39, known complex I inhibitors, compared to that of the large set of analogs tested, suggests that the observed activity may be linked to complex I inhibition. However, because of the phenotypic nature of the assay used, we can not exclude that the activity observed is due to action on one or more different target(s). Further target deconvolution experiments are needed to confirm the target identity.

## Material and methods

### General experimental procedures

UV spectra were recorded on a JASCO J-815 spectrometer (Loveland, CO, United States) in MeOH, using a 1 cm cell. The optical rotations were measured in acetonitrile on a JASCO P-1030 polarimeter (Loveland, CO, United States) in a 1 ml, 10 cm tube. NMR data were recorded on a Bruker Avance Neo 600 MHz NMR spectrometer equipped with a QCI 5 mm cryoprobe and a SampleJet automated sample changer (Bruker BioSpin, Rheinstetten, Germany). 1D and 2D NMR experiments (^1^H, COSY, ROESY, HSQC, and HMBC) were recorded in CD_3_OD or CDCl_3_. The residual CD_3_OD or CDCl_3_ signals (*δ*_H_ 3.31; *δ*_C_ 49.0 and *δ*_H_ 7.26; *δ*_C_ 77.2, respectively) were used as internal standards for ^1^H and ^13^C NMR, respectively. Chemical shifts are reported in parts per million (*δ*) and coupling constants (*J*) in Hz.

### Plant material

The plants investigated are part of the Pierre Fabre Laboratories (PFL) collection, which is among one of the largest collections of plant samples worldwide with over 17 000 unique samples, and was registered on April 1, 2020, at the European Commission under the accession number 03-FR-2020.^23,54^ This registration certifies that the collection meets the criteria set out in the EU ABS Regulation which implements at EU level the requirements of the Nagoya Protocol regarding access to genetic resources and the fair and equitable sharing of benefits arising from their utilization. The PFL supplied all the vegetal material (grounded dry material). Precise localization of the initial collection, unique ID (VXXXXX) and barcode are stored in databases. The plant material was dried, and then grounded and stored in plastic pots at a controlled temperature and humidity in the PFL facilities.

### Plant material extraction

Plant material was extracted with three solvents of increasing polarity (hexane, ethyl acetate, and methanol). For each solvent, the dried plant material was macerated 3 consecutive times for 12 hours with a 1 : 10 (m/v) solvent ratio. The macerate was filtered, and the filtrate was dried using a rotavapor. For the rest of the work, EtOAc extracts were used. The different PFL identifiers of the plant parts used in this work are: *Desmodium heterophyllum* (Willd.) DC. underground parts: V114311, *Chadsia grevei* Drake roots: V113889, *C. grevei* bark: V113890, *Pachyrhizus erosus* (L.) Urb. leaves: V112311, and *Cnestis palala* (Lour.) Merr. woody stems: V113330, *C. palala* roots: V113332.

### UHPLC-PDA-HRMS^2^-CAD analysis

Ethyl acetate extracts were analyzed using UHPLC-HRMS/MS-CAD-PDA. Prior to analysis, too apolar compounds of the extracts were removed by C_18_ solid phase extraction (SPE 1000 mg/12 ml, Finisterre, Teknokroma, Barcelona, Spain). About 10 mg of extract was loaded on the cartridge, followed by elution with 10 ml of methanol (MeOH). The prepared extracts were dried under nitrogen flux. For UHPLC-PDA/HRMS^2^/CAD analysis, extracts were diluted in MeOH at a 5 mg mL^−1^ concentration. Chromatographic separation was performed on a Waters Acquity UPLC system interfaced with a Q-Exactive Focus mass spectrometer (Thermo Scientific, Bremen, Germany), using a heated electrospray ionization (HESI-II) source. Thermo Scientific Xcalibur 3.1 software was used for instrument control. The LC conditions were as follows: column, Waters BEH C_18_ 100 × 2.1 mm I.D., 1.7 μm; mobile phase, (A) water with 0.1% formic acid; (B) acetonitrile with 0.1% formic acid; flow rate, 600 μl min^−1^; injection volume, 5 μl; gradient, a linear gradient of 5–100% B over 13.5 min and isocratic at 100% B for 2 min. The optimized HESI-II parameters were as follows: source voltage, 3.5 kV (pos); sheath gas flow rate (N_2_), 55 units; auxiliary gas flow rate, 15 units; spare gas flow rate, 3.0; capillary temperature, 350.00 °C, S-Lens RF level, 45. The mass analyzer was calibrated using a mixture of caffeine, methionine–arginine–phenylalanine–alanine–acetate (MRFA), sodium dodecyl sulfate, sodium taurocholate, and Ultramark 1621 in an acetonitrile/methanol/water solution containing 1% formic acid by direct injection. The data-dependent MS/MS events were performed on the three most intense ions detected in full scan MS (Top3 experiment). The MS/MS isolation window width was 1 Da, and the stepped normalized collision energy was set to 15, 30, and 45 units. In data-dependent MS/MS experiments, full scans were acquired at a resolution of 35 000 FWHM (at *m*/*z* 200) and MS/MS scans at 17 500 FWHM with an automatically determined maximum injection time. After being acquired in an MS/MS scan, parent ions were placed in a dynamic exclusion list for 2.0 s.

### UHPLC-MS/MS data-treatment

The MS data were converted from RAW (Thermo) standard data format to mzML format using the MSConvert software, part of the ProteoWizard package.^55^ The converted files were treated using the MZmine software suite v. 3.1.0.^56,57^ The parameters were adjusted as follows: the centroid mass detector was used for mass detection with the noise level set to 1.0 × 10^5^ for MS level set to 1, and to 0 for MS level set to 2. The ADAP chromatogram builder was used and set to a minimum group size of scans of 5, minimum group intensity threshold of 1.0 × 10^5^, minimum highest intensity of 5.0 × 10^5^ and *m*/*z* tolerance of 10 ppm. For chromatogram deconvolution, the algorithm used was the wavelets (ADAP).^58^ The intensity window S/N was used as S/N estimator with a signal-to-noise ratio set at 15, a minimum feature height at 5.0 × 10^5^, a coefficient area threshold at 110, a peak duration ranges from 0.1 to 0.5 min and the RT wavelet range from 0.02 to 0.06 min. Isotopes were detected using the isotopes peaks grouper with a *m*/*z* tolerance of 15 ppm, a RT tolerance of 0.05 min (absolute), the maximum charge set at 2 and the representative isotope used was the most intense. Individual feature lists were aligned using a RT tolerance of 0.05 min, a *m*/*z* tolerance of 10 ppm, a weight for *m*/*z* of 2 and a weight for RT of 1. At this point, only features with a corresponding MS/MS spectrum, at least 2 features in the ^13^C isotope pattern and a RT between 0.5 and 15.5 min were kept. For IIN, correlated features were grouped using the metaCorrelate module (RT tolerance of 0.05 min, no minimal feature height, and an intensity correlation threshold of 10 000). Finally, adducts were annotated for correlated features using the IIN module with a *m*/*z* tolerance of 10 ppm and no minimal feature height. Files (.mgf spectra file, .csv features quantification table and .csv IIN edges file) were exported for IIFBMN using the export to GNPS – FBMN module.

### Ion-identity molecular networking

Exported files (spectra, feature quantification table and IIN edges) were uploaded on GNPS to perform IIFBMN.^15,36,59^ Parameters were set as follows: precursor ion and fragment mass tolerance were set to 0.02, the modified-cosine score threshold was set to 0.7, the minimum matched fragments ions were set to 6 and the maximum component size was set to 100. The resulting IIFBMN is available at https://gnps.ucsd.edu/ProteoSAFe/status.jsp?task=bef399e77f86411ab6857bce924f4b07.

### Semi-preparative HPLC-PDA purification

To purify the targeted compounds, the chromatographic conditions were first optimized using an HP 1260 Agilent High-Performance liquid chromatography equipped with a photodiode array detector (HPLC-PDA) (Agilent Technologies, Santa Clara, CA, United States). The chromatographic separation was performed on an XBridge C_18_ column (250 × 4.6 mm I.D., 5 μm; Waters) equipped with a C_18_ pre-column at 1 mL min^−1^, with H_2_O (A) and MeCN or MeOH (B) both containing 0.1% formic acid as solvents. MeCN was used for *Cnestis palala* roots, *Chadsia grevei* roots bark, and *Pachyrhizus erosus* leaves extracts, while MeOH was used for *Desmodium heterophyllum* underground parts. The UV absorbance was measured at 254 nm, and the UV-PDA spectra were recorded between 190 and 600 nm (step 2 nm).

The geometrically transferred gradients were used at the semi-preparative scale on a Shimadzu system equipped with an LC-20 A module pumps, an SPD-20 A UV/VIS, a 7725I Rheodyne® valve, and an FRC-40 fraction collector (Shimadzu, Kyoto, Japan). The separation was performed on an XBridge C_18_ column (250 mm × 19 mm I.D., 5 μm; Waters) equipped with a C_18_ precolumn cartridge holder (10 mm × 19 mm I.D., 5 μm; Waters) at 17 mL min^−1^, with H_2_O (A) and MeOH or MeCN (B) (same solvent as analytical HPLC optimization) both containing 0.1% formic acid as solvents. The UV detection was set at 210 and 254 nm. The mixtures were injected on the semi-preparative HPLC column using a dry-load methodology.^35^

#### *Cnestis palala* roots bark extract

The solvents used were H_2_O (A) and MeCN (B), both containing 0.1% formic acid. The optimized gradient was: 40 to 65% B in 60 min, 65 to 100% B in 2 min, followed by an isocratic step at 100% B for 10 min. Three consecutive injections of *ca.* 40 mg of extract were performed and yielded 24 fractions after pooling of corresponding fractions based on the semi-preparative HPLC chromatographic profile. Fraction 7 was identified as compound 22 (0.3 mg, *R*_*t*_ = 11 min), fraction 8 as compound 36 (1.1 mg, *R*_*t*_ = 13 min), fraction 10 as compound 16 (0.7 mg, *R*_*t*_ = 15 min), fraction 12 as compound 16 (0.7 mg, *R*_*t*_ = 19 min), fraction 14 as compound 33 (1.2 mg, *R*_*t*_ = 22 min), fraction 15 as compound 35 (1.5 mg, *R*_*t*_ = 24 min), fraction 17 as compound 38 (2.1 mg, *R*_*t*_ = 30 min), fraction 18 as compound 34 (3.6 mg, *R*_*t*_ = 33 min), and fraction 20 as compound 39 (4.9 mg, *R*_*t*_ = 40 min).

#### *Chadsia grevei* roots extract

The solvents used were H_2_O (A) and MeCN (B), both containing 0.1% formic acid. The optimized gradient was: 10 to 45% B in 10 min, isocratic 45% B for 30 min, 45 to 100% B in 25 min, followed by an isocratic step at 100% B for 10 min. Three consecutive injections of *ca.* 40 mg of extract were performed and yielded 32 fractions. Fraction 1 was identified as compound 20 (0.9 mg, *R*_*t*_ = 16 min), fraction 8 as compound 18 (0.9 mg, *R*_*t*_ = 23 min), fraction 9 as compound 21 (0.6 mg, *R*_*t*_ = 24 min), fraction 14 as compound 40 (1.0 mg, *R*_*t*_ = 31 min), fraction 20 as compound 37 (1.5 mg, *R*_*t*_ = 22 min), fraction 21 as compound 39 (0.4 mg, *R*_*t*_ = 42 min), fraction 23 as compound 41 (1.1 mg, *R*_*t*_ = 54 min), fraction 27 as compound 17 (1.7 mg, *R*_*t*_ = 59 min), fraction 31 as compound 23 (4.0 mg, *R*_*t*_ = 65 min), and fraction 32 as compound 19 (1.3 mg, *R*_*t*_ = 66 min).

#### *Pachyrhizus erosus* leaves extract

The solvents used were H_2_O (A) and MeCN (B), both containing 0.1% formic acid. The optimized gradient was: 25 to 50% B in 75 min, 50 to 100% B in 5 min, followed by an isocratic step at 100% B for 10 min. Three consecutive injections of *ca.* 40 mg of extract were performed and yielded 29 fractions. Fraction 1 was identified as compound 4 (0.3 mg, *R*_*t*_ = 27 min), fraction 2 as compound 24 (0.5 mg, *R*_*t*_ = 28 min), fraction 3 as compound 25 (0.3 mg, *R*_*t*_ = 28 min), fraction 5 as compound 26 (0.4 mg, *R*_*t*_ = 31 min), fraction 6 as compound 31 (0.7 mg, *R*_*t*_ = 34 min), fraction 8 as compound 29 (1.5 mg, *R*_*t*_ = 40 min), fraction 9 as compound 6 (0.2 mg, *R*_*t*_ = 42 min), fraction 10 as compound 30 (13.6 mg, *R*_*t*_ = 43 min), fraction 13 as compound 32 (0.6 mg, *R*_*t*_ = 48 min), fraction 16/17 as compound 5 (1.1 mg, *R*_*t*_ = 54 min), fraction 18 as compound 3 (0.7 mg, *R*_*t*_ = 55 min), fraction 21 as compound 28 (1.8 mg, *R*_*t*_ = 58 min), fraction 22 as compound 2 (1.0 mg, *R*_*t*_ = 59 min), fraction 25 as compound 27 (0.9 mg, *R*_*t*_ = 68 min), fraction 26 as compound 37 (1.7 mg, *R*_*t*_ = 70 min), fraction 28 as compound 1 (0.9 mg, *R*_*t*_ = 73 min), and fraction 29 as compound 7 (1.0 mg, *R*_*t*_ = 79 min).

#### *Desmodium heterophyllum* underground parts extract

The solvents used were H_2_O (A) and MeOH (B), both containing 0.1% formic acid. The optimized gradient was: 50 to 70% B in 60 min, 70 to 100% B in 20 min, followed by an isocratic step at 100% B for 10 min. Three consecutive injections of *ca.* 40 mg of extract were performed and yielded 24 fractions. Fraction 2 was identified as compound 12 (0.6 mg, *R*_*t*_ = 12 min), fraction 8 as compound 13 (0.3 mg, *R*_*t*_ = 23 min), fraction 15 as compound 10 (1.1 mg, *R*_*t*_ = 39 min), fraction 16 as compound 11 (0.3 mg, *R*_*t*_ = 41 min), fraction 17 as compound 37 (2.2 mg, *R*_*t*_ = 42 min), fraction 18 as compound 39 (1.1 mg, *R*_*t*_ = 44 min), fraction 19 as compound 14 (0.8 mg, *R*_*t*_ = 53 min), fraction 23 as compound 8 (3.5 mg, *R*_*t*_ = 68 min), and fraction 24 as compound 9 (1.6 mg, *R*_*t*_ = 72 min).

### Isolated compounds

For details on isolated compounds and structural elucidation, see ESI.†

### Cytotoxicity assay: L-6 cells

The pure compounds cytotoxicity was assessed against L6 cells as described in ref. 27.

### Activity against *Trypanosoma cruzi*

The pure compounds activity was assessed against *T. cruzi* amastigotes as described in ref. 27.

## Conclusion

Applying the ENPKG sample-centric and semantic enrichment methodology to anti-parasitic screening results and the associated metabolomics dataset of 1600 plant extracts, we quickly identified rotenoids as being responsible for anti-*T. cruzi* activity. These derivatives were located in six different plant extracts from four different botanical species: *Desmodium heterophyllum*, *Chadsia grevei*, *Pachyrhizus erosus*, and *Cnestis palala*. Deguelin or rotenone, two highly active rotenoids, were identified in all six active extracts. Using IIN-FBMN, we found several rotenoid analogs in active extracts from the four botanical species considered. We targeted the isolation of these potentially active analogs using a streamlined single chromatographic-step semi-preparative HPLC procedure. This resulted in a library of 41 compounds, seven of which, to our knowledge, have not been previously reported. Their bioactivity was assessed against intracellular *T. cruzi* amastigotes, and only tephrosin displayed an activity range similar to deguelin and rotenone. In this work, we demonstrate how modern annotation strategies and mining tools, coupled with state-of-the-art isolation and structural characterization techniques, can help to efficiently generate a library of structural analogs for bioactivity assessment. While only having limited amounts of botanical material and isolated compounds present certain difficulties for complete and detailed structural analysis, the strategy enables fast deconvolution of the compounds responsible for the bioactivity of the crude extracts. This method allows for identification of metabolites responsible for bioactivity in individual extracts, and in the entire NE library, thanks to the connection possible through ENPKG and its multimodal alignment.^27^ As showcased in this study, and since plants often produce various structurally related scaffolds, the compounds obtained can be used for structure–activity relationship evaluation. This workflow demonstrates how modern NP research methods can facilitate the transition from an NE library to a digitalized NP library. Together with recent advances in computational metabolomics and data analysis, these advances show how these tools can allow researchers to efficiently explore the potential for natural products drug discovery.

## Data availability

The ENPKG containing data information of the original screening on 1600 plant extracts is available at https://enpkg.commons-lab.org/graphdb/. The IIFBMN obtained from the newly generated extracts of selected species is available at https://gnps.ucsd.edu/ProteoSAFe/status.jsp?task=bef399e77f86411ab6857bce924f4b07.

## Author contributions

Conceptualization: AGa, P-MA and J-LW. Data curation: AGa, MK and LM. Funding acquisition: J-RI, J-LW and P-MA. Investigation: AGa, JF, MK, LM, EFQ and P-MA. Project administration: J-LW and P-MA. Resources: MK, PM, J-RI, BD, AGr and J-LW. Supervision: J-LW and P-MA. Visualization: AGa. Writing – original draft: AGa. Writing – review & editing: AGa, LM, MK, PM, JR, AGr, BD, J-RI, EFQ, J-LW and P-MA.

## Conflicts of interest

The authors declare that the research was conducted without any commercial or financial relationships that could be construed as a potential conflict of interest.

## Supplementary Material

RA-015-D4RA08652J-s001

## References

[cit1] Pérez-Molina J. A., Molina I. (2018). Chagas disease. Lancet.

[cit2] Field M. C., Horn D., Fairlamb A. H., Ferguson M. A., Gray D. W., Read K. D. (2017). *et al.*, Anti-trypanosomatid drug discovery: an ongoing challenge and a continuing need. Nat. Rev. Microbiol..

[cit3] Centers for Disease Control and Prevention , CDC – Chagas Disease, 2009, https://www.cdc.gov/parasites/chagas/biology.html

[cit4] World Health Organization , Chagas disease, https://www.who.int/news-room/fact-sheets/detail/chagas-disease-(american-trypanosomiasis)

[cit5] Mejia A. M., Hall B. S., Taylor M. C., Gómez-Palacio A., Wilkinson S. R., Triana-Chávez O. (2012). *et al.*, Benznidazole-resistance in Trypanosoma cruzi is a readily acquired trait that can arise independently in a single population. J. Infect. Dis..

[cit6] Martín-Escolano J., Medina-Carmona E., Martín-Escolano R. (2020). Chagas Disease: Current View of an Ancient and Global Chemotherapy Challenge. ACS Infect. Dis..

[cit7] Wang J., Xu C., Wong Y. K., Li Y., Liao F., Jiang T. (2019). *et al.*, Artemisinin, the magic drug discovered from traditional Chinese medicine. Engineering.

[cit8] Feher M., Schmidt J. M. (2003). Property Distributions: Differences between Drugs, Natural Products, and Molecules from Combinatorial Chemistry. J. Chem. Inf. Comput. Sci..

[cit9] Clemons P. A., Bodycombe N. E., Carrinski H. A., Wilson J. A., Shamji A. F., Wagner B. K. (2010). *et al.*, Small molecules of different origins have distinct distributions of structural complexity that correlate with protein-binding profiles. Proc. Natl. Acad. Sci. U. S. A..

[cit10] Bindseil K. U., Jakupovic J., Wolf D., Lavayre J., Leboul J., van der Pyl D. (2001). Pure compound libraries; a new perspective for natural product based drug discovery. Drug Discovery Today.

[cit11] Sukuru S. C. K., Jenkins J. L., Beckwith R. E. J., Scheiber J., Bender A., Mikhailov D. (2009). *et al.*, Plate-based diversity selection based on empirical HTS data to enhance the number of hits and their chemical diversity. J. Biomol. Screening.

[cit12] Stratton C. F., Newman D. J., Tan D. S. (2015). Cheminformatic comparison of approved drugs from natural product *versus* synthetic origins. Bioorg. Med. Chem. Lett..

[cit13] David B., Grondin A., Schambel P., Vitorino M., Zeyer D. (2020). Plant natural fragments, an innovative approach for drug discovery. Phytochem. Rev..

[cit14] Wolfender J.-L., Litaudon M., Touboul D., Queiroz E. F. (2019). Innovative omics-based approaches for prioritisation and targeted isolation of natural products - new strategies for drug discovery. Nat. Prod. Rep..

[cit15] Wang M., Carver J. J., Phelan V. V., Sanchez L. M., Garg N., Peng Y. (2016). *et al.*, Sharing and community curation of mass spectrometry data with GNPS. Nat. Biotechnol..

[cit16] Allard P.-M., Péresse T., Bisson J., Gindro K., Marcourt L., Pham V. C. (2016). *et al.*, Integration of Molecular Networking and In-Silico MS/MS Fragmentation for Natural Products Dereplication. Anal. Chem..

[cit17] Duhrkop K., Fleischauer M., Ludwig M., Aksenov A. A., Melnik A. V., Meusel M. (2019). *et al.*, SIRIUS 4: a rapid tool for turning tandem mass spectra into metabolite structure information. Nat. Methods.

[cit18] Duhrkop K., Shen H., Meusel M., Rousu J., Bocker S. (2015). Searching molecular structure databases with tandem mass spectra using CSI:FingerID. Proc. Natl. Acad. Sci. U. S. A..

[cit19] Dührkop K., Nothias L.-F., Fleischauer M., Reher R., Ludwig M., Hoffmann M. A. (2021). *et al.*, Systematic classification of unknown metabolites using high-resolution fragmentation mass spectra. Nat. Biotechnol..

[cit20] Olivon F., Allard P.-M., Koval A., Righi D., Genta-Jouve G., Neyts J. (2017). *et al.*, Bioactive Natural Products Prioritization Using Massive Multi-informational Molecular Networks. ACS Chem. Biol..

[cit21] Nothias L.-F., Nothias-Esposito M., da Silva R., Wang M., Protsyuk I., Zhang Z. (2018). *et al.*, Bioactivity-Based Molecular Networking for the Discovery of Drug Leads in Natural Product Bioassay-Guided Fractionation. J. Nat. Prod..

[cit22] Kurita K. L., Glassey E., Linington R. G. (2015). Integration of high-content screening and untargeted metabolomics for comprehensive functional annotation of natural product libraries. Proc. Natl. Acad. Sci. U. S. A..

[cit23] Allard P.-M., Gaudry A., Quirós-Guerrero L.-M., Rutz A., Dounoue-Kubo M., Walker T. W. N. (2022). *et al.*, Open and reusable annotated mass spectrometry dataset of a chemodiverse collection of 1,600 plant extracts. GigaScience.

[cit24] Gaudry A., Huber F., Nothias L.-F., Cretton S., Kaiser M., Wolfender J.-L. (2022). *et al.*, MEMO: Mass Spectrometry-Based Sample Vectorization to Explore Chemodiverse Datasets. Front. Bioinform..

[cit25] Cretton S., Dorsaz S., Azzollini A., Favre-Godal Q., Marcourt L., Ebrahimi S. N. (2016). *et al.*, Antifungal Quinoline Alkaloids from Waltheria indica. J. Nat. Prod..

[cit26] Cretton S., Breant L., Pourrez L., Ambuehl C., Marcourt L., Ebrahimi S. N. (2014). *et al.*, Antitrypanosomal quinoline alkaloids from the roots of Waltheria indica. J. Nat. Prod..

[cit27] Gaudry A., Pagni M., Mehl F., Moretti S., Quiros-Guerrero L.-M., Cappelletti L. (2024). *et al.*, A sample-centric and knowledge-driven computational framework for natural products drug discovery. ACS Cent. Sci..

[cit28] Cretton S., Bréant L., Pourrez L., Ambuehl C., Perozzo R., Marcourt L. (2015). *et al.*, Chemical constituents from Waltheria indica exert *in vitro* activity against Trypanosoma brucei and *T. cruzi*. Fitoterapia.

[cit29] Cretton S., Kaiser M., Karimou S., Ebrahimi S. N., Mäser P., Cuendet M. (2020). *et al.*, Pyridine-4(1*H*)-one Alkaloids from Waltheria indica as Antitrypanosomatid Agents. J. Nat. Prod..

[cit30] Dilrukshi Herath H. M. P., Preston S., Hofmann A., Davis R. A., Koehler A. V., Chang B. C. H. (2017). *et al.*, Screening of a small, well-curated natural product-based library identifies two rotenoids with potent nematocidal activity against Haemonchus contortus. Vet. Parasitol..

[cit31] Upegui Y., Gil J. F., Quinones W., Torres F., Escobar G., Robledo S. M. (2014). *et al.*, Preparation of rotenone derivatives and *in vitro* analysis of their antimalarial, antileishmanial and selective cytotoxic activities. Molecules.

[cit32] Kim H. W., Wang M., Leber C. A., Nothias L.-F., Reher R., Kang K. B. (2021). *et al.*, NPClassifier: A Deep Neural Network-Based Structural Classification Tool for Natural Products. J. Nat. Prod..

[cit33] Ligor M., Studzińska S., Horna A., Buszewski B. (2013). Corona-Charged Aerosol Detection: An Analytical Approach. Crit. Rev. Anal. Chem..

[cit34] Rutz A., Wolfender J.-L. (2023). Automated composition assessment of natural extracts: Untargeted mass spectrometry-based metabolite profiling integrating semiquantitative detection. J. Agric. Food Chem..

[cit35] Queiroz E. F., Alfattani A., Afzan A., Marcourt L., Guillarme D., Wolfender J. L. (2019). Utility of dry load injection for an efficient natural products isolation at the semi-preparative chromatographic scale. J. Chromatogr. A.

[cit36] Schmid R., Petras D., Nothias L.-F., Wang M., Aron A. T., Jagels A. (2021). *et al.*, Ion identity molecular networking for mass spectrometry-based metabolomics in the GNPS environment. Nat. Commun..

[cit37] Manjary F., Petitjean A., Conan J. Y., Martin M. T., Frappier F., Rasoanaivo P. (1994). *et al.*, Degueline: A Rotenoid Constituent of Chadsia grevei. Planta Med..

[cit38] Estrella-Parra E. A., Gomez-Verjan J. C., González-Sánchez I., Vázquez-Martínez E. R., Vergara-Castañeda E., Cerbón M. A. (2014). *et al.*, Rotenone isolated from Pachyrhizus erosus displays cytotoxicity and genotoxicity in K562 cells. Nat. Prod. Res..

[cit39] Norton L. B., Hansberry R. (1945). Constituents of the Insecticidal Resin of the Yam Bean (Pachyrrhizus erosus). J. Am. Chem. Soc..

[cit40] Krishnamurti M., Sambhy Y. R., Seshadri T. R. (1970). Chemical study of indian yam beans (Pachyrrhizus erosus): Isolation of two new rotenoids: 12a-hydroxydolineone and 12a-hydroxypachyrrhizone. Tetrahedron.

[cit41] Phrutivorapongkul A., Lipipun V., Ruangrungsi N., Watanabe T., Ishikawa T. (2002). Studies on the constituents of seeds of Pachyrrhizus erosus and their anti herpes simplex virus (HSV) activities. Chem. Pharm. Bull..

[cit42] Veitch N. C. (2009). Isoflavonoids of the leguminosae. Nat. Prod. Rep..

[cit43] Caboni P., Sherer T. B., Zhang N., Taylor G., Na H. M., Greenamyre J. T. (2004). *et al.*, Rotenone, deguelin, their metabolites, and the rat model of Parkinson's disease. Chem. Res. Toxicol..

[cit44] Lümmen P. (1998). Complex I inhibitors as insecticides and acaricides. Biochim. Biophys. Acta.

[cit45] Schiller J., Zickermann V. (2022). Binding of natural inhibitors to respiratory complex I. Pharmaceuticals.

[cit46] Ueno H., Miyoshi H., Ebisui K., Iwamura H. (1994). Comparison of the inhibitory action of natural rotenone and its stereoisomers with various NADH-ubiquinone reductases. Eur. J. Biochem..

[cit47] Ueno H., Miyoshi H., Inoue M., Niidome Y., Iwamura H. (1996). Structural factors of rotenone required for inhibition of various NADH-ubiquinone oxidoreductases. Biochim. Biophys. Acta.

[cit48] Miyoshi H. (1998). Structure-activity relationships of some complex I inhibitors. Biochim. Biophys. Acta.

[cit49] Pereira C. S., Teixeira M. H., Russell D. A., Hirst J., Arantes G. M. (2023). Mechanism of rotenone binding to respiratory complex I depends on ligand flexibility. Sci. Rep..

[cit50] Fang N., Casida J. E. (1999). Cubé resin insecticide: Identification and biological activity of 29 rotenoid constituents. J. Agric. Food Chem..

[cit51] Hernandez F. R., Turrens J. F. (1998). Rotenone at high concentrations inhibits NADH-fumarate reductase and the mitochondrial respiratory chain of Trypanosoma brucei and T. cruzi. Mol. Biochem. Parasitol..

[cit52] Carranza J. C., Kowaltowski A. J., Mendonça M. A. G., de Oliveira T. C., Gadelha F. R., Zingales B. (2009). Mitochondrial bioenergetics and redox state are unaltered in Trypanosoma cruzi isolates with compromised mitochondrial complex I subunit genes. J. Bioenerg. Biomembr..

[cit53] Liu Z., Ulrich vonBargen R., McCall L.-I. (2021). Central role of metabolism in Trypanosoma cruzi tropism and Chagas disease pathogenesis. Curr. Opin. Microbiol..

[cit54] European Commission , Official European Commission register of collections, 2020, https://ec.europa.eu/environment/nature/biodiversity/international/abs/pdf/Register%20of%20Collections.pdf

[cit55] Chambers M. C., Maclean B., Burke R., Amodei D., Ruderman D. L., Neumann S. (2012). *et al.*, A cross-platform toolkit for mass spectrometry and proteomics. Nat. Biotechnol..

[cit56] Pluskal T., Castillo S., Villar-Briones A., Orešič M. (2010). MZmine 2: Modular framework for processing, visualizing, and analyzing mass spectrometry-based molecular profile data. BMC Bioinf..

[cit57] Schmid R., Heuckeroth S., Korf A., Smirnov A., Myers O., Dyrlund T. S. (2023). *et al.*, Integrative analysis of multimodal mass spectrometry data in MZmine 3. Nat. Biotechnol..

[cit58] Myers O. D., Sumner S. J., Li S., Barnes S., Du X. (2017). One Step Forward for Reducing False Positive and False Negative Compound Identifications from Mass Spectrometry Metabolomics Data: New Algorithms for Constructing Extracted Ion Chromatograms and Detecting Chromatographic Peaks. Anal. Chem..

[cit59] Nothias L.-F., Petras D., Schmid R., Dührkop K., Rainer J., Sarvepalli A. (2020). *et al.*, Feature-based molecular networking in the GNPS analysis environment. Nat. Methods.

