# Supplementary material for: Efficient constitution of a library of rotenoid analogs active against Trypanosoma cruzi from a digitalized plant extract collection
Source: RSC Adv. 2025 May 9;15(19):15240–51. doi: 10.1039/d4ra08652j (PMC12062876; doi:10.1039/d4ra08652j)
Supplement: RA-015-D4RA08652J-s001 [file RA-015-D4RA08652J-s001.pdf]

Supplementary Information (SI) for RSC Advances.
This journal is © The Royal Society of Chemistry 2025

Supplementary Information

Structural Elucidation of New Compounds

Due to the limited quantities obtained during the purification process in a single chromatographic step, mainly to allow the biological activity of the extracts to be deconvoluted, structural elucidation was carried out as extensively as possible. Among the unreported compounds, compound **33** is the only rotenoid. Compound **33** was isolated as an amorphous powder with a $[M-C_2H_4O_2+H]^+$ of *m/z* 353.1012 and a molecular formula of $C_{22}H_{20}O_8$. NMR data show that this compound is a member of the rotenoid family and, in particular, bears a close resemblance to that of 12-deoxo-12α-acetoxyelliptone (**34**). The ^{1}H-NMR and HSQC spectra indicated the presence of three methyl groups: two methoxyl (δ_H 3.85, δ_C 56.1, 3'-OCH_3 and δ_H 3.87, δ_C 56.5, 2'-OCH_3) and one acetoxy group (δ_H 1.78, s, δ_C 20.9, 12-OAc), this latter was not observed in MS because of in-source fragmentation. Four aromatic protons are also visible, in the form of two singulets (δ_H 6.39, δ_C 99.9 and 7.07, δ_C 108.6, CH-4 and CH-1 respectively) and two ortho protons (*J* = 8.5 Hz) at δ_H 7.16, δ_C 106.0 for CH-10 and δ_H 7.22, δ_C 127.0 for CH-11. Also, two benzofuranic protons were observed at δ_H 6.88, δ_C 104.2, for CH-3' and δ_H 7.58, δ_C 144.6, for CH-2'. The HSQC spectrum indicated the presence of an oxymethylene (δ_H 4.42, dd, *J* = 10.7, 5.4 Hz, and 4.67, dd, 11.3, 10.7 Hz, δ_C 64.7, CH_2-6) and two oxymethines: one in the form of a doublet of doublet of doublets (δ_H 4.86, ddd, *J* = 11.3, 5.4, 1.8 Hz, δ_C 75.4, CH-6a) and one in the form of a doublet (δ_H 6.27, d, *J* = 1.8 Hz, δ_C 70.4, CH-12). Finally, a singlet was observed at δ_H 2.39 on the ^{1}H NMR spectrum recorded in $CDCl_3$. It was assigned to a hydroxy group and positioned in C-12a thanks to its HMBC to C-12a (δ_C 69.1), C-12, and C-1a (δ_C 112.6). The relative configuration between CH-6a, CH-12, and OH-12a was established based on the ROESY correlations observed from H-6a to OH-12a and H-12 and from OH-12a to H-12 and H-1. The ^4J W-coupling of 1.8 Hz between H-6a and H-12 confirms the equatorial position of these protons. The absolute configuration of **33** could not be determined based on the data obtained. Compound **33** was identified as 12-deoxo-12α-acetoxy-12aβ-hydroxyelliptone.

Compound **1** was isolated as an amorphous powder with a $[M+H]^+$ of *m/z* 341.1742 and a molecular formula of $C_{21}H_{24}O_4$. The ^{1}H-NMR spectrum indicated that **1** was a close analog of bavaisoflavanol (**2**) and manuifolin H (**3**) . When compared to manuifolin H, an additional methoxy signal is observed at δ_H 3.80 (δ_C 55.8, 2'-OCH_3), confirming the information obtained from the HRMS. The ROESY correlation between 2'-OCH_3 and H-3' (δ_H 6.44, d, *J* = 2.6 Hz) and the HMBC correlation from 2'-OCH_3 to C-2' (δ_C 159.6) and from H-6' (δ_H 6.92, d, *J* = 8.3 Hz) to C-2' allowed to determine the position of the methoxyl in C-2'. The absolute configuration of **1** was deduced as 3*R* based on the negative optical rotation value ($[\alpha]_D^{20}$ -70.5), which was on the same sign as that of manuifolin H (**3**). Compound **1** was identified as 2'-*O*-methylmanuifolin H.

Compound **6** was isolated as an amorphous powder with a $[M+H]^+$ of *m/z* 357.1700 and a molecular formula of $C_{21}H_{24}O_5$. The NMR data of **6** indicated a structure analog to 2'-*O*-methylmanuifolin H (**1)**, except for the allylic group. The two methyl groups are still observed (δ_H 1.21, s, H_3-12) and (δ_H 1.23, s, H_3-13) but with a slightly higher field than those of **1**. The HMBC spectrum showed that the carbon atom bearing the two methyl groups (C-11) was shifted at a higher field (δ_C 72.5) compared to the one of **1** (δ_C 132.3). The same shielded chemical shift could be observed for CH-10 (δ_C 90.9) compared to the one of **1** (δ_C 124.6) and indicates the loss of the olefin group. In addition, the chemical shifts of CH-10 and C-11 indicated that both atoms were bearing an oxygen atom. Because of the obtained molecular formula, an ether link should arise between C-10 and C-7 or between C-11 and C-7. A comparison of the chemical shift values of H-10, C-10, and C-11 with those of rautandiol A which contain a 3-hydroxy-2,2dimethyldihydropyran moiety and B which contain a hydroxyisopropyldihydrofuran moiety [1] indicated that these values are closer to those of rautandiol B, and thus an ether between C-10 and C-7 occurred. Because of the distance between the two chiral carbon atoms CH-3 and CH-10, the relative configuration of **6** could not be established based on the data obtained. Compound **6** was identified as 4-(2-(2-hydroxypropan-2-yl)-2,3,6,7-tetrahydro-5H-furo[3,2-g]chromen-6-yl)-3-methoxyphenol.

Compound **13** was isolated with an $[M+H]^+$ of *m/z* 397.1648 corresponding to a molecular formula of $C_{23}H_{24}O_6$. The NMR data of **13** showed the same signals as those obtained for the flavanone part of purpurine (**11**): a hydroxy methine at δ_H 5.56 (dd, *J* = 13.8, 3.0 Hz, H-2), a methylene at δ_H 2.85 (dd, *J* = 17.0, 3.0 Hz, H-3b) and 2.96 (dd, *J* = 17.0, 13.8 Hz, H-3a), phenyl protons at δ_H 7.38 (t, *J* = 7.7 Hz, H-4'), 7.45 (t, *J* = 7.7 Hz, H-3', H-5'), and 7.61 (d, *J* = 7.7 Hz, H-2', H-6'), and two ortho coupled aromatics at δ_H 6.54 (d, *J* = 8.4 Hz, H-6) and 7.81 (d, *J* = 8.4 Hz, H-5). The signals for the ditetrahydrofuran part of purpurine are different in **13**. In this latter, they consist of two methyl groups at δ_H 1.22 and 1.23 linked to quaternary carbon bearing an oxygen atom (δ_C 73.0, C-5''), a methine bearing an acetoxy group at δ_H 4.94 and 1.88 for the acetate, a methine at δ_H 3.86 (t, *J* = 7.7 Hz, H-3'') and a methylene at δ_H 4.62 (t, *J* = 8.8 Hz, H-2''b) and 4.82 (overlapped, H-2''a). This information indicated that the dihydrofurane of purpurin was open in **13** between the oxygen linked to C-5'' and C-2''. The HMBC correlations are in agreement with this structure. Due to the opening of the dihydrofuran ring, the relative configuration of **13** could not be established. Based on these data, the structure of **13** was established as 2-hydroxy-2-methyl-1-(4-oxo-2-phenyl-3,4,8,9-tetrahydro-2H-furo[2,3-h]chromen-9-yl) propyl acetate.

Compound **12** was isolated with an $[M+H]^+$ *m/z* 413.1586 corresponding to a molecular formula of $C_{23}H_{24}O_7$. The NMR data of **12** showed close similarities to those of **13** except for the phenyl group, which appeared in **12** as a para-substituted phenol as indicated by the four

aromatic protons at δ_H 6.84 (d, *J* = 8.5 Hz, H-3', H-5'), and 7.41 (2H, d, *J* = 8.5 Hz, H-2', H-6'). This structure is consistent with the HRMS data. Based on this data, the structure of **12** was established as 2-hydroxy-1-(2-(4-hydroxyphenyl)-4-oxo-3,4,8,9-tetrahydro-2H-furo[2,3-h]chromen-9-yl)-2-methylpropyl acetate. As for compound **13**, the relative configuration of **12** could not be established.

Compound **14** was isolated with an $[M+H]^+$ of *m/z* 485.1964 corresponding to a molecular formula of $C_{30}H_{28}O_6$. The HRMS data of **14** indicated a C_7H_4 increment compared with **13**. Compared to **13**, the ^{1}H-NMR spectrum indicated that the acetyl signal was missing and that the signals corresponding to a styrene group were observed at δ_H 6.31 (d, *J* = 16.0 Hz, H-9'') and 7.46 (m, H-10”) for the vinyl and δ_H 7.39 for the phenyl protons. The HMBC correlations from H4”, H-9”, and H-10” to C-8” (δ_H 167.4) allowed us to link this group to C-4”. Based on this data, the structure of **14** was established as 2-hydroxy-2-methyl-1-(4-oxo-2-phenyl-3,4,8,9-tetrahydro-2H-furo[2,3-h]chromen-9-yl)propyl cinnamate. As for compounds **12** and **13**, the relative configuration of **14** could not be established based on the data obtained.

Compound **21** was isolated as an amorphous powder with a $[M+H]^+$ of *m/z* 353.1020 and a molecular formula of $C_{20}H_{16}O_6$. The NMR and HRMS data of **21** showed that when compared to glabrone (**20**) ($C_{20}H_{16}O_5$), an additional phenol group positioned in C-6 was observed. This is confirmed by the presence of two aromatic singlets at δ_H 6.91 and 7.49 for H-8 and H-5, respectively. Based on this data, the structure of **21** was established as 5',6,7-trihydroxy-2',2'-dimethyl-2'H,4H-[3,6'-bichromen]-4-one. Compound **21** was named 6-hydroxy-glabrone.

Table S1: ^{1}H-NMR ($CDCl_3$, 600 MHz) and ^{13}C-NMR ($CDCl_3$, 151 MHz) data of compounds **33**

	**33 ($CDCl_3$)**	
Position	δ_H (J in Hz)	δ_C, Type
**1**	7.07 (s)	108.6, CH
**1a**	-	112.6, C
**2**	-	144.1, C
**3**	-	150.6, C
**4**	6.39 (s)	99.9, CH
**4a**	-	148.8, C
**6**	4.42 (dd, 10.7, 5.4 Hz), 4.67 (dd, 11.3, 10.7 Hz)	64.7, CH_2
**6a**	4.86 (ddd, 11.3, 5.4, 1.8 Hz)	75.4, CH
**7a**	-	146.1, C
**8**	-	117.1, C
**9**	-	157.0, C
**10**	7.16 (dd, 8.5, 0.9 Hz)	106.0, CH
**11**	7.22 (d, 8.5 Hz)	127.0, CH
**11a**	-	110.3, C
**12**	6.27 (d, 1.8 Hz)	70.4, CH
**12a**	2.39 (s, OH)	69.1, C
**2-OCH_3**	3.87 (s)	56.5, CH_3
**3-OCH_3**	3.85 (s)	56.1, CH_3
**3'**	6.88 (dd, 2.2, 0.9 Hz)	104.2, CH
**2'**	7.58 (d, 2.2 Hz)	144.6, CH
**12-OAc**	-	169.7, C
**12-OAc**	1.78 (s)	20.9, CH_3

Table S2: ^{1}H-NMR (CD_3OD, 600 MHz) and ^{13}C-NMR (CD_3OD, 151 MHz) data of compounds **20** and **21**

	**20 (glabrone)**		**21**	
Position	δ_H (J in Hz)	δ_C, Type	δ_H (J in Hz)	δ_C, Type
**2**	8.00 (s)	156.2, CH	8.20 (s)	156.0, CH
**3**	-	*NO*	-	123.9, C
**4**	-	178.3, C	-	178.9, C
**5**	8.04 (d, 8.8 Hz)	128.5, CH	7.49 (s)	107.8, CH
**6**	6.94 (m)	116.3, CH	-	146.8 , C
**7**	-	164.4, C	-	154.0, C
**8**	6.87 (d, 2.3 Hz)	103.3, CH	6.91 (s)	102.9, CH
**9**	-	159.8, C	-	153.8, C
**10**	-	*NO*	-	*NO*
**1'**	-	112.5, C	-	114.5, C
**2'**	-	154.5, C	-	152.9, C
**3'**	-	110.9, C	-	112.4, C
**4'**	-	153.1, C	-	155.6, C
**5'**	6.40 (d, 8.3 Hz)	108.3, CH	6.38 (d, 8.4 Hz)	109.5, CH
**6'**	6.94 (m)	132.3, CH	6.98 (d, 8.4 Hz)	130.8, CH
**1''**	6.68 (d, 10.0 Hz)	118.2, CH	6.77 (d, 10.0 Hz)	118.2, CH
**2''**	5.60 (d, 10.0 Hz)	129.6, CH	5.65 (d, 10.0 Hz)	129.6, CH
**3''**	-	77.1, C	-	76.7, C
**4''**	1.35 (s)	28.0, CH_3	1.41 (s)	27.8, CH_3
**5''**	1.35 (s)	28.0, CH_3	1.41 (s)	27.8, CH_3

Table S3: ^{1}H-NMR (CD_3OD, 600 MHz) and ^{13}C-NMR (CD_3OD, 151 MHz) data of compounds **3**, **2**, **1** and **6**

	**3 (manuifolin H)**		**2 (bavaisoflavanol)**		**1**		**6**	
Position	δ_H (J in Hz)	δ_C, Type	δ_H (J in Hz)	δ_C, Type	δ_H (J in Hz)	δ_C, Type	δ_H (J in Hz)	δ_C, Type
**2**	3.89 (t, 10.1 Hz), 4.18 (dt, 10.1, 2.9 Hz)	71.2, CH_2	3.47 (m), 4.17 (dd, 9.2, 2.4 Hz)	67.7, CH_2	3.87 (td, 10.0, 2.8 Hz), 4.14 (dd, 10.0, 2.7 Hz)	71.2, CH_2	3.90 (t, 10.4 Hz), 4.16 (dt, 10.4, 2.4 Hz)	71.2, CH_2
**3**	3.40 (m)	33.2, CH	3.47 (m)	41.0, CH	-	33.0, CH	-	32.9, CH
**4**	2.73 (ddd, 15.6, 5.3, 1.9 Hz), 2.90 (dd, 15.6, 11.0 Hz)	31.5, CH_2	5.40 (d, 5.7 Hz),	80.2, CH	2.71 (dd, 15.0, 4.8 Hz), 2.87 (ddd, 15.0, 10.9, 2.7 Hz)	31.5, CH_2	2.77 (dd, 15.5, 5.4 Hz), 2.91 (dd, 15.5, 10.7 Hz)	32.0, CH_2
**4a**	-	114.6, C	-	112.4, C	-	114.4, C	-	115.2, C
**5**	6.70 (s)	131.0, CH	7.11 (s)	132.4, CH	6.69 (s)	130.9, CH	6.84 (s)	126.2, CH
**6**	-	121.7, C	-	123.6, C	-	121.8, C	-	120.5, C
**7**	-	154.8, C	-	157.5, C	-	154.9, C	-	160.5, C
**8**	6.21 (s)	103.5, CH	6.30 (s)	103.5, CH	6.21 (s)	103.5, CH	6.16 (s)	98.2, CH
**8a**	-	154.2, C	-	155.9, C	-	154.1, C	-	155.4, C
**9**	3.19 (d, 7.3 Hz)	28.6, CH_2	3.24 (d, 7.5 Hz)	28.6, CH_2	3.18 (d, 7.4 Hz)	28.6, CH_2	3.06 (m)	30.9, CH_2
**10**	5.29 (t, 7.3 Hz)	124.7, CH	5.33 (t, 7.5 Hz)	124.1, CH	5.28 (t, 7.4 Hz)	124.6, CH	4.54 (t, 8.8 Hz)	90.9 , CH
**11**	-	132.3, C	-	132.9, C	-	132.3, C	-	72.5, C
**12**	1.70 (s)	17.8, CH_3	1.73 (s)	17.8, CH_3	1.69 (s)	17.8, CH_3	1.21 (s)	25.1, CH_3
**13**	1.72 (s)	26.0, CH_3	1.76 (s)	26.0, CH_3	1.72 (s)	25.9, CH_3	1.23 (s)	25.3, CH_3
**1’**	-	120.3, C	-	119.7, C	-	121.7, C	-	121.7, C
**2'**	-	157.2, C	-	161.9, C	-	159.6, C	-	159.6, C
**3'**	6.31 (d, 2.4 Hz)	103.5, CH	6.26 (d, 2.3 Hz)	98.7, CH	6.44 (d, 2.6 Hz)	99.9, CH	6.44 (d, 2.4 Hz)	100.0, CH
**4'**	-	157.9, C	-	159.7, C	-	158.4, C	-	158.5, C
**5'**	6.25 (dd, 8.3, 2.4 Hz)	107.6, CH	6.32 (dd, 8.2, 2.3 Hz)	108.6, CH	6.34 (dd, 8.3, 2.6 Hz)	108.0, CH	6.34 (dd, 8.3, 2.4 Hz)	108.0, CH
**6'**	6.86 (d, 8.3 Hz)	128.8, CH	7.06 (d, 8.2 Hz	126.0, CH	6.92 (d, 8.3 Hz)	128.6, CH	6.92 (d, 8.3 Hz)	128.7, CH
**$-OCH_3$**	-	-	-	-	3.80 (s)	55.8, CH_3	3.80 (s)	55.9, CH_3

Table S4: ^{1}H-NMR (CD_3OD, 600 MHz) and ^{13}C-NMR (CD_3OD, 151 MHz) data of compounds **11**, **13**, **12** and **14**

	**11 ((-)-purpurin)**		**13**		**12**		**14**	
Position	δ_H (J in Hz)	δ_C, Type	δ_H (J in Hz)	δ_C, Type	δ_H (J in Hz)	δ_C, Type	δ_H (J in Hz)	δ_C, Type
**2**	5.69 (dd, 11.7, 3.4 Hz)	81.0, CH	5.56 (dd, 13.8, 3.0 Hz)	81.0, CH	5.44 (dd, 13.8, 2.8 Hz)	81.0, CH	5.44 (dd, 12.2, 3.2 Hz)	80.7, CH
**3**	2.93 (dd, 16.0, 3.4 Hz), 3.06 (dd, 16.0, 11.7 Hz)	45.2, CH_2	2.85 (dd, 17.0, 3.0 Hz), 2.96 (dd, 17.0, 13.8 Hz)	45.8, CH_2	2.79 (dd, 17.0, 2.8 Hz), 2.99 (dd, 17.0, 13.8 Hz)	45.5, CH_2	2.73 (dd, 16.9, 3.2 Hz), 2.90 (dd,16.9, 12.2 Hz)	44.8, CH_2
**4**	-	192.4, C	-	192.5, C	-	193.0, C	-	192.5, C
**5**	7.85 (d, 8.6 Hz)	131.2, CH	7.81 (d, 8.4 Hz)	130.8, CH	7.79 (d, 8.5 Hz)	130.8, CH	7.73 (d, 8.6 Hz)	130.9, CH
**6**	6.56 (d, 8.6 Hz)	105.8, CH	6.54 (d, 8.4 Hz)	105.9, CH	6.52 (d, 8.5 Hz)	105.8, CH	6.53 (d, 8.6 Hz)	106.0, CH
**7**	-	167.1, C	-	170.4, C	-	169.9, C	-	170.3, C
**8**	-	114.6, C	-	115.9, C	-	116.3, C	-	115.6, C
**9**	-	159.8, C	-	161.4, C	-	161.7, C	-	161.1, C
**10**	-	117.0, C	-	116.3, C	-	NO	-	116.5, C
**1'**	-	140.6, C	-	140.7, C	-	131.3, C	-	140.4, C
**2'**	7.52 (d, 8.0 Hz)	127.1, CH	7.61 (d, 7.7 Hz)	127.0, CH	7.41 (d, 8.5 Hz)	128.7, CH	7.46 (m)	126.8, CH
**3'**	7.41 (t, 8.0 Hz)	129.7, CH	7.45 (t, 7.7 Hz)	129.7, CH	6.84 (d, 8.5 Hz)	116.3, CH	7.39 (m)	129.8, CH
**4'**	7.35 (t, 8.0 Hz)	129.5, CH	7.38 (t, 7.7 Hz)	129.5, CH	-	159.2, C	7.39 (m)	129.4, CH
**5'**	7.41 (t, 8.0 Hz)	129.7, CH	7.45 (t, 7.7 Hz)	129.7, CH	6.84 (d, 8.5 Hz)	116.3, CH	7.39 (m)	129.8, CH
**6'**	7.52 (d, 8.0 Hz)	127.1, CH	7.61 (d, 7.7 Hz)	127.0, CH	7.41 (d, 8.5 Hz)	128.7, CH	7.46 (m)	126.8, CH
**2''**	6.52 (d, 6.7 Hz)	113.9, CH	4.62 (t, 8.8 Hz), 4.82 (overlapped)	80.0, CH_2	4.62 (t, 9.4, 8.0 Hz), 4.83 (overlapped)	80.0, CH_2	4.64 (t, 9.4, 8.0 Hz), 4.95 (dd, 9.4, 2.1 Hz)	79.7, CH_2
**3''**	4.12 (dd, 6.7, 2.9 Hz)	53.4, CH	3.86 (t, 7.7 Hz)	41.5, CH	3.84 (td, 8.0, 2.4 Hz)	41.5, CH	4.02 (td, 8.0, 2.1 Hz)	41.7, CH
**4''**	5.51 (d, 2.9 Hz)	81.8, CH	4.95 (overlapped)	81.7, CH	4.91 (d, 7.8 Hz)	81.8, CH	5.04 (d, 8.0 Hz)	81.5, CH
**5''**	-	88.7, C	-	73.0, C	-	73.0, C	-	73.2, C
**6''**	1.23 (s)	23.5, CH_3	1.23 (s)	24.6, CH_3	1.20 (s)	24.7, CH_3	1.30 (s)	24.8, CH_3
**7''**	1.01 (s)	27.7, CH_3	1.22 (s)	27.3, CH_3	1.19 (s)	27.3, CH_3	1.23 (s)	27.7, CH_3
**8''**	-	171.5, C	-	171.8, C	-	171.8, C	-	167.4, C
**9''**	2.08 (s)	20.6, CH_3	1.88 (s)	21.0, CH_3	1.86 (s)	21.0, CH_3	6.31 (d, 16.0 Hz)	118.6, CH
**10''**	-	-	-	-	-	-	7.46 (m)	146.5, CH
**11''**	-	-	-	-	-	-	-	135.3, C
**12''**	-	-	-	-	-	-	7.39 (m)	131.8, CH
**13''**	-	-	-	-	-	-	7.39 (m)	129.3, CH
**14''**	-	-	-	-	-	-	7.39 (m)	130.1, CH
**15''**	-	-	-	-	-	-	7.39 (m)	129.3, CH
**16''**	-	-	-	-	-	-	7.39 (m)	131.8, CH

Isolated compounds details

(-)-(3*R*)-2′-*O*-methylmanuifolin H (**1**): HRESIMS *m/z* 341.1742 $[M+H]^+$ (calcd for $C_{21}H_{25}O_4$, 341.1753). $[\alpha]_D^{20}$ -70.9 (*c* 0.0127, MeOH). UV (MeOH) λ_{max} (log ε) 230 (3.55) nm, 287 (3.46) nm. For NMR data, see Table 3. For NMR spectra, see Figures S10 to S15.

(-)-(3*S*,4*R*)-bavaisoflavanol (**2**) : HRESIMS *m/z* 325.1431 $[M-H_2O+H]^+$ (calcd for $C_{20}H_{21}O_4$, 325.1440). $[\alpha]_D^{20}$ -154.6 (*c* 0.0087, MeOH). UV (MeOH) λ_{max} (log ε) 231 (3.52) nm, 287 (3.45) nm. For NMR data, see Table 3.

(-)-(3*R*)-manuifolin H (**3**): HRESIMS *m/z* 327.1594 $[M+H]^+$ (calcd for $C_{20}H_{23}O_4$, 327,1596). $[\alpha]_D^{20}$ -86.7 (*c* 0.0060, MeOH). For NMR data, see Table 3.

(-)-(3*S*)-7,4′-dihydroxy-2′-methoxyisoflavan (**4**): HRESIMS *m/z* 273.1116 $[M+H]^+$ (calcd for $C_{16}H_{17}O_4$, 273.1127). $[\alpha]_D^{20}$ -71.2 (*c* 0.0027, MeOH). NMR data: ^{1}H NMR (CD_3OD, 600 MHz) δ 2.75 (1H, dd, *J* = 15.6, 5.2 Hz, H_2-4eq), 2.90 (1H, dd, *J* = 15.6, 10.9 Hz, H_2-4ax), 3.42 (1H, overlapped, H-3), 3.80 (3H, s, 2'OCH_3), 3.91 (1H, t, *J* = 10.3 Hz, H_2-2ax), 4.17 (1H, dt, *J* = 10.3, 2.6 Hz, H_2-2eq), 6.22 (1H, d, *J* = 2.5 Hz, H-8), 6.31 (1H, dd, *J* = 8.1, 2.5 Hz, H-6), 6.34 (1H, dd, *J* = 8.3, 2.4 Hz, H-5'), 6.45 (1H, d, *J* = 2.4 Hz, H-3'), 6.86 (1H, d, *J* = 8.1 Hz, H-5), 6.93 (1H, d, *J* = 8.3 Hz, H-6'); ^{13}C NMR (CD_3OD, 151 MHz) δ 31.5 (CH_2-4), 2.9 (CH-3), 71.3 (CH_2-2), 100.0 (CH-3'), 103.9 (CH-8), 108.1 (CH-5'), 109.0 (CH-6), 114.9 (C-10), 121.7 (C-1'), 128.7 (CH-6'), 131.1 (CH-5), 156.3 (C-9), 157.6 (C-7), 158.7 (C-4'), 159.6 (C-2').

(-)-astraciceran (**5**): HRESIMS *m/z* 301.1068 $[M+H]^+$ (calcd for $C_{17}H_{17}O_5$, 301.1076). $[\alpha]_D^{20}$ -75 (*c* 0.0040, MeOH). NMR data: ^{1}H NMR (CD_3OD, 600 MHz) δ 2.78 (1H, ddd, *J* = 15.5, 5.3, 1.9 Hz, H_2-4b), 2.87 (1H, dd, *J* = 15.5, 10.4 Hz, H_2-4a), 3.49 (1H, tt, *J* = 10.4, 5.3, 3.5 Hz, H-3), 3.80 (3H, s, 2'OCH_3), 3.93 (1H, t, *J* = 10.4 Hz, H_2-2b), 4.16 (1H, ddd, *J* = 10.4, 3.5, 1.9 Hz, H_2-2a), 5.86 (1H, d, *J* = 1.2 Hz, H_2-7'b), 5.87 (1H, d, *J* = 1.2 Hz, H_2-7'a), 6.22 (1H, d, *J* = 2.5 Hz, H-8), 6.32 (1H, dd, *J* = 8.2, 2.5 Hz, H-6), 6.67 (1H, s, H-3'), 6.68 (1H, s, H-6'), 6.87 (1H, d, *J* = 8.2 Hz, H-5); ^{13}C NMR (CD_3OD, 151 MHz) δ 31.6 (CH_2-4), 33.2 (CH-3), 57.0 (2'OCH_3), 71.1 (CH_2-2), 95.9 (CH-3'), 102.3 (CH_2-7'), 103.8 (CH-8), 108.1 (CH-6'), 109.1 (CH-6), 114.6 (C-10), 123.1 (C-1'), 131.2 (CH-5), 142.7 (C-4'), 148.2 (C-5'), 153.8 (C-2'), 156.3 (C-9), 157.6 (C-7).

4-(2-(2-hydroxypropan-2-yl)-2,3,6,7-tetrahydro-5H-furo[3,2-g]chromen-6-yl)-3-methoxyphenol (**6**): HRESIMS *m/z* 357.1700 $[M+H]^+$ (calcd for $C_{21}H_{25}O_5$, 357.1702). $[\alpha]_D^{20}$ - 60.0 (*c* 0.0027, MeOH). UV (MeOH) λ_{max} (log ε) 211 (3.93) nm, 293 (3.60) nm. For NMR data, see Table 3. For NMR spectra, see Figures S16 to S20.

isobavachalcone (**7**): HRESIMS *m/z* 325.1438 $[M+H]^+$ (calcd for $C_{20}H_{21}O_4$, 325.1440). NMR data: ^{1}H NMR (CD3OD, 600 MHz) δ 1.66 (3H, s, H_3-5''), 1.78 (3H, s, H_3-4''), 3.32 (2H, overlapped,

H_2-1''), 5.23 (1H, t, *J* = 7.4 Hz, H-2''), 6.44 (1H, d, *J* = 8.7 Hz, H-5'), 6.85 (2H, d, *J* = 8.2 Hz, H-3, H-5), 7.63 (3H, m, H-2, H-6, H-8), 7.78 (1H, d, *J* = 15.2 Hz, H-7), 7.84 (1H, d, *J* = 8.7 Hz, H-6'); ^{13}C NMR (CD3OD, 151 MHz) δ 17.9 (CH_3-4''), 22.5 (CH_2-1''), 26.0 (CH_3-5''), 108.2 (CH-5'), 114.5 (C-1'), 116.6 (C-3'), 116.9 (CH-3, CH-5), 118.6 (CH-8), 123.6 (CH-2''), 127.9 (C-1), 130.4 (CH-6'), 131.8 (CH-2, CH-6), 131.9 (C-3''), 145.4 (CH-7), 161.5 (C-4), 163.7 (C-4'), 165.1 (C-2'), 193.8 (C-9).

pongamol (**8**): HRESIMS *m/z* 295.0964 $[M+H]^+$ (calcd for $C_{18}H_{15}O_4$, 295,0970). $[\alpha]_D^{20}$ -27.9 (*c* 0.0187, MeOH). NMR data: ^{1}H NMR ($CDCl_3$, 600 MHz) δ 4.15 (3H, s, OCH_3), 7.00 (1H, t, *J* = 2.2, 0.9 Hz, H-15), 7.17 (1H, s, H-8), 7.32 (1H, dd, *J* = 8.7, 0.9 Hz, H-12), 7.49 (2H, tt, *J* = 7.5, 1.2 Hz, H-3, H-5), 7.54 (1H, tt, *J* = 7.5, 1.2 Hz, H-4), 7.63 (1H, d, *J* = 2.2 Hz, H-14), 7.88 (1H, d, *J* = 8.7 Hz, H-11), 7.98 (2H, d, *J* = 7.5 Hz, H-2, H-6); ^{13}C NMR ($CDCl_3$, 151 MHz) δ 61.4 (OCH_3), 98.1 (CH-8), 105.4 (CH-15), 107.2 (CH-12), 119.8 (C-16), 122.4 (C-10), 126.6 (CH-11), 127.3 (CH-2, CH-6), 128.8 (CH-3, CH-5), 132.3 (CH-4), 135.8 (C-1), 145.0 (CH-14), 153.9 (C-17), 158.9 (C-13), 184.4 (C-7), 186.3 (C-9).

(-)-dehydroisoderricin (**9**): HRESIMS *m/z* 321.1488 $[M+H]^+$ (calcd for $C_{21}H_{21}O_3$, 321.1491). $[\alpha]_D^{20}$ -82.1 (*c* 0.0113, MeOH). NMR data: ^{1}H NMR (CD_3OD, 600 MHz) δ 1.90 (3H, d, *J* = 1.3 Hz, H_3-4''), 2.90 (1H, dd, *J* = 16.8, 3.0 Hz, H_2-3b), 3.10 (1H, dd, *J* = 16.8, 12.8 Hz, H_2-3a), 3.97 (3H, s, $7OCH_3$), 4.91 (1H, s, H_2-5''b), 5.00 (1H, t, *J* = 1.9 Hz, H_2-5''a), 5.61 (1H, dd, *J* = 12.8, 3.0 Hz, H-2), 6.80 (1H, d, *J* = 16.6 Hz, H-1''), 6.83 (1H, d, *J* = 8.9 Hz, H-6), 7.36 (1H, d, *J* = 16.6 Hz, H-2''), 7.39 (1H, m, H-4'), 7.44 (2H, t, *J* = 7.4 Hz, H-3', H-5'), 7.56 (2H, d, *J* = 7.4 Hz, H-2', H-6'), 7.82 (1H, d, *J* = 8.9 Hz, H-5); ^{13}C NMR (CD_3OD, 151 MHz) δ 18.2 (CH_3-4''), 44.9 (CH_2-3), 56.7 ($7OCH_3$), 81.1 (CH-2), 106.4 (CH-6), 115.5 (C-8), 116.5 (C-10), 117.4 (CH_2-5''), 119.4 (CH-1''), 127.2 (CH-2', CH-6'), 128.1 (CH-5), 129.6 (CH-4'), 129.8 (CH-3', CH-5'), 137.7 (CH-2''), 140.6 (C-1'), 144.6 (C-3''), 161.9 (C-9), 165.0 (C-7), 193.6 (C-4).

lanceolatin B (**10**): HRESIMS *m/z* 263.0701 $[M+H]^+$ (calcd for $C_{17}H_{11}O_3$, 263.0708). NMR data: ^{1}H NMR (CD_3OD, 600 MHz) δ 7.01 (1H, s, H-3), 7.44 (1H, d, *J* = 2.0 Hz, H-1''), 7.62 (3H, m, H-3', H-4', H-5'), 7.69 (1H, d, *J* = 8.8 Hz, H-6), 8.02 (1H, d, *J* = 2.0 Hz, H-2''), 8.11 (1H, d, *J* = 8.8 Hz, H-5), 8.14 (2H, m, H-2', H-6'); ^{13}C NMR (CD_3OD, 151 MHz) δ 105.2 (CH-1''), 108.1 (CH-3), 111.6 (CH-6), 118.7 (C-8), 120.0 (C-10), 122.2 (CH-5), 127.5 (CH-2', CH-6'), 130.4 (CH-3', CH-5'), 132.7 (C-1'), 133.1 (CH-4'), 148.2 (CH-2''), 152.4 (C-9), 160.1 (C-7), 165.3 (C-2), 180.5 (C-4).

(-)-purpurin (**11**): HRESIMS *m/z* 395.1465 $[M+H]^+$ (calcd for $C_{23}H_{23}O_6$, 395.1495). $[\alpha]_D^{20}$ -79.3 (*c* 0.0047, MeOH). For NMR data, see Table 4.

2-hydroxy-1-(2-(4-hydroxyphenyl)-4-oxo-3,4,8,9-tetrahydro-2H-furo[2,3-h]chromen-9-yl)-2-methylpropyl acetate (**12**): HRESIMS *m/z* 413.1586 $[M+H]^+$ (calcd for $C_{23}H_{25}O_7$, 413.1600). $[\alpha]_D^{20}$ -63.0 (*c* 0.0067, MeOH). UV (MeOH) λ_{max} (log ε) 225 (3.72) nm, 282 (3.65) nm. For NMR data, see Table 4. For NMR spectra, see Figures S21 to S25.

2-hydroxy-2-methyl-1-(4-oxo-2-phenyl-3,4,8,9-tetrahydro-2H-furo[2,3-h]chromen-9-yl) propyl acetate (**13**): HRESIMS *m/z* 397.1648 $[M+H]^+$ (calcd for $C_{23}H_{24}O_6$, 396.1573). $[\alpha]_D^{20}$ -63.3 (*c* 0.0060, MeOH). UV (MeOH) λ_{max} (log ε) 222 (3.74) nm, 239 (3.71) nm, 286 (3.63) nm. For NMR data, see Table 4. For NMR spectra, see Figures S26 to S30.

2-hydroxy-2-methyl-1-(4-oxo-2-phenyl-3,4,8,9-tetrahydro-2H-furo[2,3-h]chromen-9-yl)propyl cinnamate (**14**): HRESIMS *m/z* 485.1964 $[M+H]^+$ (calcd for $C_{30}H_{29}O_6$, 485.1964). $[\alpha]_D^{20}$ - 132.9 (*c* 0.0047, MeOH). UV (MeOH) λ_{max} (log ε) 223 (3.88) nm, 241 (3.85) nm, 279 (3.82) nm. For NMR data, see Table 4. For NMR spectra, see Figures S31 to S36.

(-)-(6a*R*,11a*R*)-medicarpin (**15)**: HRESIMS *m/z* 271.0975 $[M+H]^+$ (calcd for $C_{16}H_{15}O_4$, 271.0970). $[\alpha]_D^{20}$ -240.0 (*c* 0.0020, MeOH). NMR data: ^{1}H NMR (CD_3OD, 600 MHz) δ 3.54 (2H, m, H-6'', H-6a), 3.74 (3H, s, 9OCH_3), 4.22 (1H, d, *J* = 5.8 Hz, H-6'), 5.48 (1H, d, *J* = 6.4 Hz, H-11a), 6.31 (1H, d, *J* = 2.4 Hz, H-4), 6.38 (1H, d, *J* = 2.3 Hz, H-10), 6.45 (1H, dd, *J* = 8.1, 2.3 Hz, H-8), 6.49 (1H, dd, *J* = 8.4, 2.4 Hz, H-2), 7.17 (1H, d, *J* = 8.1 Hz, H-7), 7.29 (1H, d, *J* = 8.4 Hz, H-1); ^{13}C NMR (CD_3OD, 151 MHz) δ 40.9 (CH-6a), 55.9 (9OCH_3), 67.6 (CH_2-6), 80.1 (CH-11a), 97.6 (CH-10), 104.1 (CH-4), 107.2 (CH-8), 110.7 (CH-2), 112.9 (C-11b), 120.9 (C-6b), 126.0 (CH-7), 133.2 (CH-1), 158.1 (C-4a), 160.1 (C-3), 162.1 (C-10a), 162.6 (C-9).

(-)-(6a*R*,11a*R*)-maackiain (**16)**: HRESIMS *m/z* 285.0753 $[M+H]^+$ (calcd for $C_{16}H_{13}O_5$, 285.0763). $[\alpha]_D^{20}$ -103.1 (*c* 0.0053, MeOH). NMR data: ^{1}H NMR (CD_3OD, 600 MHz) δ 3.48 (1H, ddd, *J* = 10.9, 6.9, 4.8 Hz, H-6a), 3.56 (1H, t, *J* = 10.9 Hz, H-6''), 4.22 (1H, dd, *J* = 10.9, 4.8 Hz, H-6'), 5.45 (1H, d, *J* = 6.9 Hz, H-11a), 5.85 (1H, d, *J* = 1.1 Hz, OCH_2O''), 5.88 (1H, d, *J* = 1.1 Hz, OCH_2O'), 6.30 (1H, d, *J* = 2.4 Hz, H-4), 6.37 (1H, s, H-10), 6.48 (1H, dd, *J* = 8.3, 2.4 Hz, H-2), 6.81 (1H, s, H-7), 7.26 (1H, d, *J* = 8.3 Hz, H-1); ^{13}C NMR (CD_3OD, 151 MHz) δ 41.6 (CH-6a), 67.4 (CH_2-6), 80.1 (CH-11a), 94.2 (CH-10), 102.5 (OCH_2O), 104.1 (CH-4), 106.0 (CH-7), 110.7 (CH-2), 112.9 (C-11b), 119.8 (C-6b), 133.1 (CH-1), 143.1 (C-8), 149.5 (C-9), 155.6 (C-10a), 158.0 (C-4a), 160.1 (C-3).

(-)-(6a*R*,11a*R*,2'*R*)-emoroidocarpan (**17**): HRESIMS m/z 351.1214 $[M+H]^+$ (calcd for $C_{21}H_{19}O_5$, 351.1233). $[\alpha]_D^{20}$ -273.0 (c 0.0100, MeOH). NMR data: ^{1}H NMR (CD_3OD, 600 MHz) δ 1.75 (3H, t, J = 1.2 Hz, H_3-4'), 2.99 (1H, ddd, J = 15.3, 7.7, 1.3 Hz, H-1'b), 3.33 (1H, dd, J = 15.3, 9.2 Hz, H-1'a), 3.49 (1H, ddd, J = 10.7, 7.0, 4.8 Hz, H-6a), 3.57 (1H, t, J = 10.7 Hz, H-6''), 4.22 (1H, dd, J = 10.7, 4.8 Hz, H-6'), 4.88 (1H, p, J = 1.2 Hz, H-5'b), 5.05 (1H, p, J = 1.2 Hz, H-5'a), 5.20 (1H, t, J = 9.2, 7.1 Hz, H-2'), 5.47 (1H, d, J = 7.0 Hz, H-11a), 5.86 (1H, d, J = 1.2 Hz, OCH_2O''), 5.88 (1H, d, J = 1.2 Hz, OCH_2O'), 6.27 (1H, s, H-4), 6.37 (1H, s, H-10), 6.81 (1H, s, H-7), 7.23 (1H, s, H-1); ^{13}C NMR (CD_3OD, 151 MHz) δ 17.2 (CH_3-4'), 34.9 (CH_2-1'), 41.6 (CH-6a), 67.6 (CH_2-6), 80.4 (CH-11a), 87.9 (CH-2'), 94.2 (CH-10), 98.6 (CH-4), 102.5 (OCH_2O), 106.0 (CH-7), 112.1 (CH_2-5'), 113.7 (C-11b), 119.8 (C-6b), 122.0 (C-2), 127.8 (CH-1), 143.1 (C-8), 145.8 (C-3'), 149.5 (C-9), 155.5 (C-10a), 157.5 (C-4a), 162.5 (C-3).

(-)-(6a*R*,11a*R*,2'*R*)-4'-hydroxyemoroidocarpan (**18**): HRESIMS *m/z* 367.1190 $[M+H]^+$ (calcd for $C_{21}H_{19}O_6$, 367.1182). $[\alpha]_D^{20}$ -222.0 (*c* 0.0033, MeOH). NMR data: ^{1}H NMR (CD_3OD, 600 MHz) δ 3.07 (1H, dd, *J* = 15.3, 7.9 Hz, H-1'b), 3.37 (1H, dd, *J* = 15.3, 9.6 Hz, H-1'a), 3.49 (1H, ddd, *J* = 10.7, 7.0, 4.8 Hz, H-6a), 3.57 (1H, t, *J* = 10.8 Hz, H-6''), 4.15 (2H, s, H_2-4'), 4.22 (1H, dd, *J* = 10.8, 4.8 Hz, H-6'), 5.21 (2H, s, H-5'), 5.31 (1H, t, *J* = 9.6, 7.9 Hz, H-2'), 5.47 (1H, d, *J* = 7.0 Hz, H-11a), 5.86 (1H, s, OCH_2O''), 5.88 (1H, s, OCH_2O'), 6.29 (1H, s, H-4), 6.37 (1H, s, H-10), 6.81 (1H, s, H-7), 7.24 (1H, s, H-1); ^{13}C NMR (MeOD, 151 MHz) δ 35.3 (CH_2-1'), 41.5 (CH-6a), 62.6 (CH_2-4'), 67.6 (CH_2-6), 80.3 (CH-11a), 85.4 (CH-2'), 94.2 (CH-10), 98.7 (CH-4), 102.5 (OCH_2O), 106.0 (CH-7), 110.8 (CH_2-5'), 113.8 (C-11b), 119.8 (C-6b), 121.9 (C-2), 127.9 (CH-1), 143.1 (C-8), 149.5 (C-9), 149.7 (C-3'), 155.5 (C-10a), 157.5 (C-4a), 162.2 (C-3).

(-)-(2'*R*)-tephcalostan (**19**): HRESIMS *m/z* 363.0883 $[M+H]^+$ (calcd for $C_{21}H_{15}O_6$, 363.0869). $[\alpha]_D^{20}$ -30.0 (*c* 0.0047, MeOH). NMR data: ^{1}H NMR (CD_3OD, 600 MHz) δ 1.79 (3H, t, *J* = 1.2 Hz, H_3-4'), 3.18 (1H, ddd, *J* = 15.9, 7.9, 1.3 Hz, H-1'b), 3.54 (1H, ddd, *J* = 15.9, 9.5, 0.7 Hz, H-1'a), 4.96 (1H, p, *J* = 1.2 Hz, H-5'b), 5.13 (1H, p, *J* = 1.2 Hz, H-5'a), 5.40 (1H, t, *J* = 9.5, 7.9 Hz, H-2'), 6.08 (2H, s, OCH_2O), 6.92 (1H, s, H-4), 7.28 (1H, s, H-10), 7.35 (1H, s, H-7), 7.84 (1H, t, *J* = 1.3, 0.7 Hz, H-1); ^{13}C NMR (CD_3OD, 151 MHz) δ 16.9 (CH_3-4'), 34.3 (CH_2-4'), 88.6 (CH-2'), 94.8 (CH-10), 98.6 (CH,4), 100.1 (CH-7), 103.1 (OCH_2O), 106.8 (C-11b), 112.5 (CH_2-5'), 117.8 (C-6b), 118.0 (CH-1), 127.0 (C-2), 144.7 (C-3'), 147.4 (C-8), 148.8 (C-9), 151.7 (C-10a), 155.7 (C-4a), 161.7 (C-11a), 164.5 (C-3).

glabrone (**20**): HRESIMS *m/z* 337.1070 $[M+H]^+$ (calcd for $C_{20}H_{17}O_5$, 337.1076). For NMR data, see Table 2.

6-hydroxy-glabrone (**21**): HRESIMS *m/z* 353.1020 $[M+H]^+$ (calcd for $C_{20}H_{17}O_6$, 353.1025). UV (MeOH) λ_{max} (log ε) 226 (4.43) nm, 258 (4.02) nm, 330 (3.80) nm. For NMR data, see Table 2. For NMR spectra, see Figures S37 to S41.

formononetin (**22**): HRESIMS *m/z* 269.0802 $[M+H]^+$ (calcd for $C_{16}H_{13}O_4$, 269.0814). NMR data: ^{1}H NMR (CD_3OD, 600 MHz) δ 3.83 (3H, s, 4'OCH_3), 6.86 (1H, d, *J* = 2.3 Hz, H-8), 6.94 (1H, dd, *J* = 8.8, 2.3 Hz, H-6), 6.99 (3H, d, *J* = 8.8 Hz, H-3', H-5'), 7.47 (2H, d, *J* = 8.8 Hz, H-2', H-6'), 8.06 (1H, d, *J* = 8.8 Hz, H-5), 8.16 (1H, s, H-2); ^{13}C NMR (CD_3OD, 151 MHz) δ 55.7 (4'OCH_3), 103.3 (CH-8), 114.8 (CH-3', CH-5'), 116.6 (CH-6), 118.1 (C-10), 125.6 (C-1'), 125.7 (C-3), 128.5 (CH-5), 131.4 (CH-2', CH-6'), 154.8 (CH-2), 160.0 (C-9), 161.3 (C-4'), 165.0 (C-7), 178.1 (C-4).

munetone (**23**): HRESIMS *m/z* 417.1962 $[M+H]^+$ (calcd for $C_{26}H_{24}O_5$, 417.1702). NMR data: ^{1}H NMR (CD_3OD, 600 MHz) δ 1.43 (6H, s, H_3-5''', H_3-6'''), 1.49 (6H, s, H_3-5'', H_3-6''), 3.58 (3H, s, 2'OCH_3), 5.76 (1H, d, *J* = 10.0 Hz, H-3'''), 5.90 (1H, d, *J* = 10.0 Hz, H-3''), 6.54 (1H, d, *J* = 10.0 Hz, H-4''), 6.61 (1H, d, *J* = 8.3 Hz, H-5'), 6.63 (1H, d, *J* = 10.0 Hz, H-4'''), 6.88 (1H, s, H-8), 7.06 (1H, d, *J* = 8.3 Hz, H-6'), 7.81 (1H, s, H-5), 8.10 (1H, s, H-2); ^{13}C NMR (CD_3OD, 151 MHz) δ 28.0 (CH_3-5''',

CH_3-6'''), 28.7 (CH_3-5'', CH_3-6''), 62.3 (2'O CH_3), 77.1 (C-2'''), 79.4 (C-2''), 105.0 (CH-8), 113.3 (CH-5'), 116.3 (C-3'), 118.1 (CH-4'''), 118.6 (C-1'), 119.3 (C-10), 121.7 (C-6), 121.9 (CH-4''), 123.3 (C-3), 124.0 (CH-5), 131.8 (CH-3'''), 132.8 (CH-6'), 133.6 (CH-3''), 155.8 (C-4'), 156.1 (C-2'), 156.2 (CH-2), 159.3 (C-9), 159.9 (C-7), 178.1 (C-4).

acicerone (**24**): HRESIMS *m/z* 313.0708 $[M+H]^+$ (calcd for $C_{17}H_{13}O_6$, 313.0712). NMR data: ^1H NMR (CD_3OD, 600 MHz) δ 4.01 (3H, d, *J* = 2.6 Hz, $7OCH_3$), 5.98 (2H, d, *J* = 2.6 Hz, OCH_2O), 6.88 (1H, d, *J* = 8.0 Hz, H-5'), 6.99 (1H, d, *J* = 8.0 Hz, H-6'), 7.07 (1H, s, H-2'), 7.13 (1H, s, H-8), 7.48 (1H, s, H-5), 8.20 (1H, s, H-2); ^{13}C NMR (CD_3OD, 151 MHz) δ 57.0 ($7OCH_3$), 100.7 (CH-8), 102.5 (OCH_2O), 108.9 (CH-5), 109.1 (CH-5'), 110.8 (CH-2'), 119.0 (C-10), 123.8 (CH-6'), 125.3 (C-3), 127.3 (C-1'), 146.8 (C-6), 149.1 (C-3', C-4'), 153.6 (C-9), 154.9 (C-2), 155.6 (C-7), 177.8 (C-4).

alfalone (**25**): HRESIMS *m/z* 299.0921 $[M+H]^+$ (calcd for $C_{17}H_{15}O_5$, 299.0920). NMR data: ^1H NMR (CD_3OD, 600 MHz) δ 3.83 (3H, s, $4'OCH_3$), 4.01 (3H, s, $7OCH_3$), 6.99 (2H, d, *J* = 8.4 Hz, H-3', H-5'), 7.13 (1H, s, H-8), 7.47 (2H, d, *J* = 8.4 Hz, H-2', H-6'), 7.49 (1H, s, H-5), 8.20 (1H, s, H-2); ^{13}C NMR (CD_3OD, 151 MHz) δ 55.7 ($4'OCH_3$), 57.0 ($7OCH_3$), 100.8 (CH-8), 108.9 (CH-5), 114.9 (CH-3', CH-5'), 119.1 (C-10), 125.3 (C-3), 125.7 (C-1'), 131.4 (CH-2', CH-6'), 146.7 (C-6), 153.7 (C-9), 154.7 (CH-2), 155.6 (C-7), 161.2 (C-4'), 178.0 (C-4).

afrormosin (**26**): HRESIMS *m/z* 299.0921 $[M+H]^+$ (calcd for $C_{17}H_{15}O_5$, 299.0920). NMR data: ^1H NMR (CD_3OD, 600 MHz) δ 3.83 (3H, s, $4'OCH_3$), 3.97 (3H, s, $6OCH_3$), 6.95 (1H, s, H-8), 6.99 (2H, d, *J* = 8.7 Hz, H-3', H-5'), 7.48 (2H, d, *J* = 8.7 Hz, H-2', H-6'), 7.58 (1H, s, H-5), 8.18 (1H, s, H-2); ^{13}C NMR (CD_3OD, 151 MHz) δ 55.7 ($4'OCH_3$), 56.6 ($7OCH_3$), 103.9 (CH-8), 105.4 (CH-5), 114.8 (CH-3', CH-5'), 117.9 (C-10), 125.7 (C-1'), 131.4 (CH-2', CH-6'), 148.6 (C-6), 154.5 (CH-2), 161.2 (C-4'), 177.8 (C-4).

wighteone (**27**): HRESIMS *m/z* 339.1218 $[M+H]^+$ (calcd for $C_{20}H_{19}O_5$, 339.1233). NMR data: ^1H NMR (CD_3OD, 600 MHz) δ 1.66 (3H, s, H_3-4''), 1.78 (3H, s, H_3-5''), 3.32 (2H, overlapped, H_2-1''), 5.23 (1H, t, *J* = 7.6 Hz, H-2''), 6.39 (1H, s, H-8), 6.85 (2H, d, *J* = 7.7 Hz, H-3', H-5'), 7.37 (2H, d, *J* = 7.7 Hz, H-2', H-6'), 8.04 (1H, s, H-2); ^{13}C NMR (CD_3OD, 151 MHz) δ 17.9 (CH_3-5''), 22.3 (CH_2-1''), 26.0 (CH_3-4''), 93.9 (CH-8), 106.1 (C-10), 113.1 (C-6), 116.3 (CH-3', CH-5'), 123.2 (C-1'), 123.3 (CH-2''), 124.5 (C-3), 131.4 (CH-2', CH-6'), 132.2 (C-3''), 154.6 (CH-2), 157.6 (C-9), 158.8 (C-4'), 160.5 (C-5), 163.7 (C-7), 182.4 (C-4).

dehydroneotenone (**28**): HRESIMS *m/z* 337.0712 $[M+H]^+$ (calcd for $C_{19}H_{13}O_6$, 337.0712). NMR data: ^1H NMR (CD_3OD, 600 MHz) δ 3.73 (3H, s, $2'OCH_3$), 5.96 (2H, s, OCH_2O), 6.76 (1H, s, H-3'), 6.80 (1H, s, H-6'), 7.07 (1H, d, *J* = 2.3 Hz, H-3''), 7.77 (1H, s, H-8), 7.96 (1H, d, *J* = 2.3 Hz, H-2''), 8.19 (1H, s, H-2), 8.48 (1H, s, H-5); ^{13}C NMR (CD_3OD, 151 MHz) δ 57.1 ($2'OCH_3$), 96.3 (CH-3'), 101.0 (CH-8), 102.8 (OCH_2O), 108.0 (CH-3''), 112.0 (CH-6'), 114.0 (C-1'), 119.4 (CH-5), 121.9

(C-10), 122.8 (C-3), 127.9 (C-6), 142.6 (C-5'), 149.7 (CH-2''), 150.1 (C-4'), 154.6 (C-2'), 155.8 (C-9), 157.3 (CH-2), 159.0 (C-7), 178.9 (C-4).

(-)-12a-hydroxypachyrrhizone (**29**): HRESIMS *m/z* 365.0652 $[M-H_20+H]^+$ (calcd for $C_{20}H_{13}O_7$, 365.0661). $[\alpha]_D^{20}$ -46.1 (*c* 0.0093, MeOH). NMR data: ^1H NMR (CD_3OD, 600 MHz) δ 4.04 (3H, s, $8OCH_3$), 4.50 (1H, d, *J* = 11.6 Hz, H-6''), 4.63 (1H, d, *J* = 11.6 Hz, H-6'), 4.65 (1H, s, H6a), 5.79 (1H, s, OCH_2O''), 5.85 (1H, s, OCH_2O'), 6.44 (1H, s, H-4), 6.55 (1H, s, H-1), 6.86 (1H, s, H-3'), 7.74 (1H, s, H-2'), 7.89 (1H, s, H-11); ^{13}C NMR (CD_3OD, 151 MHz) δ 61.5 ($8OCH_3$), 65.0 (CH_2-6), 69.9 (C-12a), 77.6 (CH-6a), 99.6 (CH-4), 102.7 (OCH_2O), 107.3 (CH-1), 108.3 (CH-3'), 109.9 (C-12b), 115.0 (CH-11), 118.2 (C-11a), 125.7 (C-10), 134.6 (C-8), 143.4 (C-2), 148.0 (CH-2'), 150.6 (C-7a), 150.9 (C-3), 151.3 (C-4a), 152.6 (C-9), 194.5 (C-12).

(-)-12a-hydroxydolineone (**30**): HRESIMS *m/z* 335.0558 $[M-H_20+H]^+$ (calcd for $C_{19}H_{11}O_6$, 335.0556). $[\alpha]_D^{20}$ -41.3 (*c* 0.0053, MeOH). NMR data: ^1H NMR (CD_3OD, 600 MHz) δ 4.48 (1H, dd, *J* = 12.2, 1.1 Hz, H-6''), 4.57 (1H, dd, *J* = 12.2, 2.4 Hz, H-6'), 4.64 (1H, dd, *J* = 2.4, 1.1 Hz, H-6a), 5.79 (1H, d, *J* = 1.1 Hz, OCH_2O''), 5.85 (1H, d, *J* = 1.1 Hz, OCH_2O'), 6.43 (1H, s, H-4), 6.56 (1H, s, H-1), 6.86 (1H, d, *J* = 2.3 Hz, H-3'), 7.02 (1H, s, H-8), 7.71 (1H, d, *J* = 2.3 Hz, H-2'), 8.18 (1H, s, H-11); ^{13}C NMR (CD_3OD, 151 MHz) δ 65.0 (CH_2-6), 69.8 (C-12a), 77.5 (CH-6a), 99.7 (CH-4), 100.4 (CH-8), 102.7 (OCH_2O), 107.4 (CH-1), 107.9 (CH-3'), 110.0 (C-12b), 117.0 (C-11a), 121.8 (CH-11), 124.6 (C-10), 143.2 (C-2), 148.0 (CH-2'), 150.9 (C-3), 151.3 (C-4a), 159.6 (C-7a), 161.4 (C-9), 194.5 (C-12).

(+)-12a-hydroxyerosone (**31**): HRESIMS *m/z* 351.0867 $[M-H_20+H]^+$ (calcd for $C_{20}H_{15}O_6$, 351.0869). $[\alpha]_D^{20}$ 97.5 (*c* 0.0067, MeOH). NMR data: ^1H NMR (CD_3OD, 600 MHz) δ 3.66 (3H, s, $2OCH_3$), 3.77 (3H, s, $3OCH_3$), 4.50 (1H, dd, *J* = 12.1, 3.4 Hz, H-6''), 4.59 (1H, dd, *J* = 12.1, 1.6 Hz, H-6'), 4.65 (1H, s, H-6a), 6.54 (1H, s, H-4), 6.70 (1H, s, H-1), 6.85 (1H, s, H-3'), 7.02 (1H, s, H-8), 7.71 (1H, s, H-2'), 8.19 (1H, s, H-11); ^{13}C NMR (CD_3OD, 151 MHz) δ 56.3 ($3OCH_3$), 57.1 ($2OCH_3$), 65.0 (CH_2-6), 69.7 (C-12a), 77.8 (CH-6a), 100.4 (CH-8), 102.3 (CH-4), 107.9 (CH-3'), 109.3 (C-12b), 112.6 (CH-1), 117.0 (C-11a), 121.7 (CH-11), 124.6 (C-10), 144.9 (C-2), 148.1 (CH-2'), 150.6 (C-4a), 152.9 (C-3), 159.5 (C-7a), 161.4 (C-9), 194.5 (C-12).

(+)-erosone (**32**): HRESIMS *m/z* 353.1016 $[M+H]^+$ (calcd for $C_{20}H_{17}O_6$, 353.1025). $[\alpha]_D^{20}$ 46.7 (*c* 0.0060, MeOH). NMR data: ^1H NMR (CD_3OD, 600 MHz) δ 3.67 (3H, s, $2OCH_3$), 3.76 (3H, s, $3OCH_3$), 3.96 (1H, d, *J* = 3.8 Hz, H-12a), 4.23 (1H, d, *J* = 12.2 Hz, H-6''), 4.59 (1H, dd, *J* = 12.2, 3.0 Hz, H-6'), 5.03 (1H, t, *J* = 3.8, 3.0 Hz, H-6a), 6.50 (1H, s, H-4), 6.73 (1H, s, H-1), 6.85 (1H, d, *J* = 2.4 Hz, H-3'), 7.07 (1H, s, H-8), 7.71 (1H, d, *J* = 2.4 Hz, H-2'), 8.20 (1H, s, H-11); ^{13}C NMR (CD_3OD, 151 MHz) δ 46.0 (CH-12a), 56.4 ($3OCH_3$), 57.2 ($2OCH_3$), 67.5 (CH_2-6), 73.8 (CH-6a), 100.4 (CH-8), 102.5 (CH-4), 106.2 (C-12b), 107.9 (CH-3'), 112.5 (CH-1), 117.4 (C-11a), 121.7 (CH-11), 124.5 (C-10), 145.0 (C-2), 148.0 (CH-2'), 149.7 (C-4a), 151.3 (C-3), 160.8 (C-7a), 161.3 (C-9), 192.9 (C-12).

12-deoxo-12α-acetoxy-12aβ-hydroxyelliptone (**33**): HRESIMS *m/z* 353.1012 $[M-C_2H_4O_2+H]^+$ (calcd for $C_{20}H_{17}O_6$, 353.1025). $[\alpha]_D^{20}$ - 80.6 (*c* 0.0053, MeOH). UV (MeOH) λ_{max} (log ε) 225 (3.82) nm, 326 (3.61) nm. For NMR data, see Table 1. For NMR spectra, see Figures S42 to S46.

(-)-12-deoxo-12α-acetoxyelliptone (**34**): HRESIMS *m/z* 337.1065 $[M-C_2H_4O_2+H]^+$ (calcd for $C_{20}H_{17}O_5$, 337.1076). $[\alpha]_D^{20}$ -293.0 (*c* 0.0187, MeOH). NMR data: ^{1}H NMR (CD_3OD, 600 MHz) δ 1.74 (3H, s, Ac), 3.75 (1H, tt, *J* = 6.2, 4.6, 1.4 Hz, H-12a), 3.78 (3H, s, $2OCH_3$), 3.79 (3H, s, $3OCH_3$), 4.30 (1H, ddd, *J* = 10.1, 5.1, 1.4 Hz, H-6), 4.50 (1H, t, *J* = 11.2, 10.1 Hz, H-6), 5.01 (1H, dtd, *J* = 11.2, 6.2, 5.1, 1.1 Hz, H-6a), 6.47 (1H, s, H-4), 6.47 (1H, d, *J* = 4.6 Hz, H-12), 6.89 (1H, dd, *J* = 2.2, 0.9 Hz, H-3'), 6.89 (1H, s, H-1), 7.10 (1H, dd, *J* = 8.4, 0.9 Hz, H-10), 7.17 (1H, d, *J* = 8.4 Hz, H-11), 7.68 (1H, d, *J* = 2.2 Hz, H-2'); ^{13}C NMR (CD_3OD, 151 MHz) δ 20.7 (Ac), 37.6 (CH-12a), 56.4 ($3OCH_3$), 57.2 ($2OCH_3$), 65.4 (CH_2-6), 68.5 (CH-12), 70.6 (CH-6a), 101.5 (CH-4), 104.8 (CH-3'), 105.8 (CH-10), 110.5 (C-12b), 112.9 (C-11a), 114.6 (CH-1), 118.2 (C-8), 127.7 (CH-11), 144.8 (C-2), 145.8 (CH-2'), 148.2 (7C-a), 150.4 (C-4a), 151.1 (C-3), 158.2 (C-9), 171.8 (CO).

(-)-(6a*R*,12a*R*)-elliptone (**35**): HRESIMS *m/z* 353.1015 $[M+H]^+$ (calcd for $C_{20}H_{17}O_6$, 353.1025). $[\alpha]_D^{20}$ -31.4 (*c* 0.0073, MeOH). NMR data: ^{1}H NMR (CD_3OD, 600 MHz) δ 3.67 (3H, s, $2OCH_3$), 3.75 (3H, s, $3OCH_3$), 3.98 (1H, d, *J* = 4.1 Hz, H-12a), 4.28 (1H, dt, *J* = 12.3, 1.0 Hz, H-6''), 4.68 (1H, dd, *J* = 12.3, 3.1 Hz, H-6'), 5.17 (1H, td, *J* = 4.1, 3.1, 1.0 Hz, H-6a), 6.51 (1H, s, H-4), 6.72 (1H, s, H-1), 6.98 (1H, dd, *J* = 2.2, 0.9 Hz, H-3'), 7.19 (1H, dd, *J* = 8.8, 0.9 Hz, H-10), 7.75 (1H, d, *J* = 2.2 Hz, H-2'), 7.86 (1H, d, *J* = 8.8 Hz, H-11); ^{13}C NMR (CD_3OD, 151 MHz) δ 45.8 (CH-12a), 56.4 ($3OCH_3$), 57.2 ($2OCH_3$), 67.3 (CH_2-6), 74.4 (CH-6a), 102.6 (CH-4), 105.5 (CH-3'), 106.3 (C-12b), 107.4 (CH-10), 112.5 (CH-1), 114.7 (C-11a), 118.4 (C-8), 124.8 (CH-11), 145.1 (C-2), 146.9 (CH-2'), 149.6 (C-4a), 151.3 (C-3), 157.5 (C-7a), 161.8 (C-9), 192.1 (C-12).

(+)-(6a*S*,12a*S*)-12a-hydroxyelliptone (**36**): HRESIMS *m/z* 351.0856 $[M-H_20+H]^+$ (calcd for $C_{20}H_{15}O_6$, 351.0869). $[\alpha]_D^{20}$ 23.3 (*c* 0.0060, MeOH). NMR data: ^{1}H NMR (CD_3OD, 600 MHz) δ 3.66 (3H, s, $2OCH_3$), 3.76 (3H, s, $3OCH_3$), 4.54 (1H, dd, *J* = 12.3, 1.1 Hz, H-6''), 4.68 (1H, dd, *J* = 12.3, 2.5 Hz, H-6'), 4.78 (1H, dd, *J* = 2.5, 1.1 Hz, H-6a), 6.54 (1H, s, H-4), 6.70 (1H, s, H-1), 6.95 (1H, dd, *J* = 2.2, 0.9 Hz, H-3'), 7.21 (1H, dd, *J* = 8.8, 0.9 Hz, H-10), 7.75 (1H, d, *J* = 2.2 Hz, H-2'), 7.85 (1H, d, *J* = 8.8 Hz, H-11); ^{13}C NMR (CD_3OD, 151 MHz) δ 56.3 ($3OCH_3$), 57.1 ($2OCH_3$), 64.9 (CH_2-6), 69.3 (C-12a), 78.3 (CH-6a), 102.3 (CH-4), 105.4 (CH-3'), 107.6 (CH-10), 109.5 (C-12b), 112.5 (CH-1), 114.3 (C-11a), 118.3 (C-8), 124.7 (C-H11), 145.0 (C-2), 147.0 (CH-2'), 150.5 (C-4a), 152.9 (C-3), 156.6 (C-7a), 161.8 (C-9), 193.9 (C-12).

(-)-(6a*S*,11a*S*,2'*R*)-rotenone (**37**): HRESIMS *m/z* 395.1488 $[M+H]^+$ (calcd for $C_{23}H_{23}O_6$, 395.1495). $[\alpha]_D^{20}$ -223.3 (*c* 0.0060, MeOH). NMR data: ^{1}H NMR (CD_3OD, 600 MHz) δ 1.76 (3H, t, *J* = 1.2 Hz, H_3-6'), 2.95 (1H, dd, *J* = 15.8, 8.0 Hz, H-3'b), 3.33 (1H, overlapped, H-3'a), 3.67 (3H, s, $2OCH_3$), 3.77 (3H, s, $3OCH_3$), 3.87 (1H, d, *J* = 4.0 Hz, H-12a), 4.22 (1H, dt, *J* = 12.3, 1.0 Hz, H-6), 4.58 (1H, dd, *J* = 12.3, 3.0 Hz, H-6), 4.92 (1H, p, *J* = 1.2 Hz, H-5'b), 5.01 (1H, td, *J* = 4.0, 3.0, 1.0 Hz, H-6a),

5.06 (1H, p, *J* = 1.2 Hz, H-5'a), 5.31 (1H, t, *J* = 9.7, 8.0 Hz, H-2'), 6.49 (1H, s, H-4), 6.51 (1H, d, *J* = 8.6 Hz, H-10), 6.71 (1H, s, H-1), 7.80 (1H, d, *J* = 8.6 Hz, H-11); ^{13}C NMR (CD_3OD, 151 MHz) δ 16.7 (CH_3-6'), 31.7 (CH_2-3'), 45.4 (CH-12a), 56.1 (3OCH_3), 56.8 (2OCH_3), 67.0 (CH_2-6), 73.3 (CH-6a), 89.0 (CH-2'), 102.2 (CH-4), 105.3 (CH-10), 106.4 (C-13a), 112.2 (CH-1), 112.4 (CH_2-5'), 114.2 (C-8), 130.5 (CH-11), 144.8 (C-2, C-4'), 149.3 (C-4a), 151.1 (C-3), 159.4 (C-7a), 168.6 (C-9), 191.3 (C-12).

(-)-(6a*R*,12a*R*)-tephrosin (**38**): HRESIMS *m/z* 393.1320 $[M-H_20+H]^+$ (calcd for $C_{23}H_{21}O_6$, 393.1338). $[\alpha]_D^{20}$ -47.9 (*c* 0.0140, MeOH). NMR data: ^{1}H NMR (CD_3OD, 600 MHz) δ 1.36 (3H, s, H_3-6'), 1.43 (3H, s, H_3-5'), 3.67 (3H, s, 2OCH_3), 3.78 (3H, s, 3OCH_3), 4.48 (1H, dd, *J* = 12.7, 1.6 Hz, H-6''), 4.60 (1H, t, *J* = 2.4, 1.6 Hz, H-6a), 4.60 (1H, dd, *J* = 12.7, 2.4 Hz, H-6'), 5.66 (1H, d, *J* = 10.1 Hz, H-3'), 6.45 (1H, d, *J* = 8.8 Hz, H-10), 6.54 (1H, s, H-4), 6.59 (1H, d, *J* = 10.1 Hz, H-4'), 6.70 (1H, s, H-1), 7.69 (1H, d, *J* = 8.8 Hz, H-11); ^{13}C NMR (CD_3OD, 151 MHz) δ 28.2 (CH_3-6'), 28.6 (CH_3-5'), 56.3 (3OCH_3), 57.1 (2OCH_3), 64.9 (CH_2-6), 69.0 (12a), 77.9 (CH-6a), 79.0 (C-2'), 102.3 (CH-4), 109.6 (C-12b), 110.3 (C-8), 112.5 (CH-10), 112.6 (CH-1), 113.4 (C-11a), 116.2 (CH-4'), 129.3 (CH-11), 130.4 (CH-3'), 145.0 (C-2), 150.5 (C-4a), 152.9 (C-3), 157.6 (C-7a), 161.6 (C-9), 193.1 (C-12).

(-)-(6a*S*,12a*S*)-deguelin (**39**): HRESIMS *m/z* 395.1481 $[M+H]^+$ (calcd for $C_{23}H_{23}O_6$, 395.1495). $[\alpha]_D^{20}$ -79.5 (*c* 0.0267, MeOH). NMR data: ^{1}H NMR (CD_3OD, 600 MHz) δ 1.35 (3H, s, H_3-6'), 1.42 (3H, s, H_3-5'), 3.68 (3H, s, 2OCH_3), 3.75 (3H, s, 3OCH_3), 3.86 (1H, d, *J* = 4.0 Hz, H-12a), 4.21 (1H, dt, *J* = 12.2, 1.0 Hz, H-6), 4.60 (1H, dd, *J* = 12.2, 3.0 Hz, H-6), 4.98 (1H, td, *J* = 4.0, 3.0, 1.0 Hz, H-6a), 5.65 (1H, d, *J* = 10.1 Hz, H-3'), 6.43 (1H, dd, *J* = 8.7, 0.7 Hz, H-10), 6.49 (1H, s, H-4), 6.63 (1H, dd, *J* = 10.1, 0.7 Hz, H-4'), 6.72 (1H, d, *J* = 1.0 Hz, H-1), 7.69 (1H, d, *J* = 8.7 Hz, H-11); ^{13}C NMR (CD_3OD, 151 MHz) δ 28.2 (CH_3-6'), 28.6 (CH_3-5'), 45.4 (CH-12a), 56.4 (3OCH_3), 57.2 (2OCH_3), 67.3 (CH_2-6), 73.9 (CH-6a), 78.9 (C-2'), 102.6 (CH-4), 106.4 (C-12b), 110.4 (C-8), 112.3 (CH-10), 112.5 (CH-1), 113.8 (C-11a), 116.4 (CH-1'), 129.3 (CH-11), 130.3 (CH-3'), 145.0 (C-2), 149.5 (C-4a), 151.2 (C-3), 158.5 (C-7a), 161.5 (C-9), 191.6 (C-12).

norisojamaicin (**40**): HRESIMS *m/z* 365.1026 $[M+H]^+$ (calcd for $C_{21}H_{17}O_6$, 365.1025). NMR data: ^{1}H NMR (CD_3OD, 600 MHz) δ 1.50 (6H, s, H_3-5'', H_3-6''), 5.87 (1H, d, *J* = 10.1 Hz, H-3''), 5.95 (2H, s, OCH_2O), 6.62 (1H, s, H-2'), 6.65 (1H, s, H-6'), 6.88 (1H, d, *J* = 10.1 Hz, H-4''), 6.91 (0H, d, *J* = 8.5 Hz, H-6), 7.98 (1H, d, *J* = 8.5 Hz, H-5), 8.22 (1H, s, H-2); ^{13}C NMR (CD_3OD, 151 MHz) δ 28.4 (CH_3-5'', CH_3-6''), 79.2 (C-2''), 102.5 (OCH_2O), 102.9 (CH-2'), 110.6 (C-8), 113.4 (CH-6'), 115.5 (CH-4''), 116.6 (CH-6), 119.1 (C-10), 126.1 (C-1'), 127.3 (CH-5), 132.1 (CH-3''), 135.8 (C-4'), 142.0 (C-5'), 150.4 (C-3'), 153.8 (C-9), 155.0 (C-2), 159.4 (C-7), 177.8 (C-4).

3’-hydroxy-4’-*O*-metyhlerrone (**41**): HRESIMS *m/z* 367.118 $[M+H]^+$ (calcd for $C_{21}H_{19}O_6$, 367.1182). NMR data: ^{1}H NMR (CD_3OD, 600 MHz) δ 1.47 (6H, s, H_3-5'', H_3-6''), 3.89 (3H, s, 4'OCH_3), 5.70 (1H, d, *J* = 10.0 Hz, H-3''), 6.22 (1H, s, H-6), 6.74 (1H, d, *J* = 10.0 Hz, H-4''), 6.99 (2H, m, H-5', H-6'), 7.06 (1H, d, *J* = 1.8 Hz, H-2'), 8.17 (1H, s, H-2); ^{13}C NMR (CD_3OD, 151 MHz) δ 28.3 (CH_3-5'', CH_3-6''), 56.2 (4'OCH_3), 79.3 (C-2''), 100.6 (CH-6), 102.4 (C-8), 106.7 (C-10), 112.5 (CH-5'), 115.1 (CH-4''),

117.2 (CH-2'), 121.5 (CH-6'), 124.6 (C-3), 128.7 (CH-3''), 147.3 (C-3'), 149.1 (C-4'), 153.4 (C-9), 154.9 (CH-2), 160.6 (C-7), 163.1 (C-5), 182.2 (C-4).

Isolated Compounds Biological Source

Table S5: Occurrence of each compound in each extract (x = isolated from the corresponding extract)

Compound	*C. palala* roots	*C. grevei* roots bark	*P.erosus* leaves	*D. heterophyllum* u.g
1			x	
2			x	
3			x	
4			x	
5			x	
6			x	
7			x	
8				x
9				x
10				x
11				x
12				x
13				x
14				x
15	x			
16	x			
17		x		
18		x		
19		x		
20		x		
21		x		
22	x			
23		x		
24			x	
25			x	
26			x	
27			x	
28			x	
29			x	
30			x	
31			x	
32			x	
33	x			
34	x			
35	x			
36	x			
37		x	x	x
38	x			
39	x	x		x
40		x		
41		x		

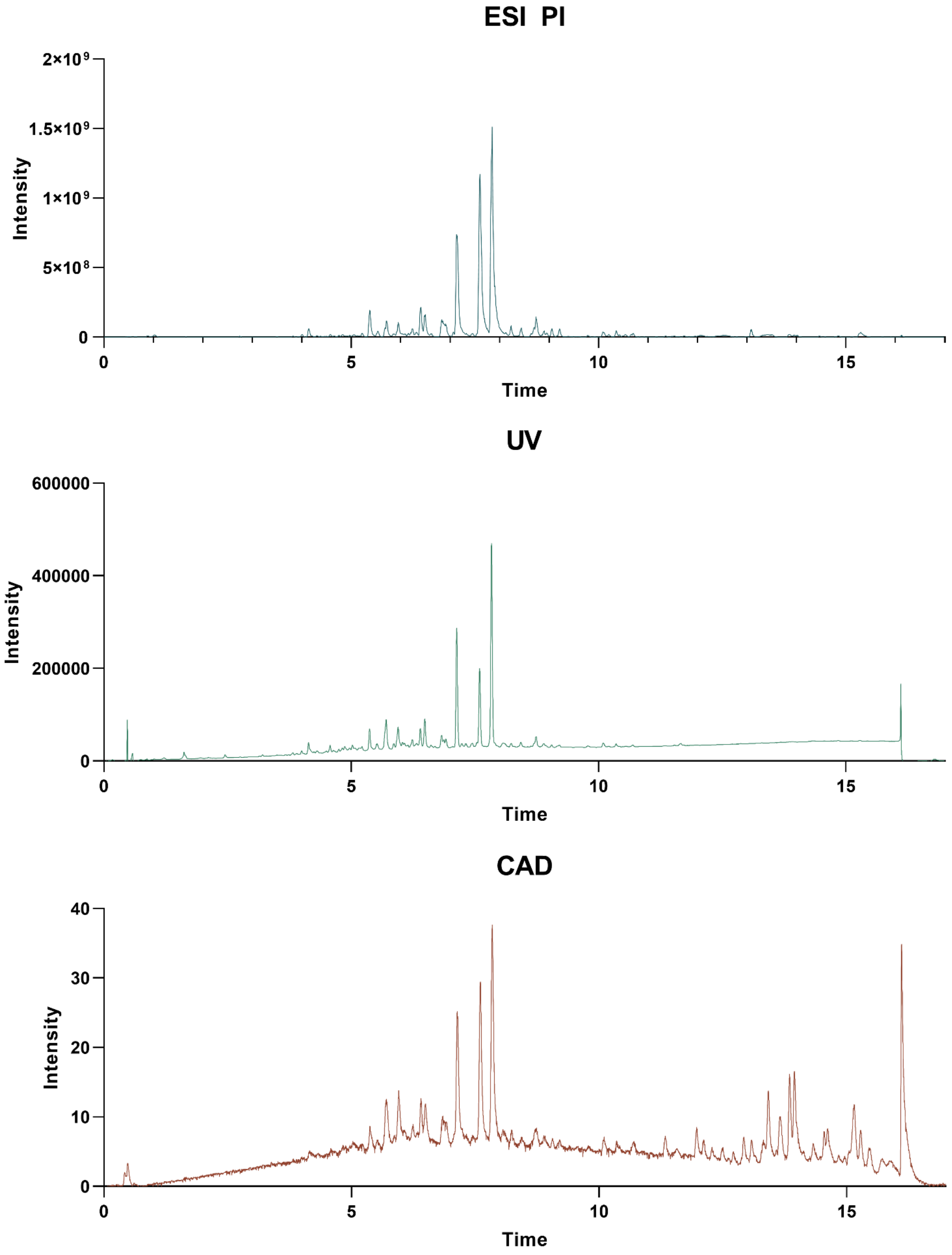

Figure S1: UHPLC-MS-PDA-CAD profile of *Cnestis palala* branches EtOAc extract.

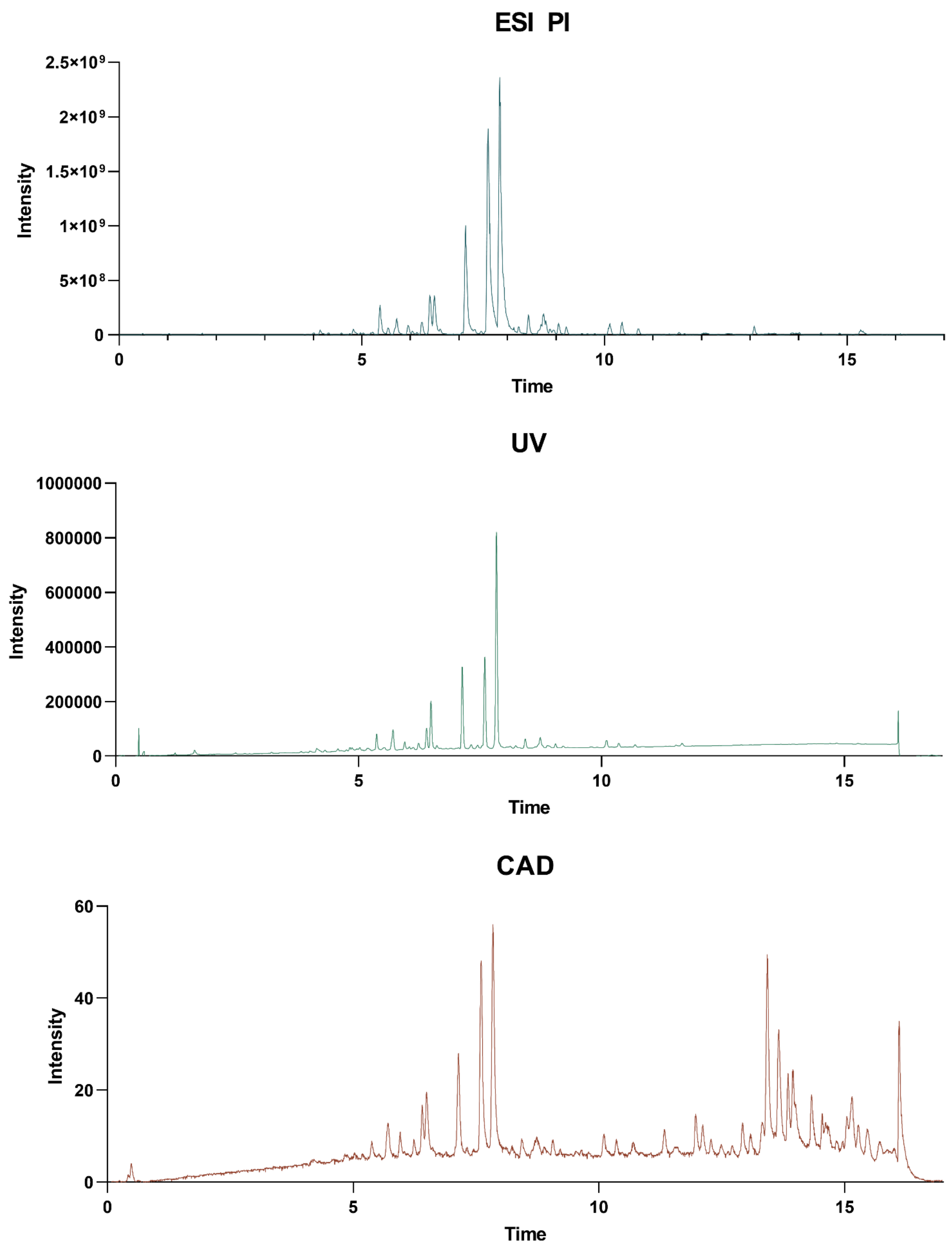

Figure S2: UHPLC-MS-PDA-CAD profile of *Cnestis palala* roots EtOAc extract.

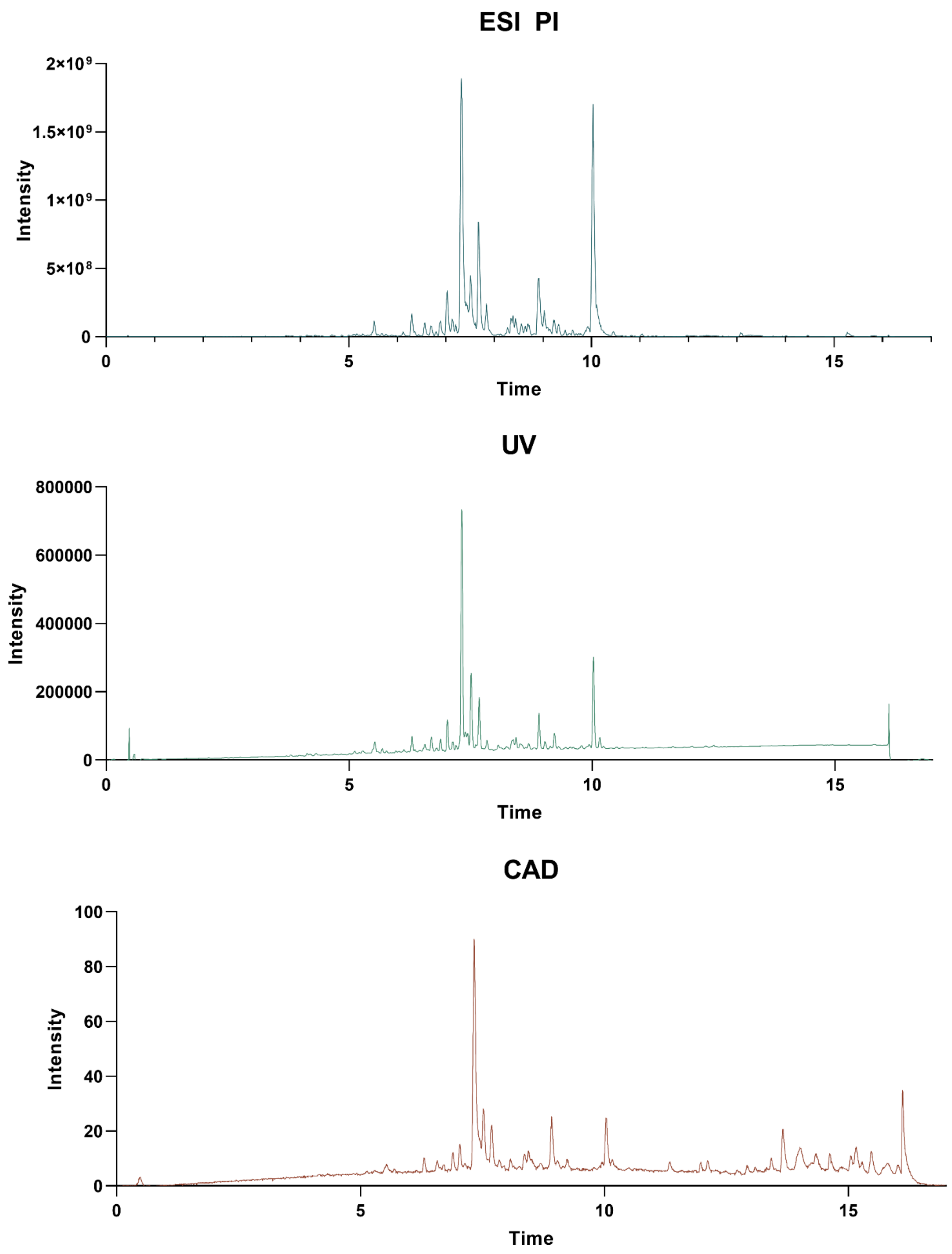

Figure S3: UHPLC-MS-PDA-CAD profile of *Chadsia grevei* roots bark EtOAc extract.

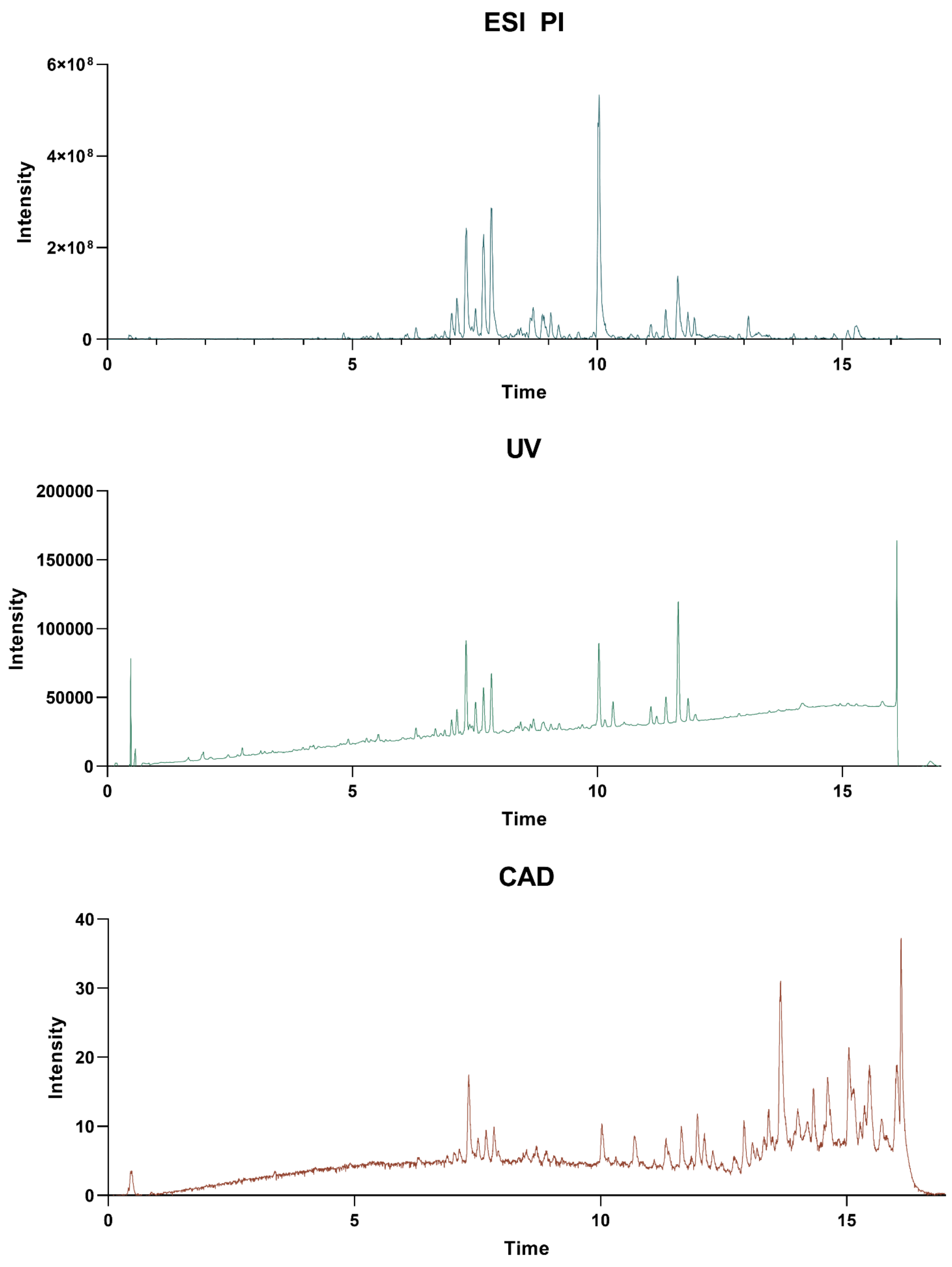

Figure S4: UHPLC-MS-PDA-CAD profile of *Chadsia grevei* trunk bark EtOAc extract.

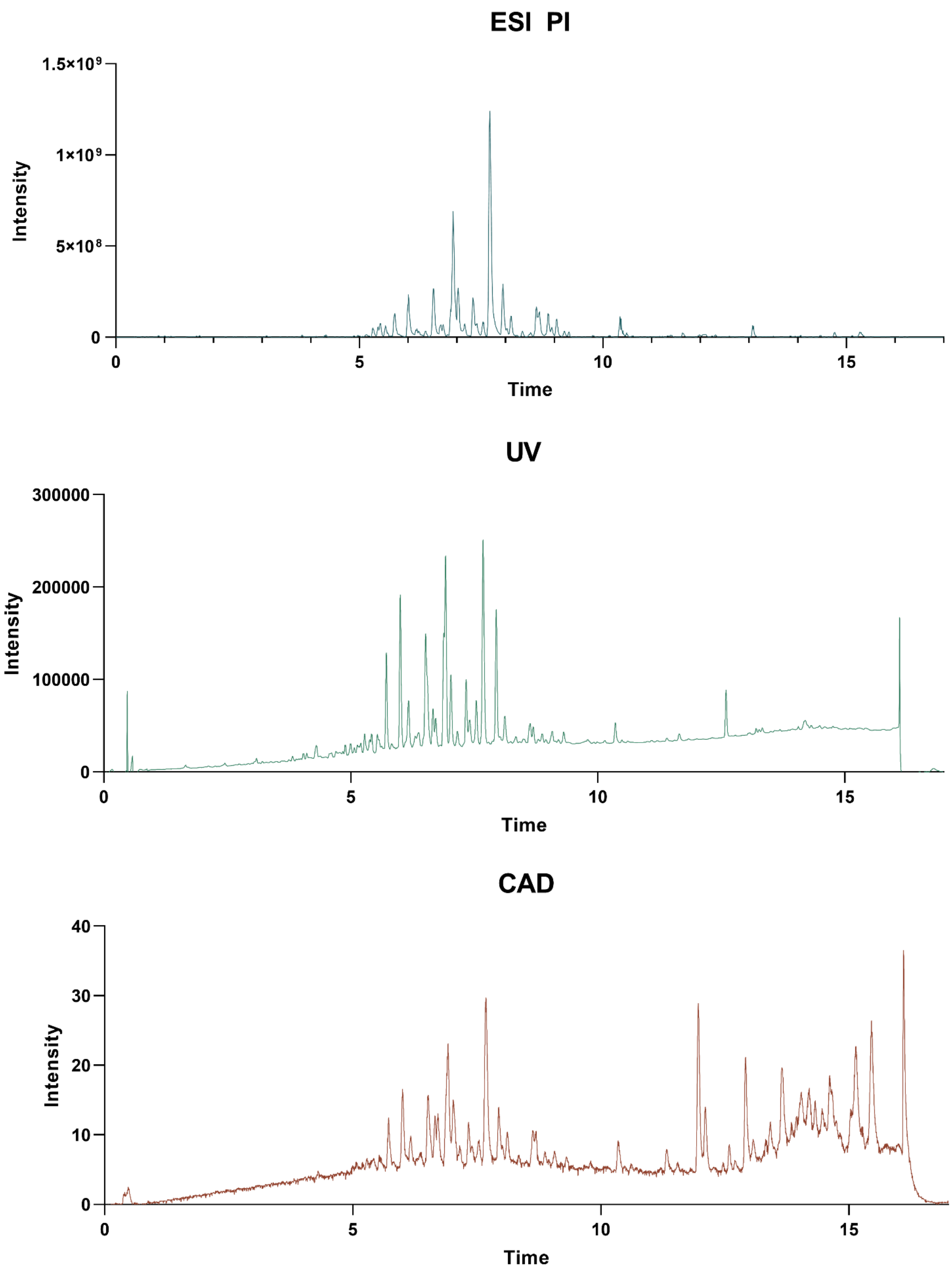

Figure S5: UHPLC-MS-PDA-CAD profile of *Pachyrhizus erosus* leaves EtOAc extract.

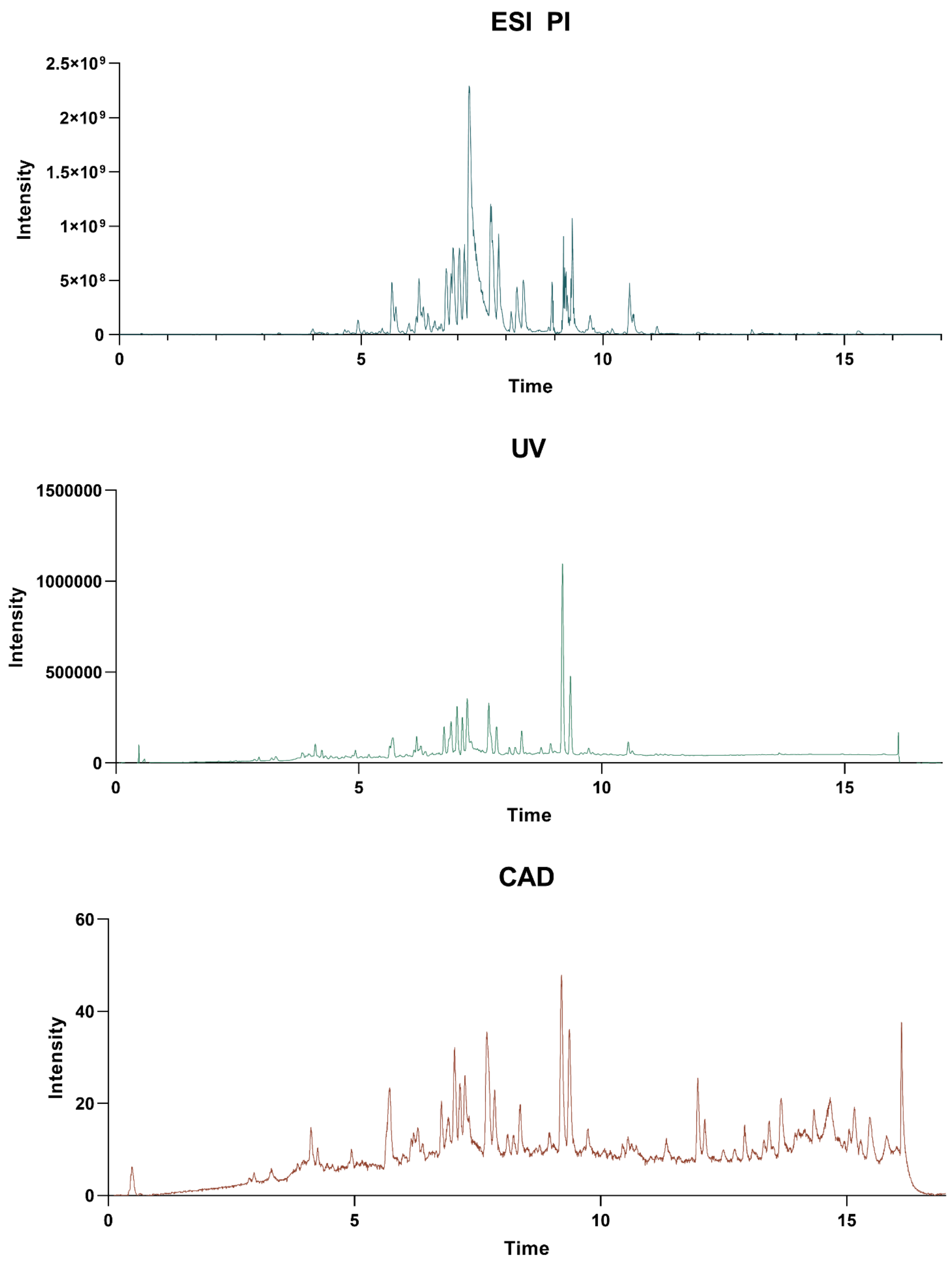

Figure S6: UHPLC-MS-PDA-CAD profile of *Desmodium heterophyllum* underground parts EtOAc extract.

Ion-Identity Molecular Network

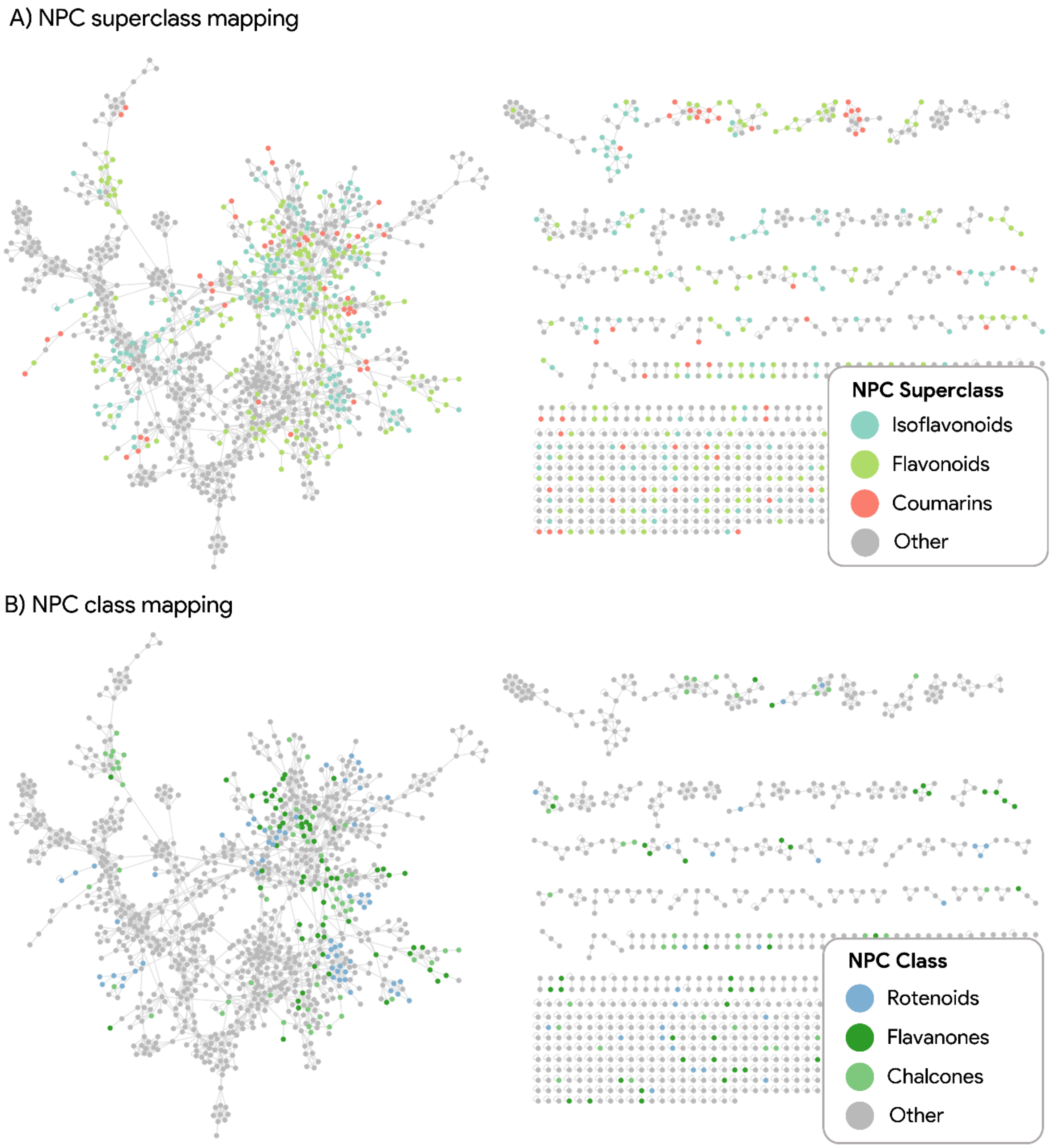

Figure S7: IIN MN of the six active extracts with mapping of the three main chemical NPClassifier superclasses (A) and classes (B).

Semi-Preparative HPLC chromatograms

A) *Cnestis palala* roots

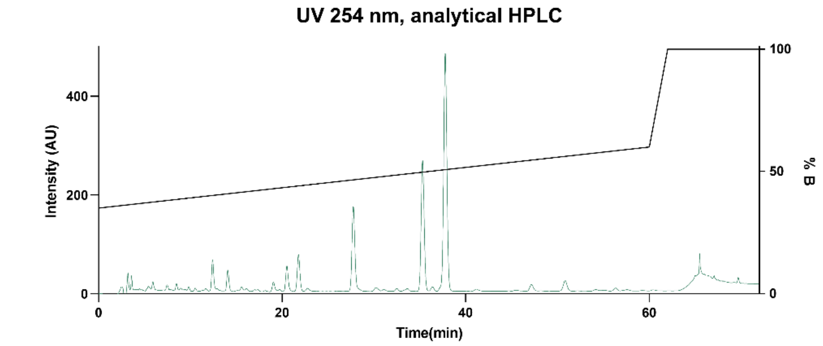

m = 0.05 mg

Waters® Xbridge C18
5µm, 4.6 x 250mm

A: H_2O + 0.1% FA
B: MeCN + 0.1% FA

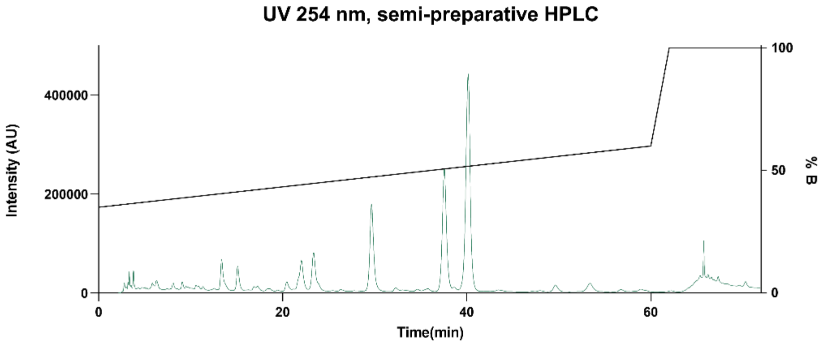

m = 38 mg

Waters® Xbridge C18
5µm, 19 x 250mm

A: H_2O + 0.1% FA
B: MeCN + 0.1% FA

B) *Chadsia grevei* roots bark

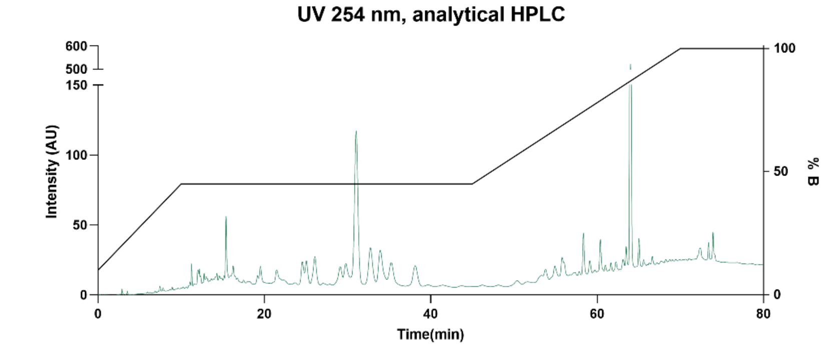

m = 0.05 mg

Waters® Xbridge C18
5µm, 4.6 x 250mm

A: H_2O + 0.1% FA
B: MeCN + 0.1% FA

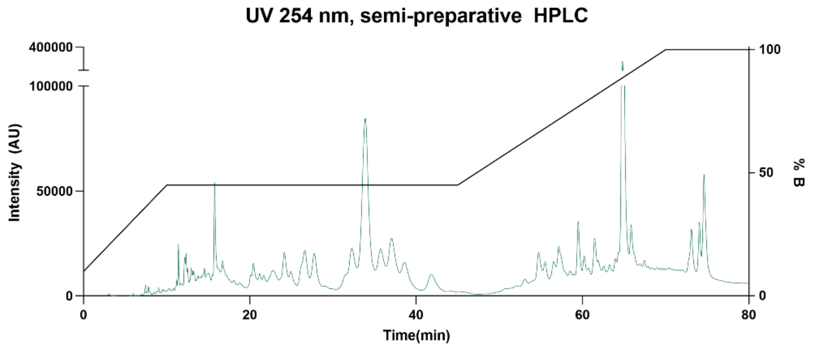

m = 40 mg

Waters® Xbridge C18
5µm, 19 x 250mm

A: H_2O + 0.1% FA
B: MeCN + 0.1% FA

Figure S8: HPLC chromatograms (analytical and semi-preparative scale) of *Cnestis palala* roots (A) and *Chadsia grevei* roots bark (B) extracts. *m = amount of extract injected*.

A) *Pachyrhizus erosus leaves*

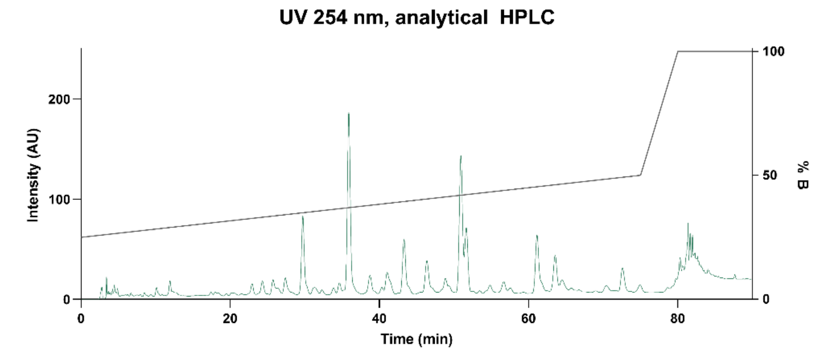

m = 0.05 mg

Waters® Xbridge C18
5µm, 4.6 x 250mm

A: H_2O + 0.1% FA
B: MeCN + 0.1% FA

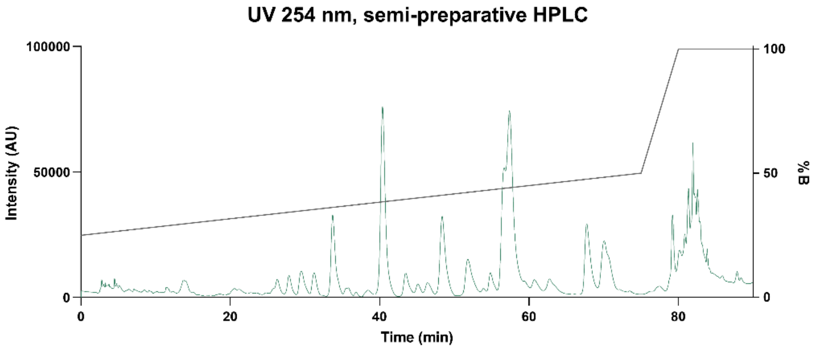

m = 39 mg

Waters® Xbridge C18
5µm, 19 x 250mm

A: H_2O + 0.1% FA
B: MeCN + 0.1% FA

B) *Desmodium heterophyllum* underground parts

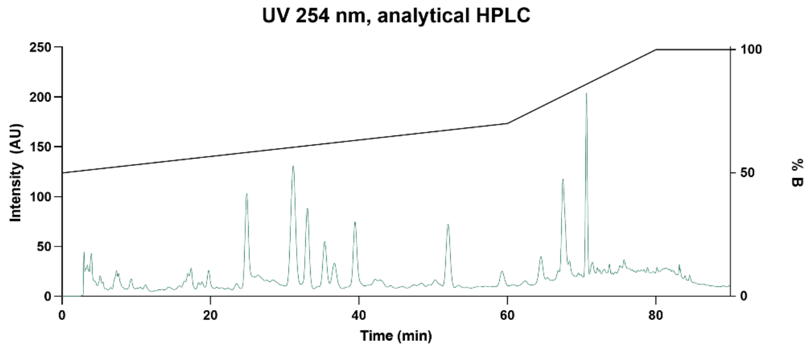

m = 0.05 mg

Waters® Xbridge C18
5µm, 4.6 x 250mm

A: H_2O + 0.1% FA
B: MeOH + 0.1% FA

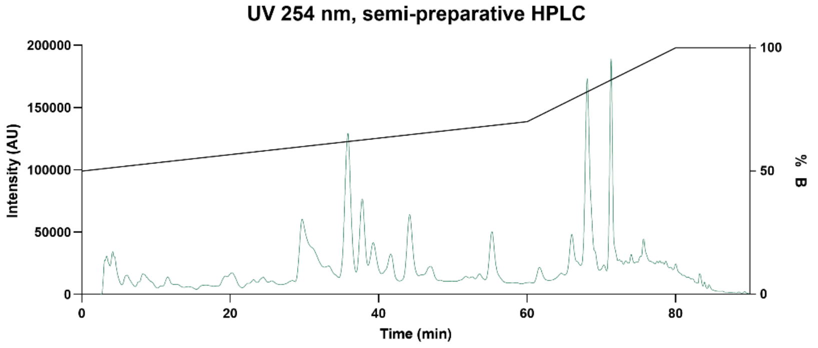

m = 43 mg

Waters® Xbridge C18
5µm, 19 x 250mm

A: H_2O + 0.1% FA
B: MeOH + 0.1% FA

Figure S9: HPLC chromatograms (analytical and semi-preparative scale) of *Pachyrhizus erosus* leaves(A) and *Desmodium heterophyllum* underground parts (B) extracts. *m = amount of extract injected*.

NMR Spectra Of New Compounds

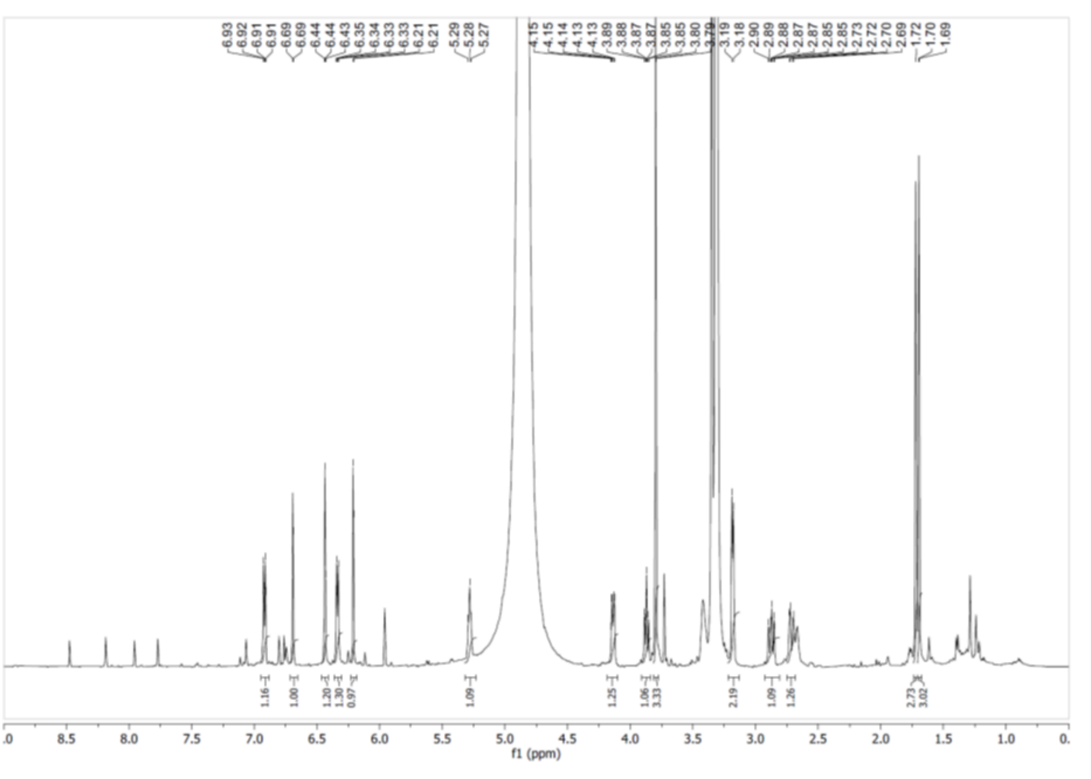

Figure S10: 1H NMR spectrum of compound **1** in CD3OD at 600 MHz

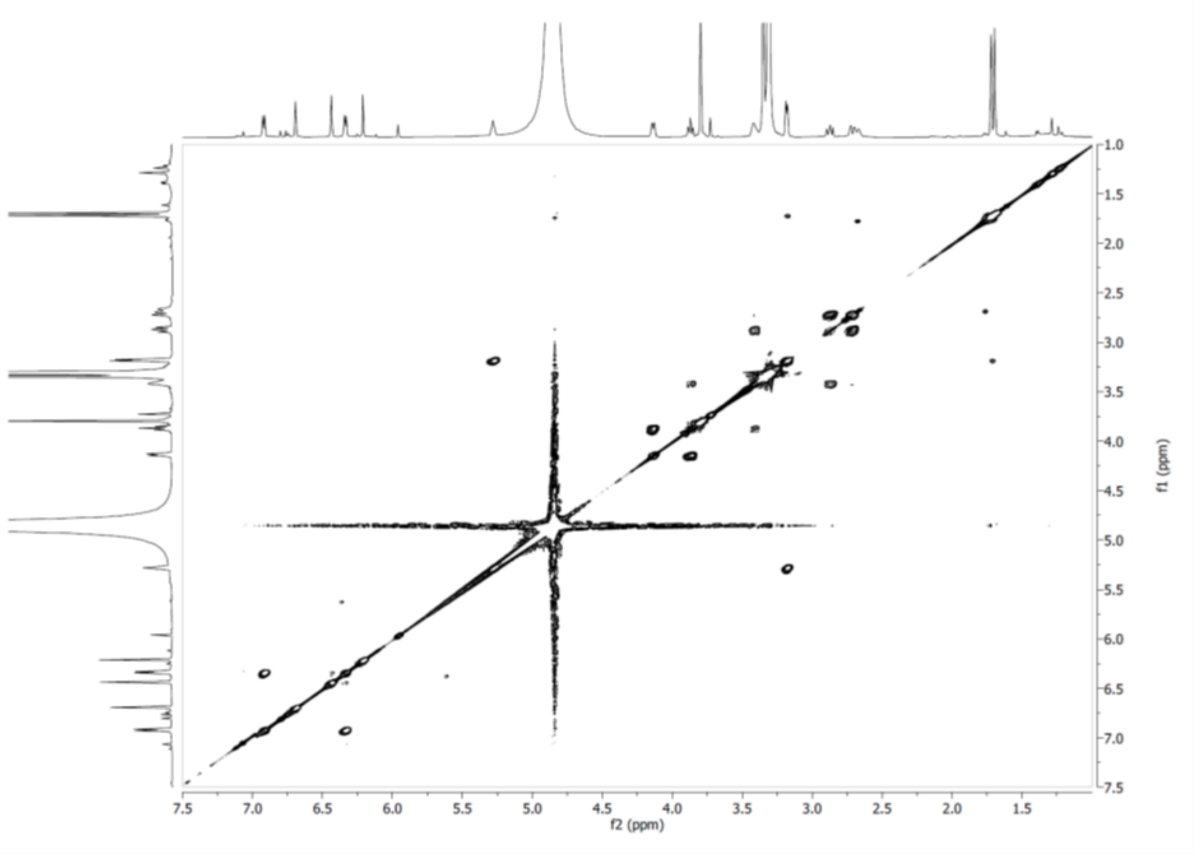

Figure S11: COSY NMR spectrum of compound **1** in CD_3OD

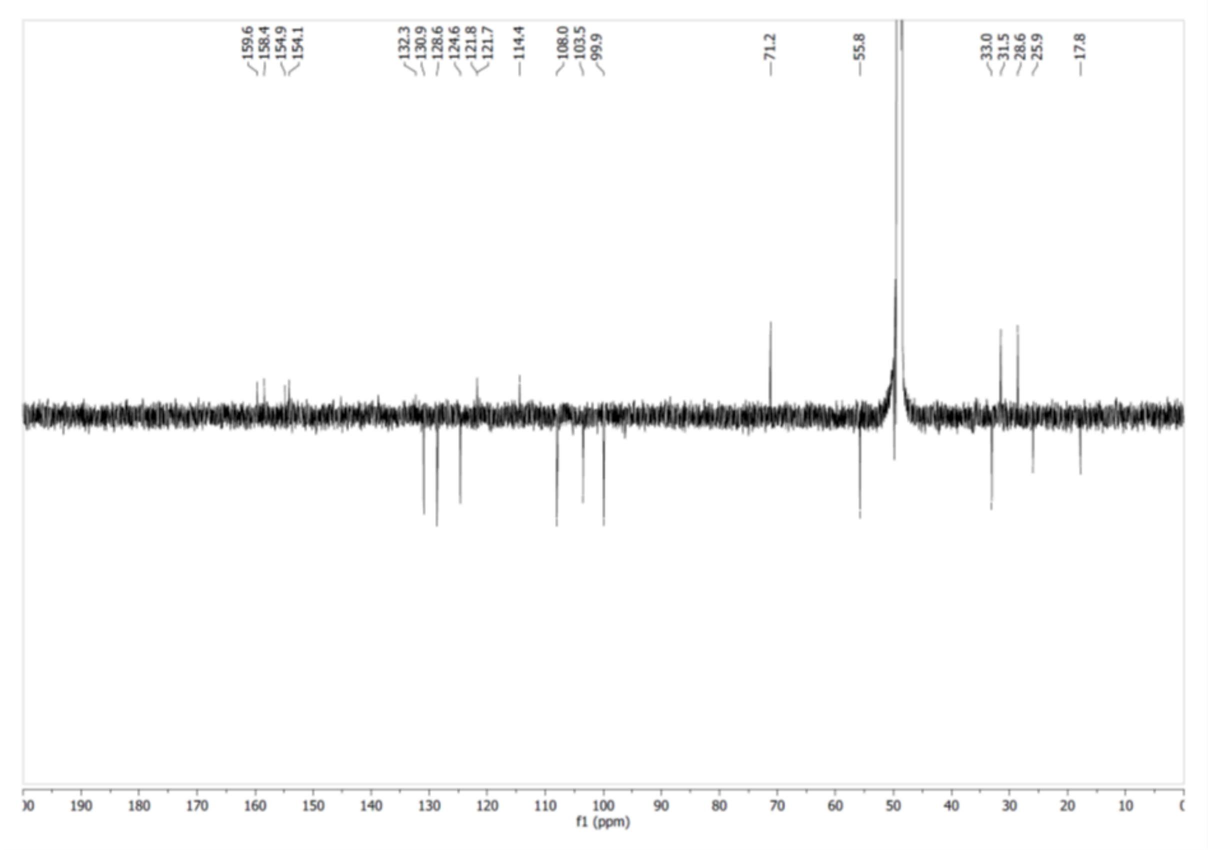

Figure S12: ^{13}C-DEPTQ NMR spectrum of compound **1** in CD_3OD at 151 MHz

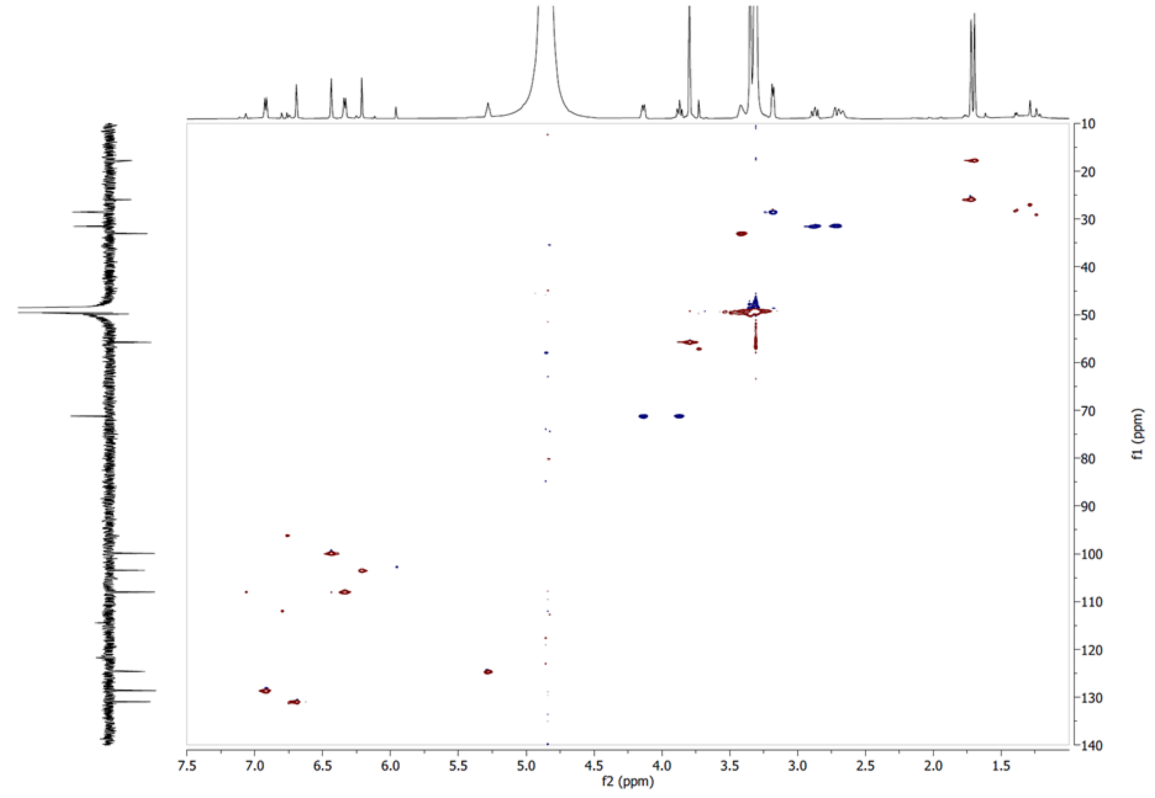

Figure S13: Edited HSQC NMR spectrum of compound **1** in CD_3OD

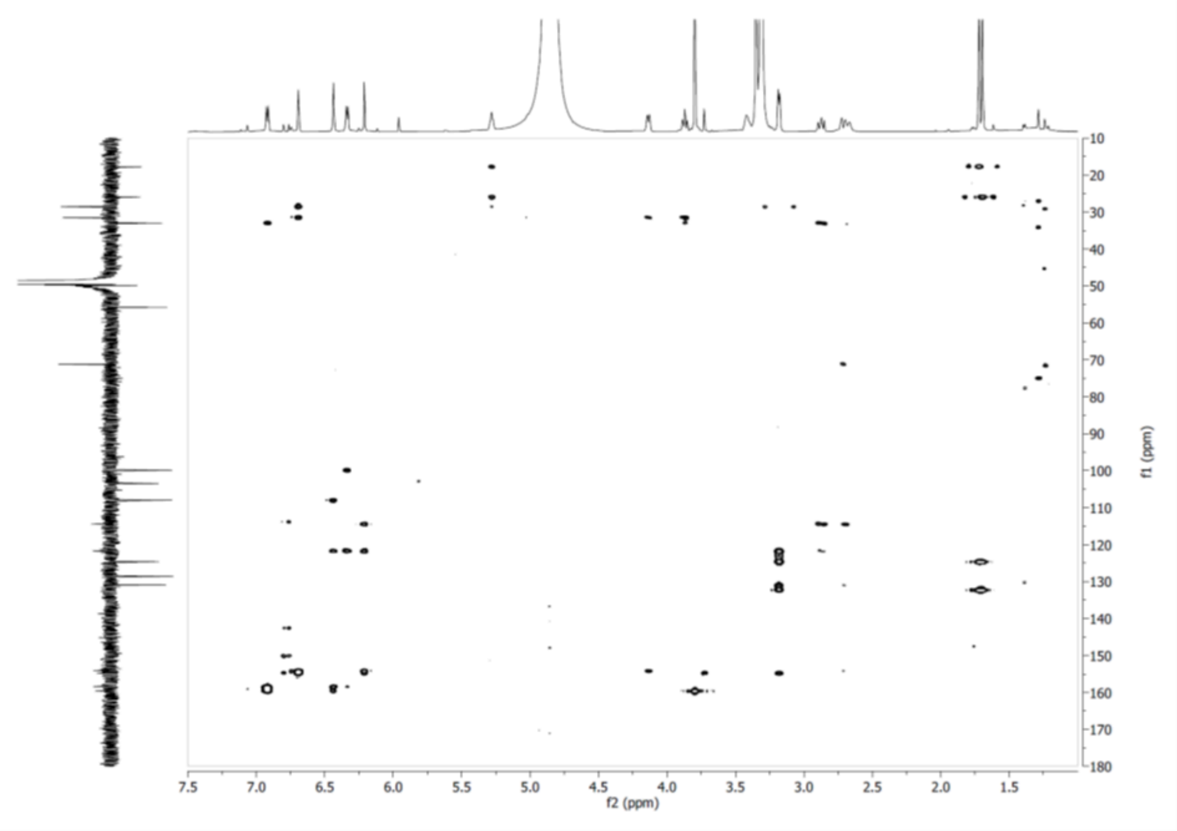

Figure S14: HMBC NMR spectrum of compound **1** in CD_3OD

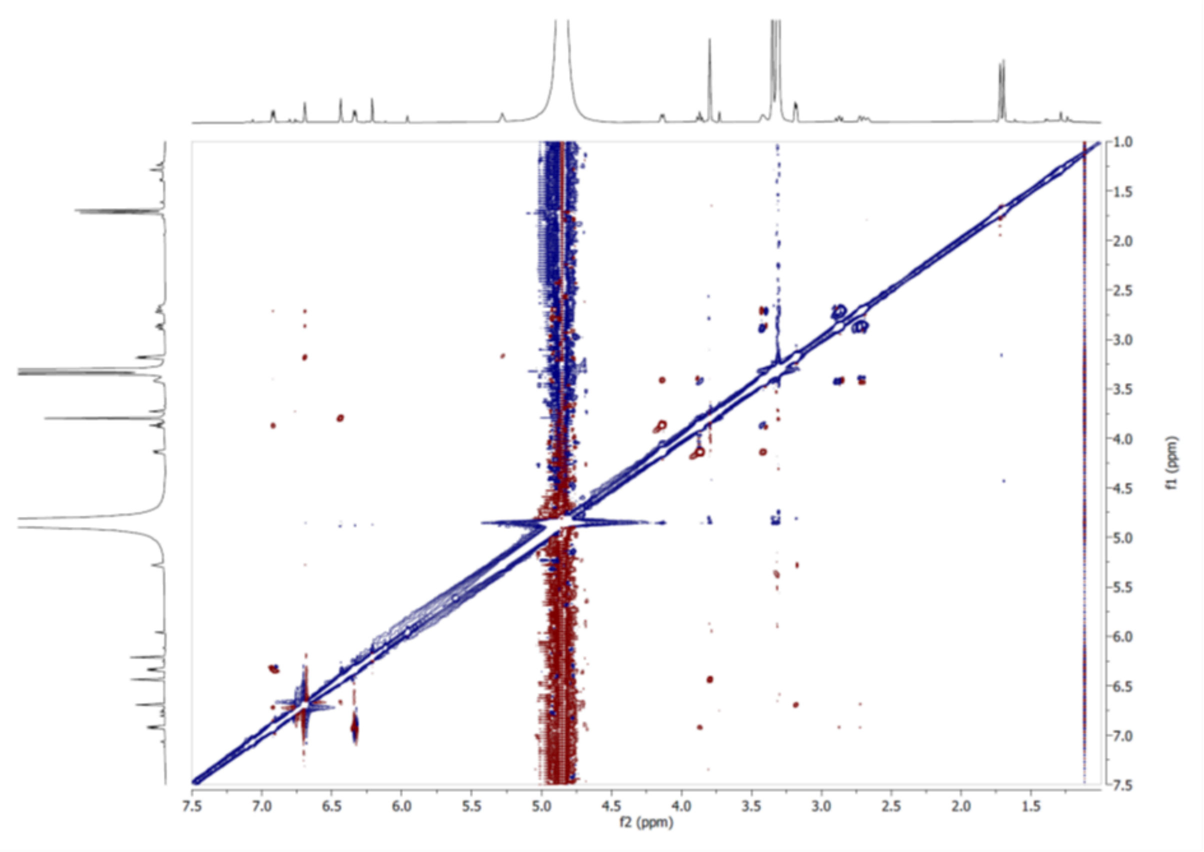

Figure S15: ROESY NMR spectrum of compound **1** in CD_3OD

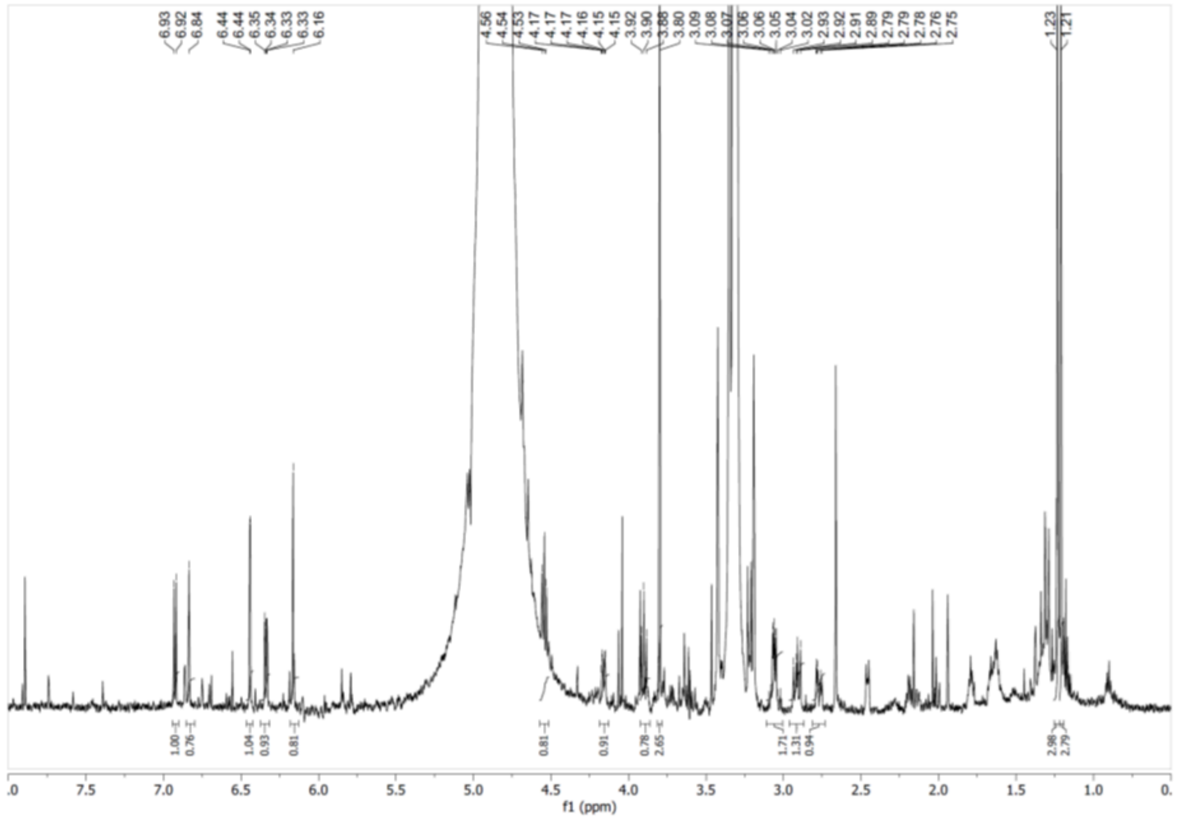

Figure S16: ^{1}H NMR spectrum of compound **6** in CD_3OD at 600 MHz

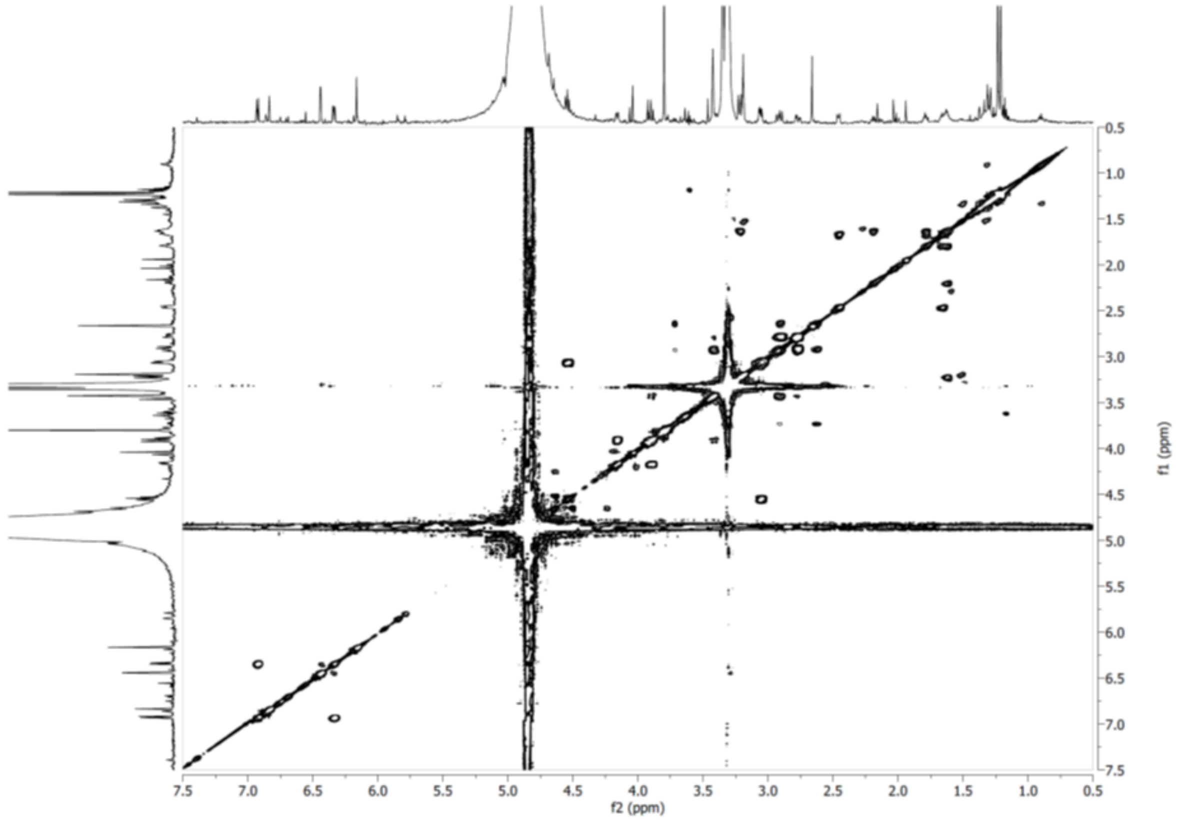

Figure S17: COSY NMR spectrum of compound **6** in CD_3OD

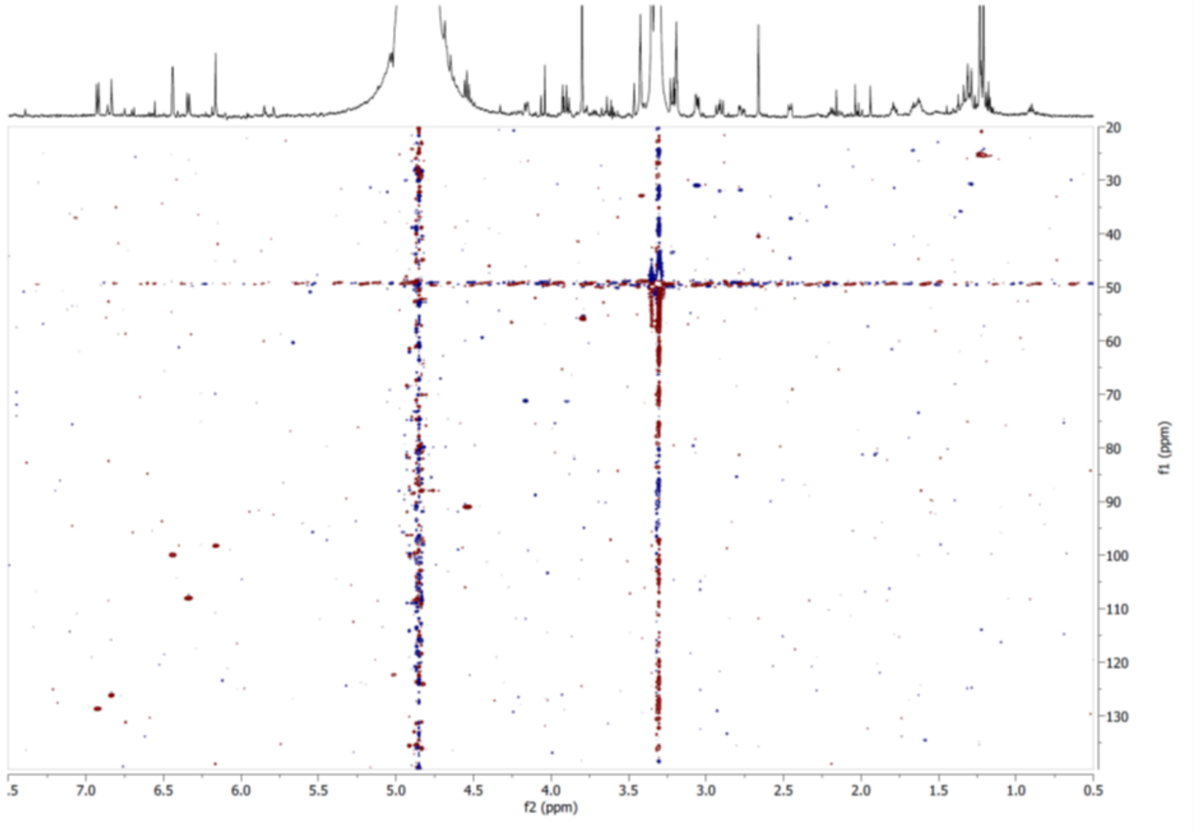

Figure S18: Edited HSQC NMR spectrum of compound **6** in CD_3OD

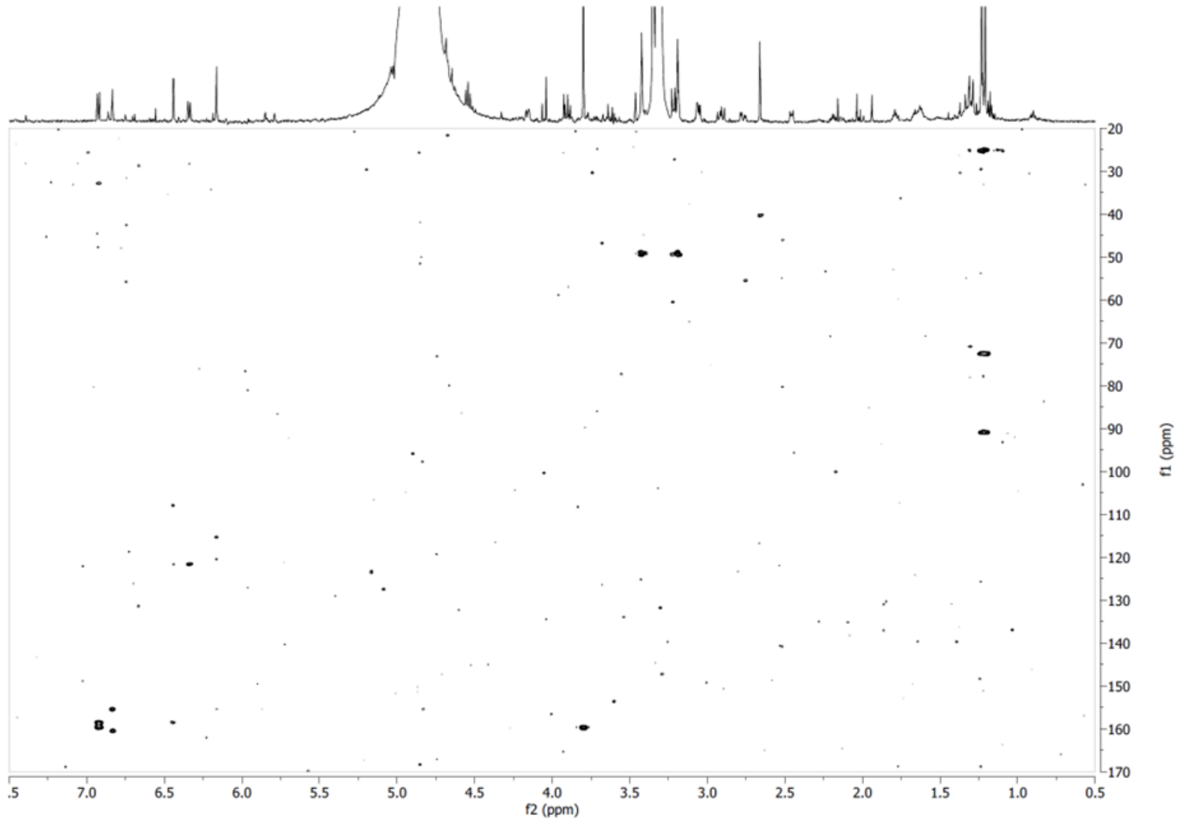

Figure S19: HMBC NMR spectrum of compound **6** in CD_3OD

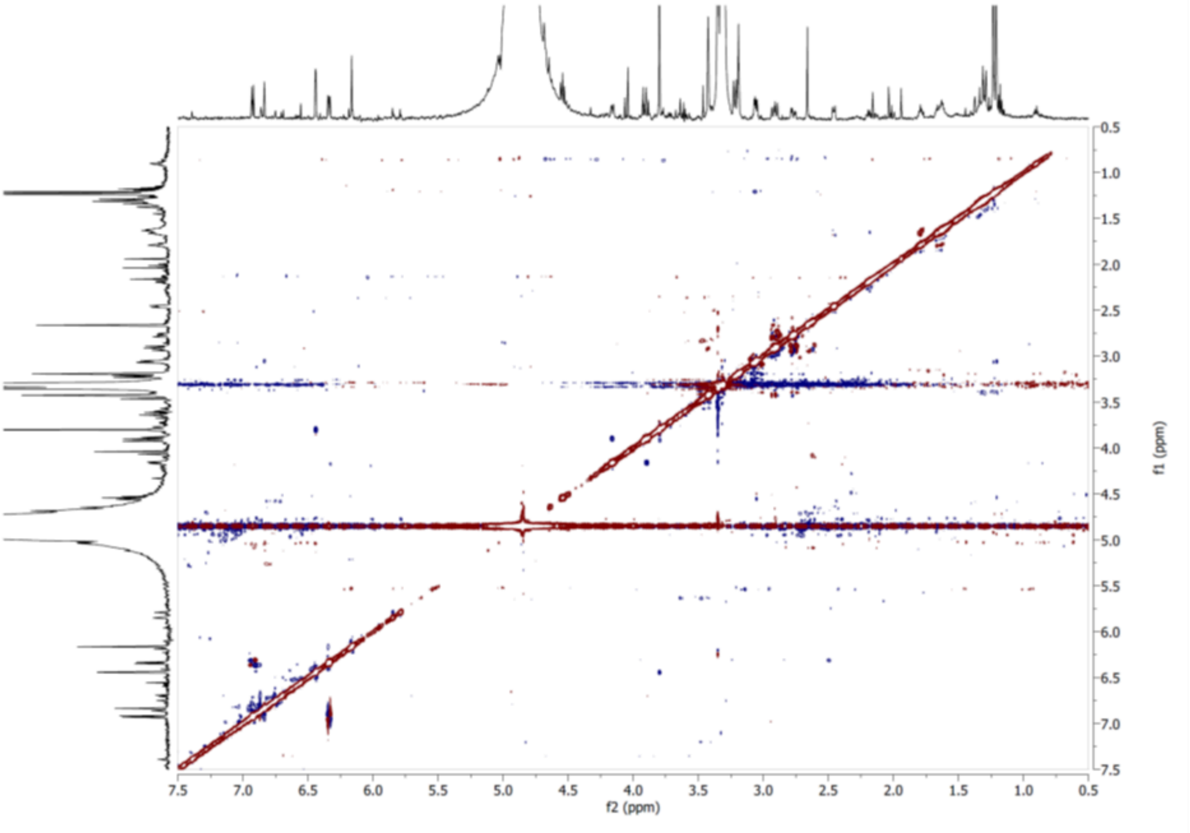

Figure S20: ROESY NMR spectrum of compound **6** in CD_3OD

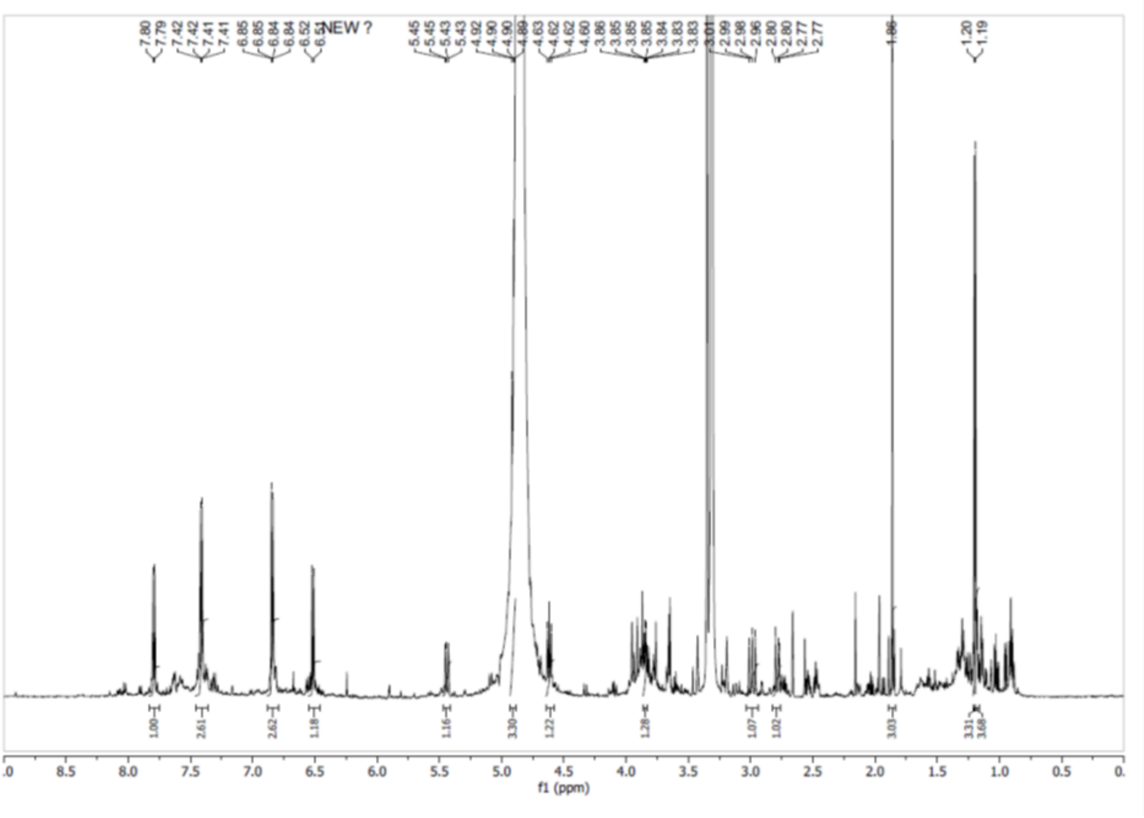

Figure S21: ^{1}H NMR spectrum of compound **12** in CD_3OD at 600 MHz

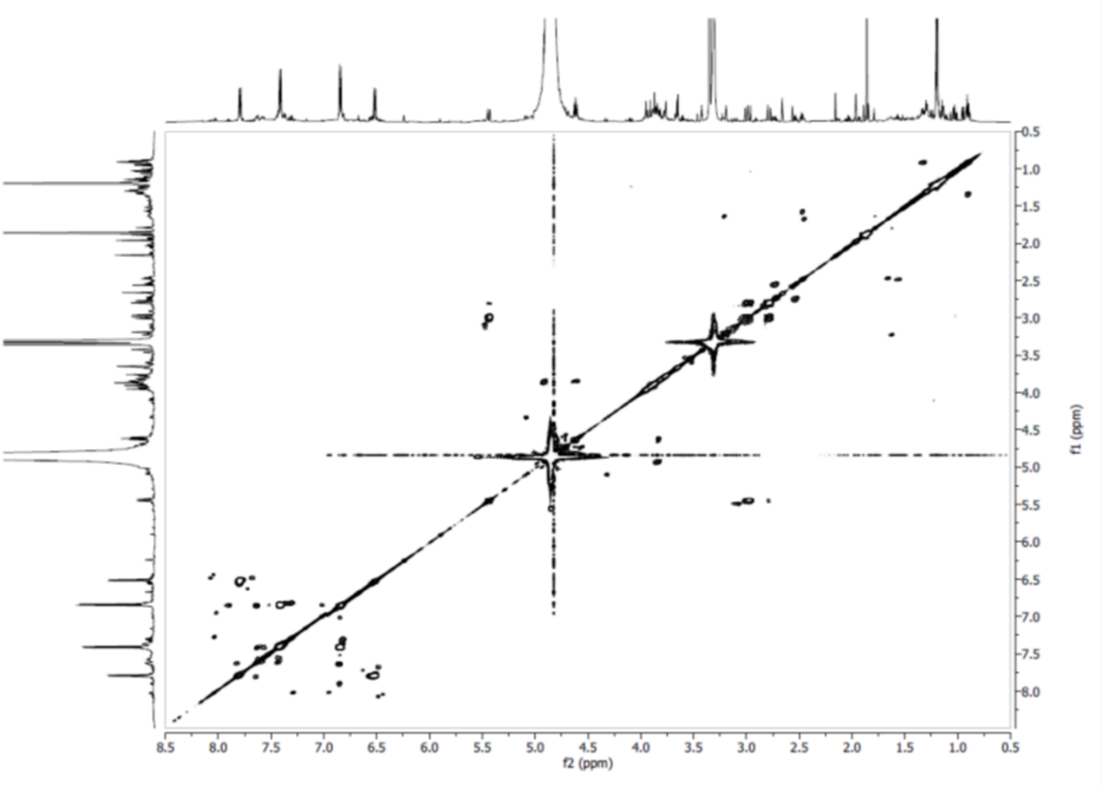

Figure S22: COSY NMR spectrum of compound **12** in CD_3OD

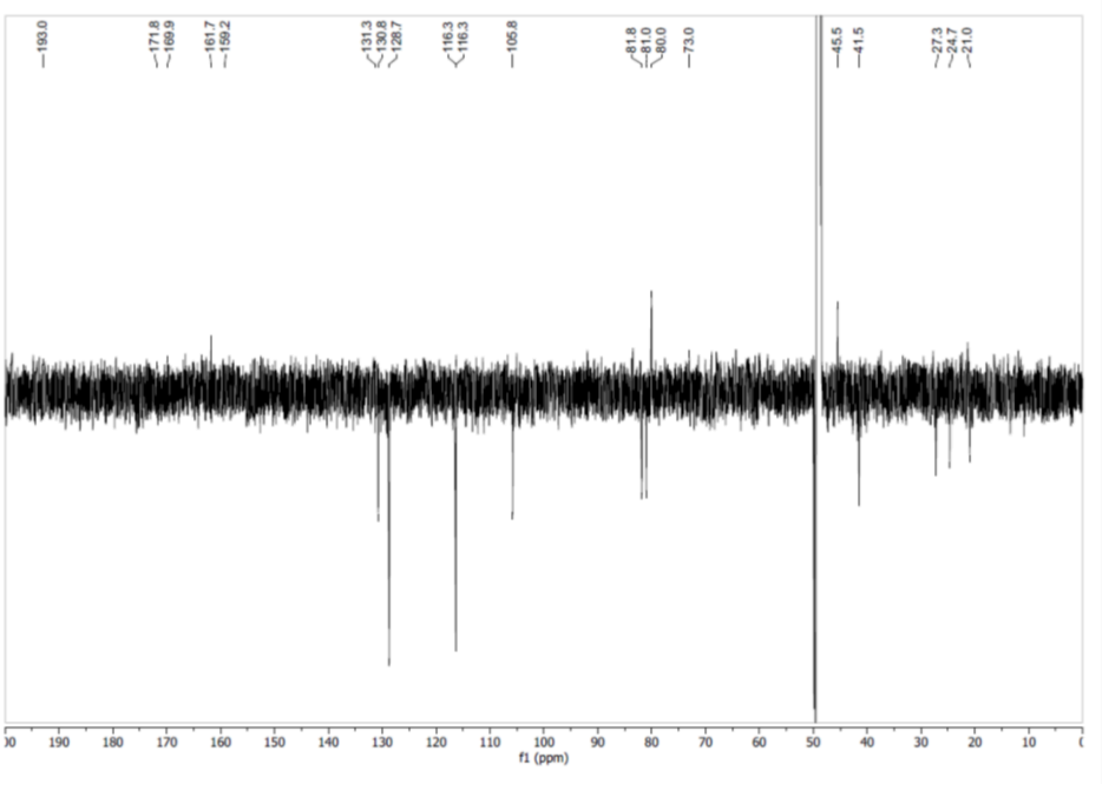

Figure S23: ^{13}C-DEPTQ NMR spectrum of compound **12** in CD_3OD at 151 MHz

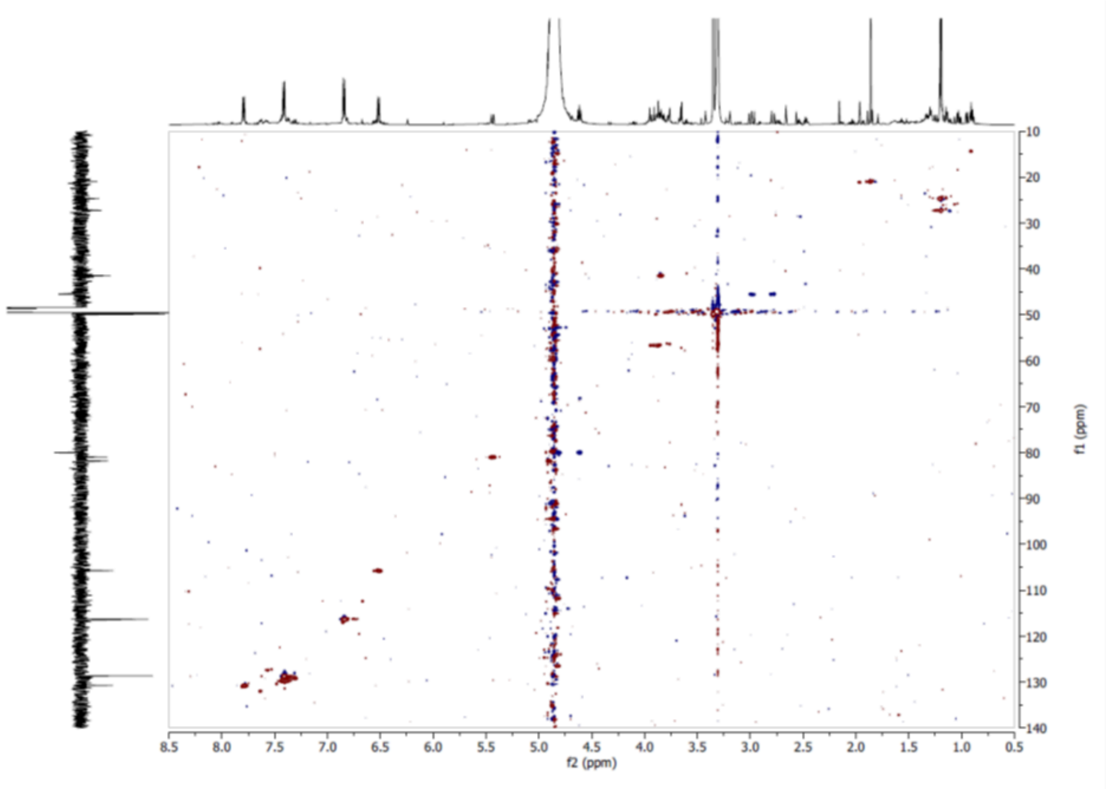

Figure S23: Edited HSQC NMR spectrum of compound **12** in CD_3OD

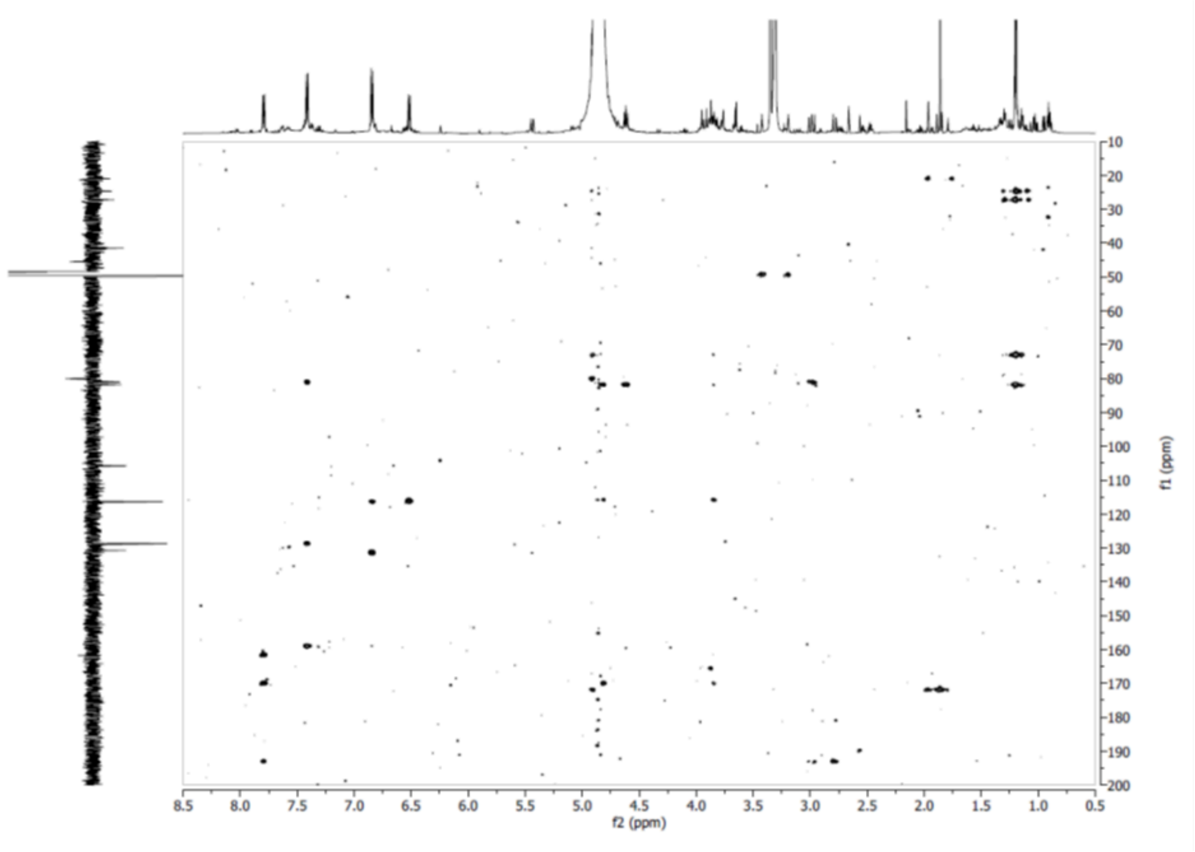

Figure S24: HMBC NMR spectrum of compound **12** in CD_3OD

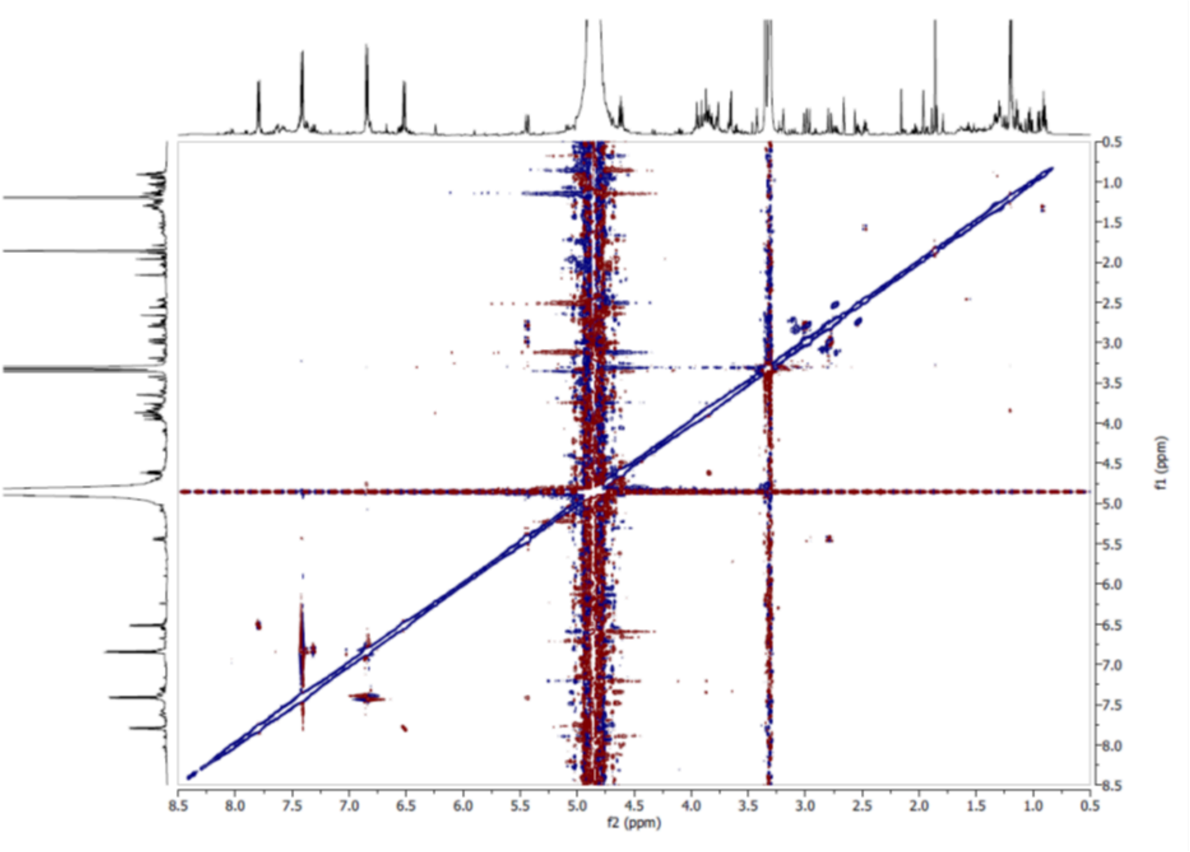

Figure S25: ROESY NMR spectrum of compound **12** in CD_3OD

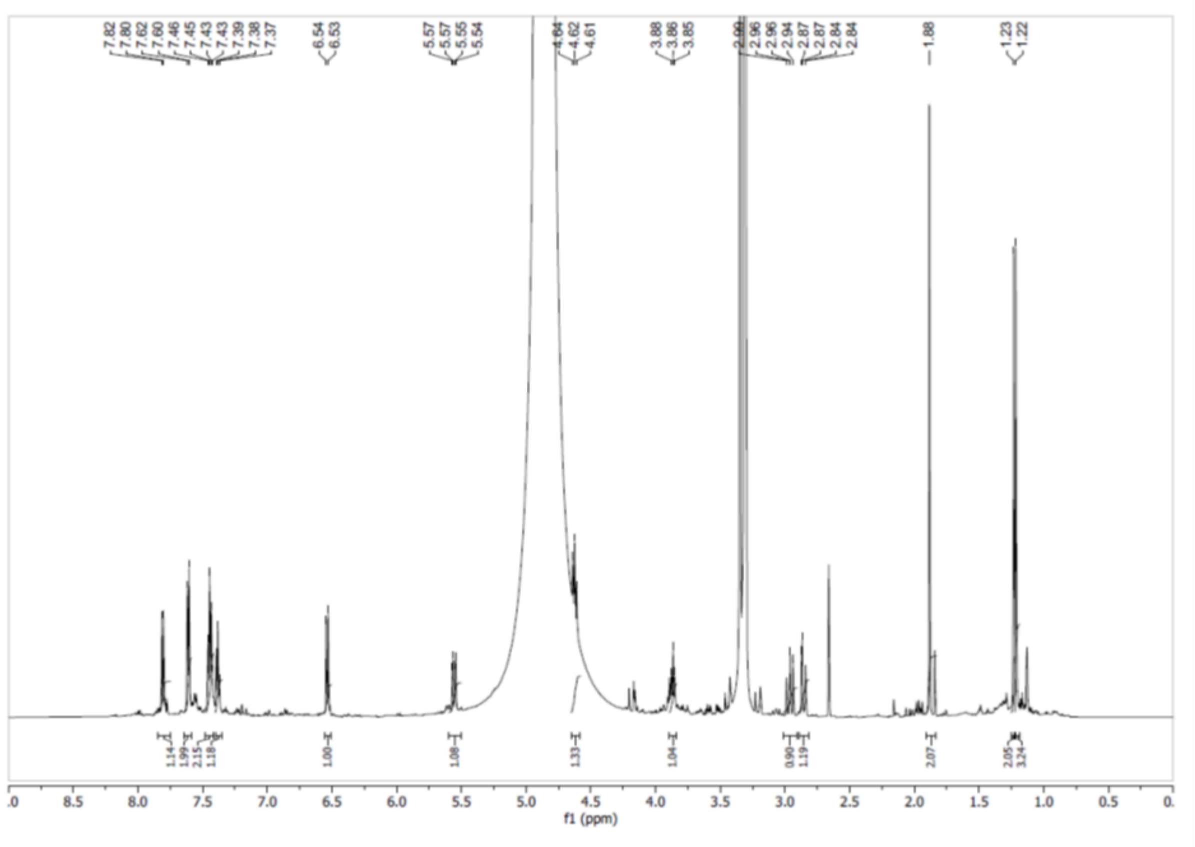

Figure S26: ^{1}H NMR spectrum of compound **13** in CD_3OD at 600 MHz

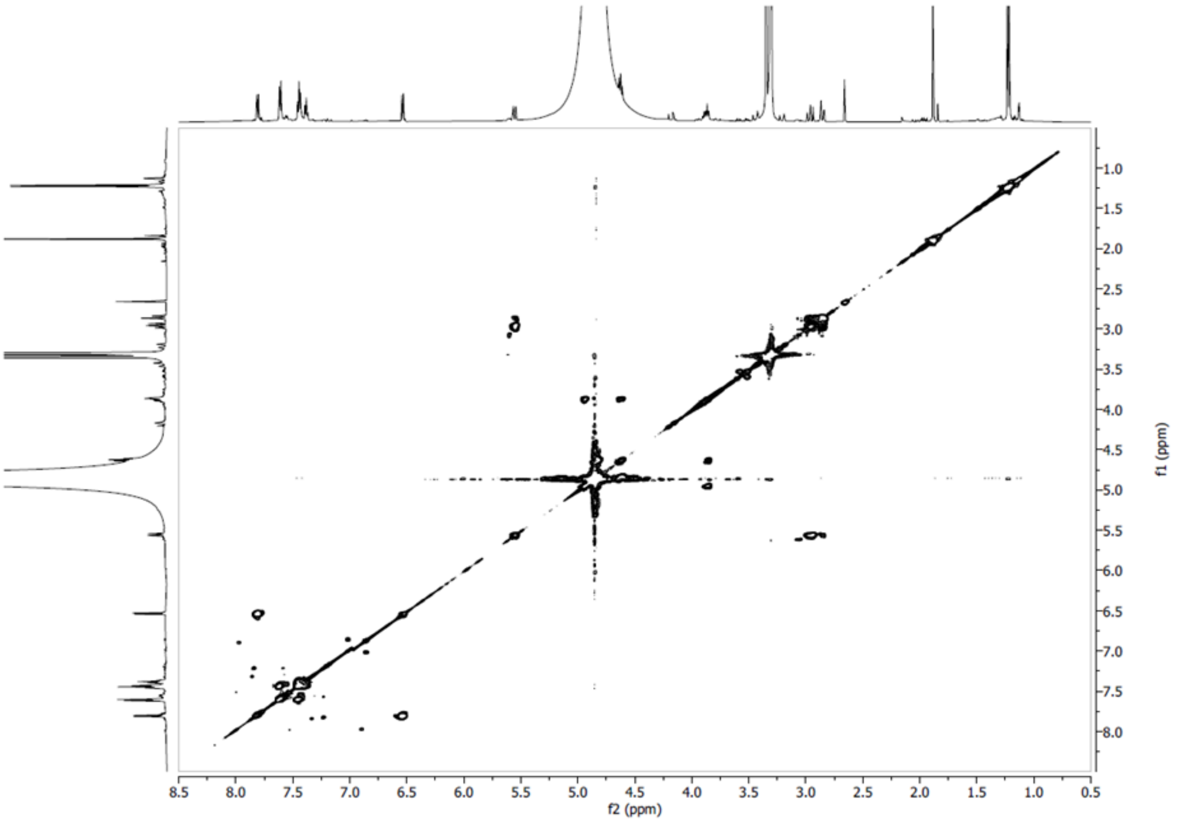

Figure S27: COSY NMR spectrum of compound **13** in CD_3OD

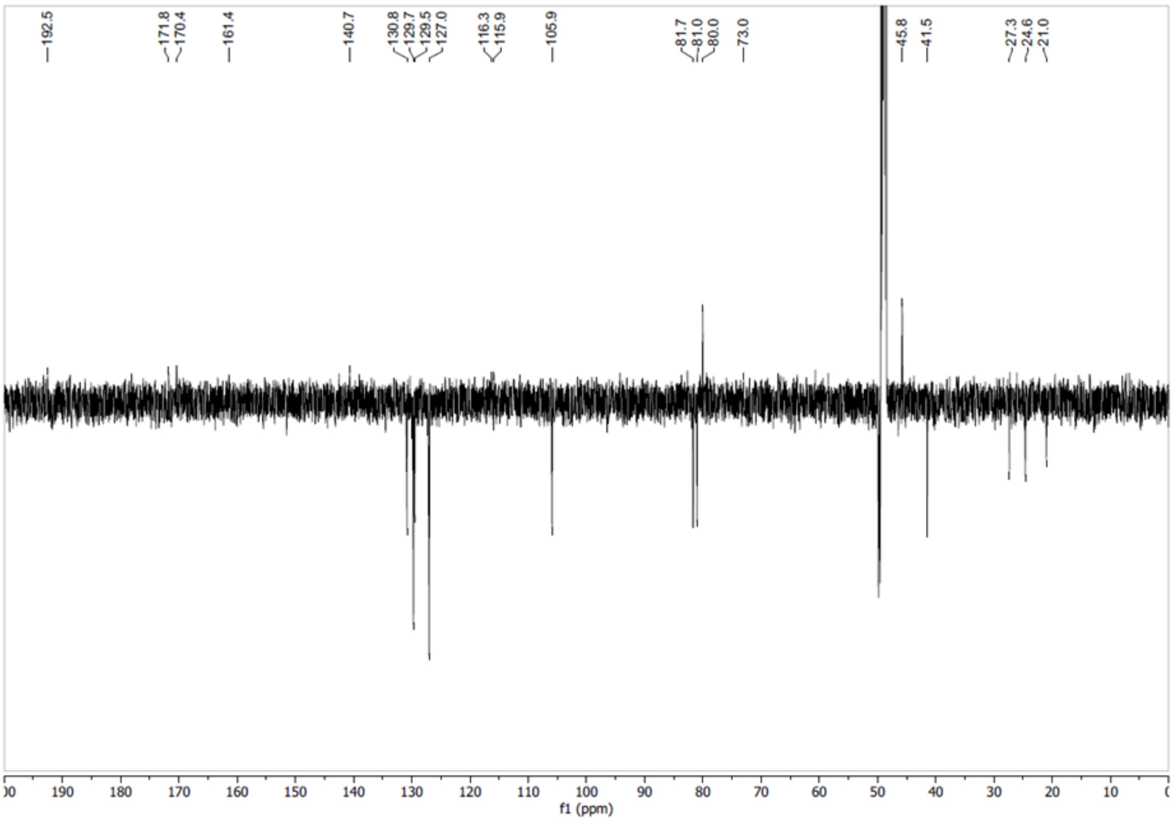

Figure S27: ^{13}C-DEPTQ NMR spectrum of compound **13** in CD_3OD at 151 MHz

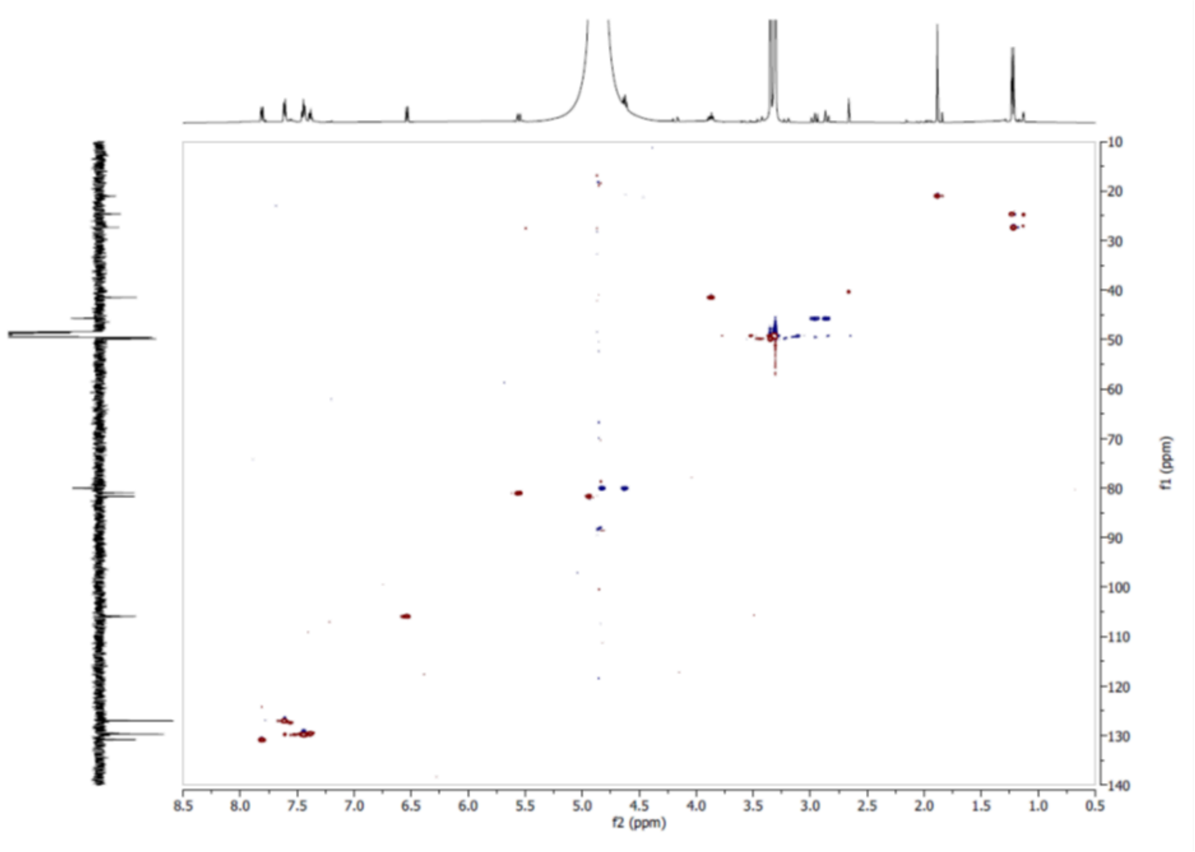

Figure S28: Edited HSQC NMR spectrum of compound **13** in CD_3OD

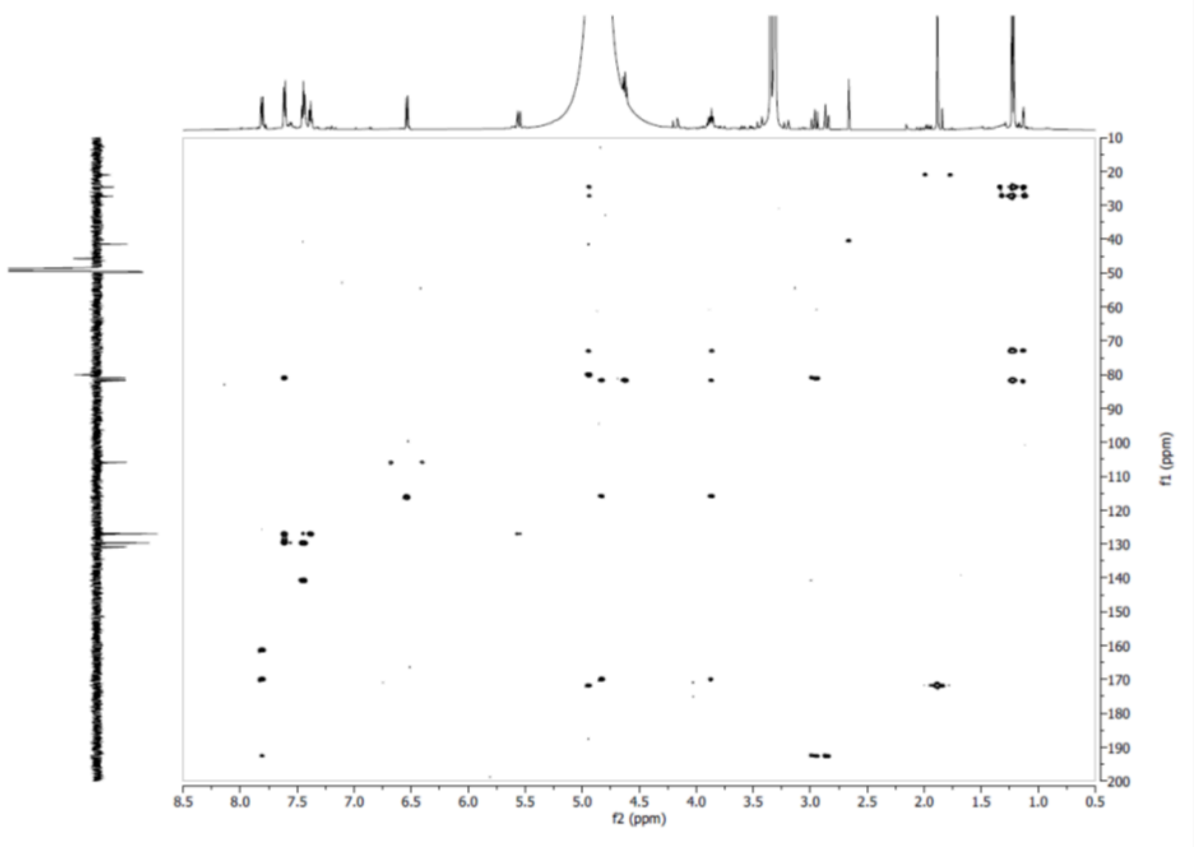

Figure S29: HMBC NMR spectrum of compound **13** in CD_3OD

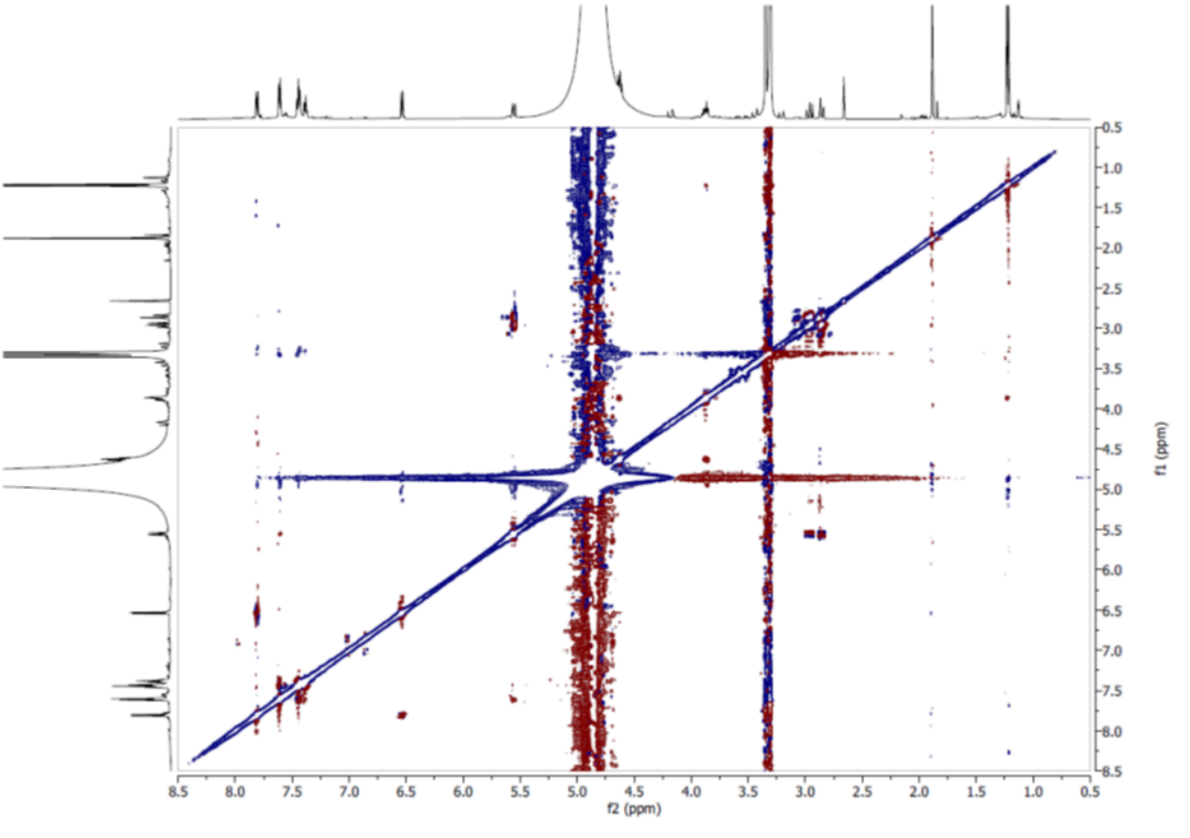

Figure S30: ROESY NMR spectrum of compound **13** in CD_3OD

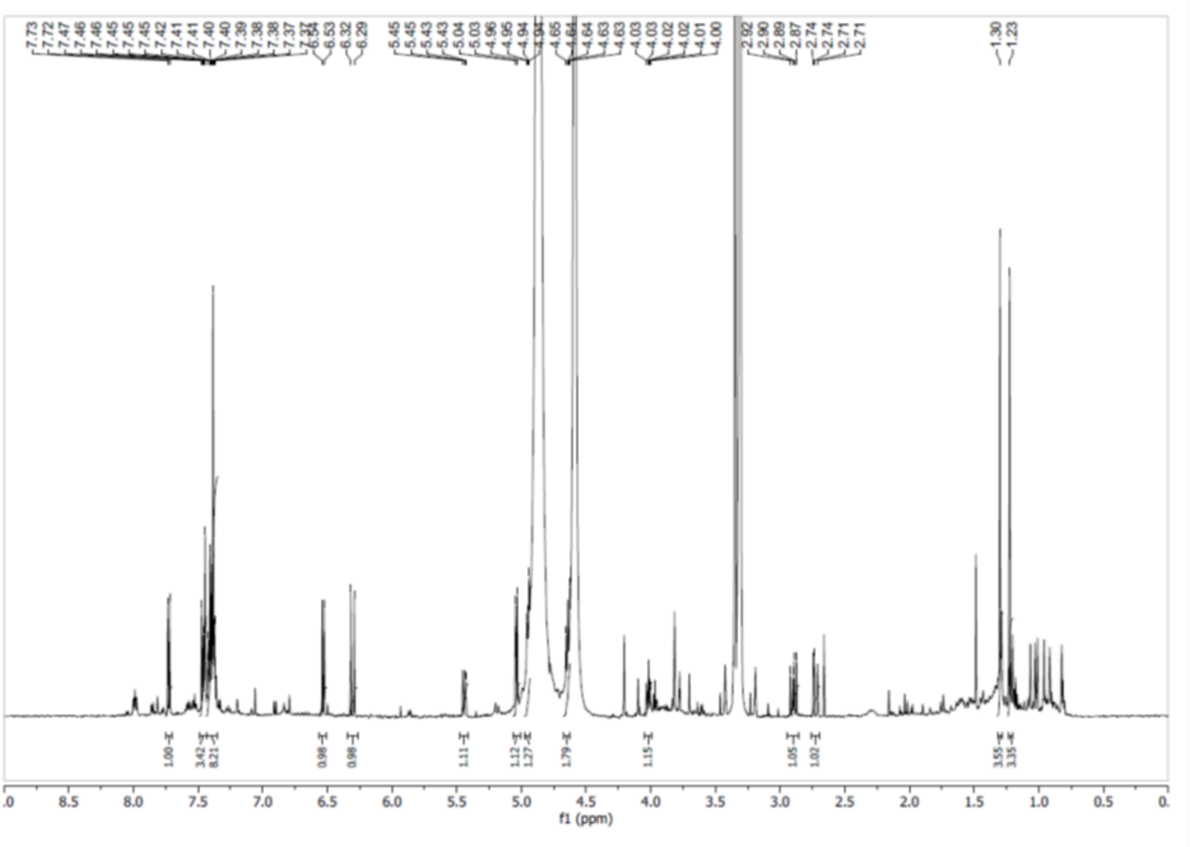

Figure S31: ^{1}H NMR spectrum of compound **14** in CD_3OD at 600 MHz

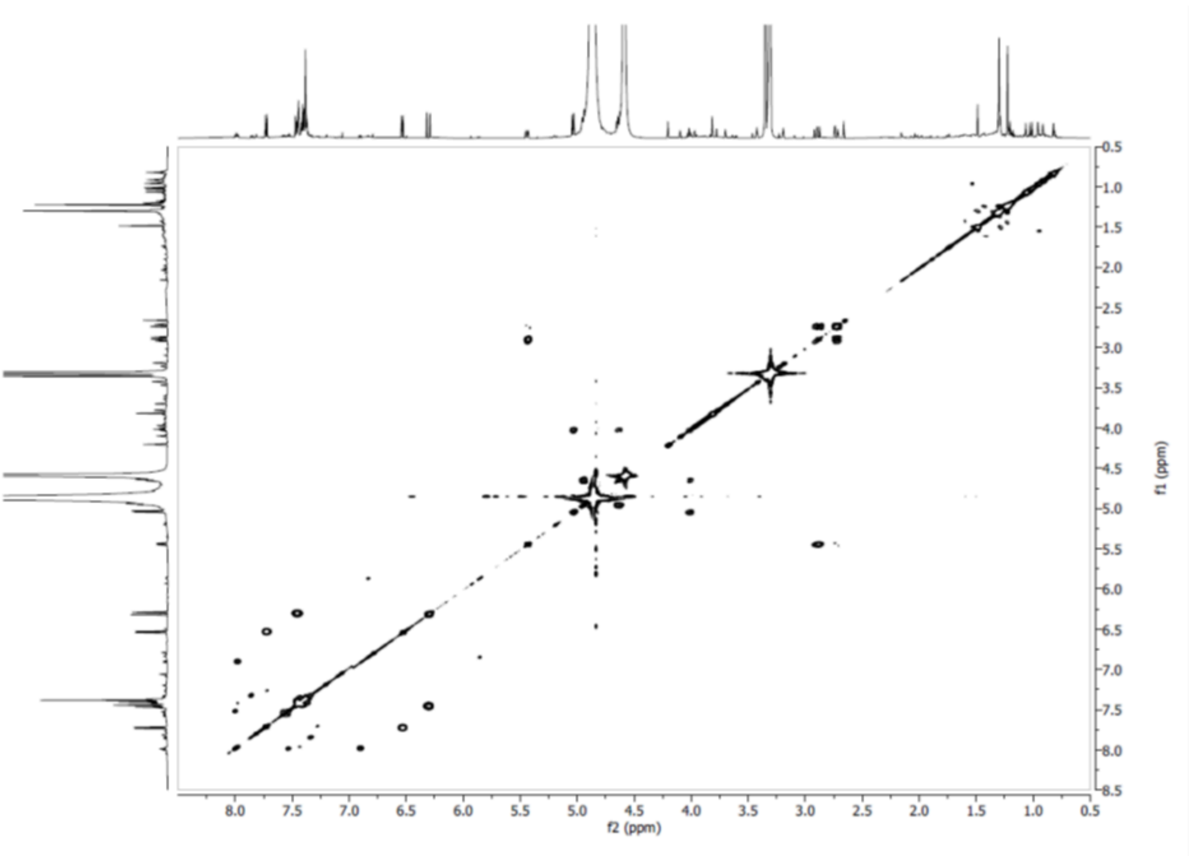

Figure S32: COSY NMR spectrum of compound **14** in CD_3OD

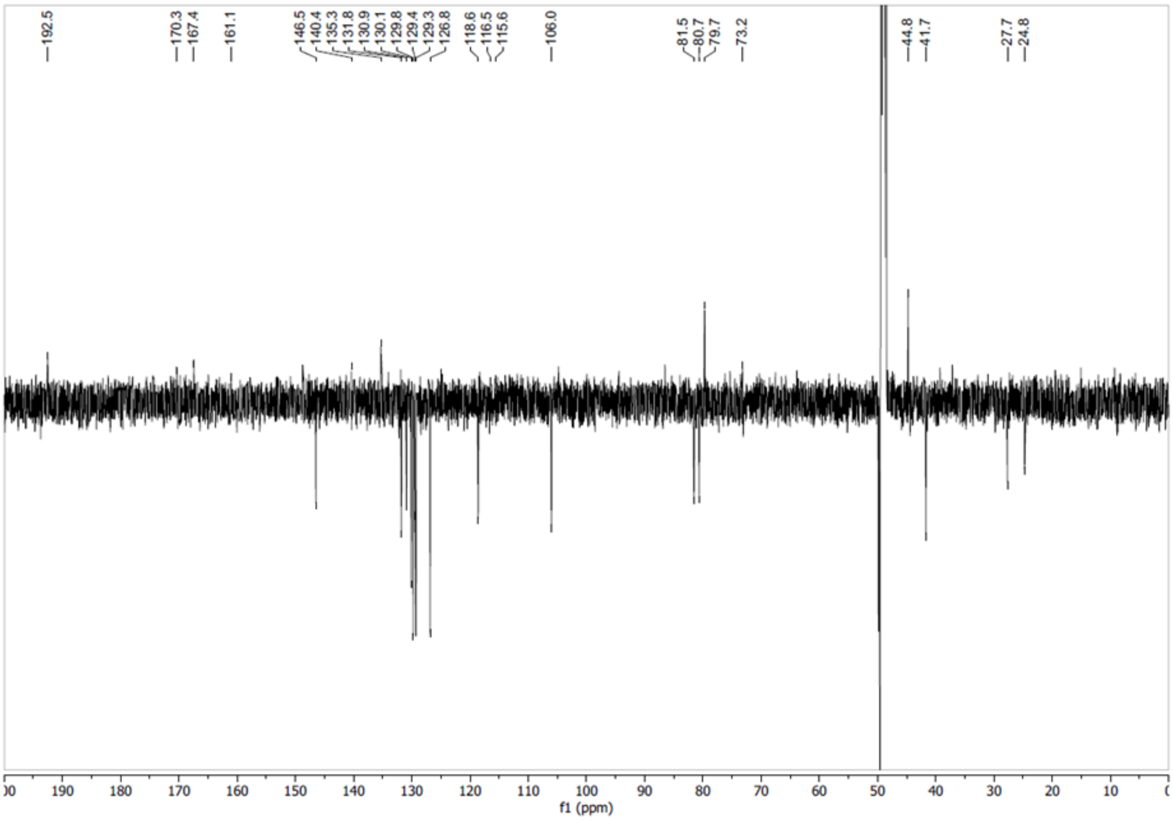

Figure S33: ^{13}C-DEPTQ NMR spectrum of compound **14** in CD_3ODat 151 MHz

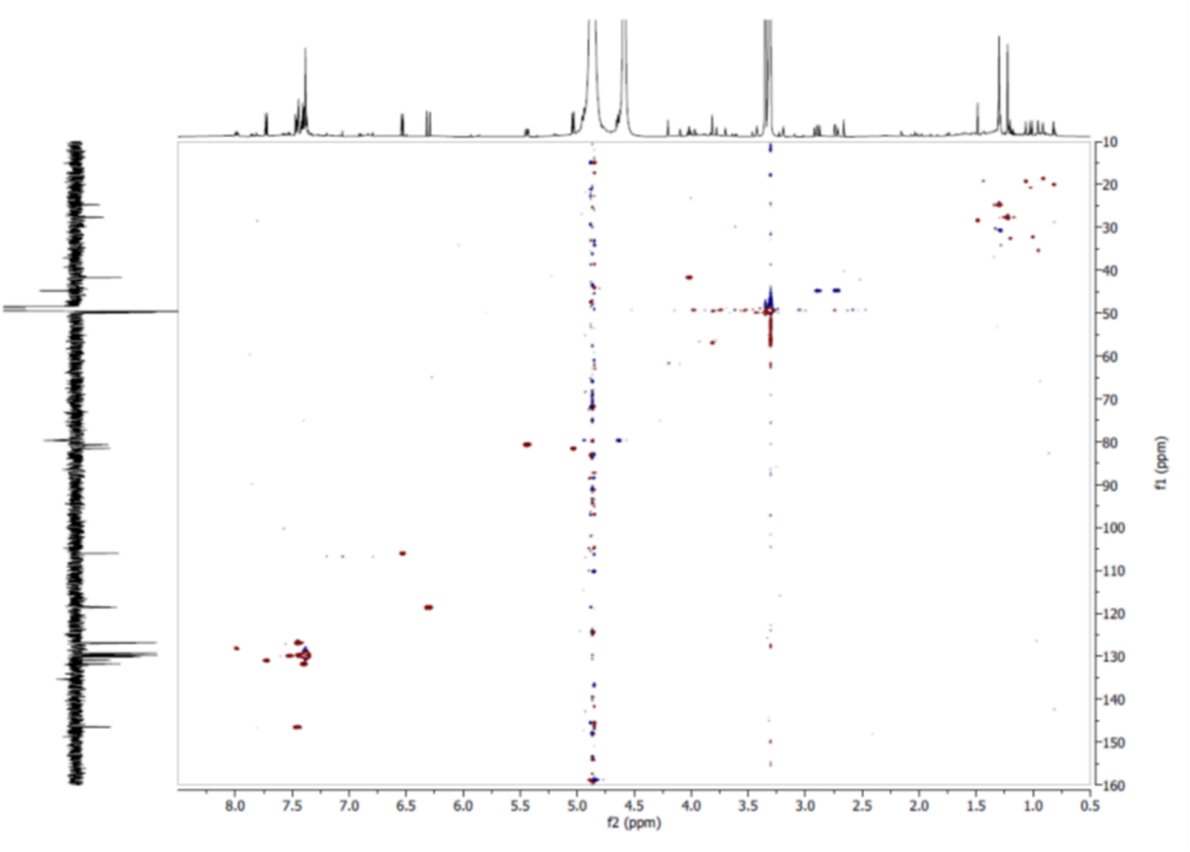

Figure S34: Edited HSQC NMR spectrum of compound **14** in CD_3OD

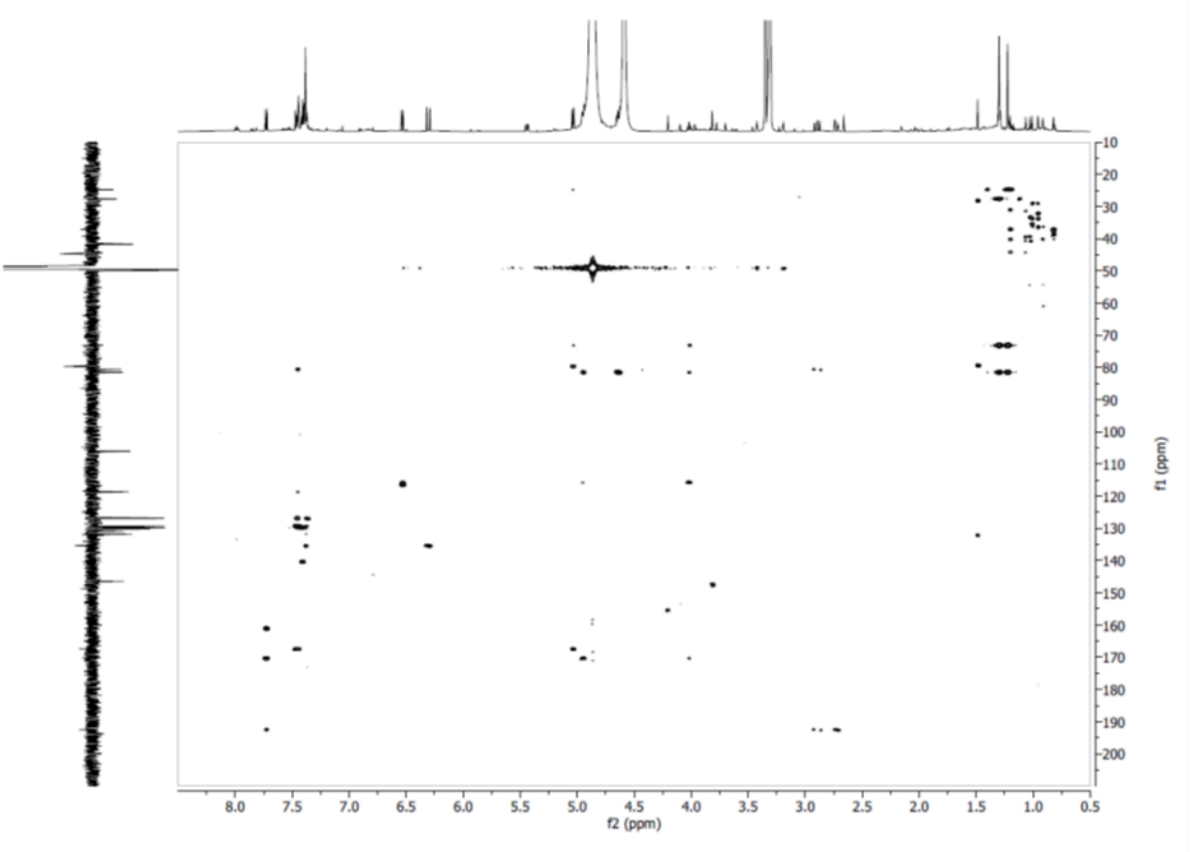

Figure S35: HMBC NMR spectrum of compound **14** in CD_3OD

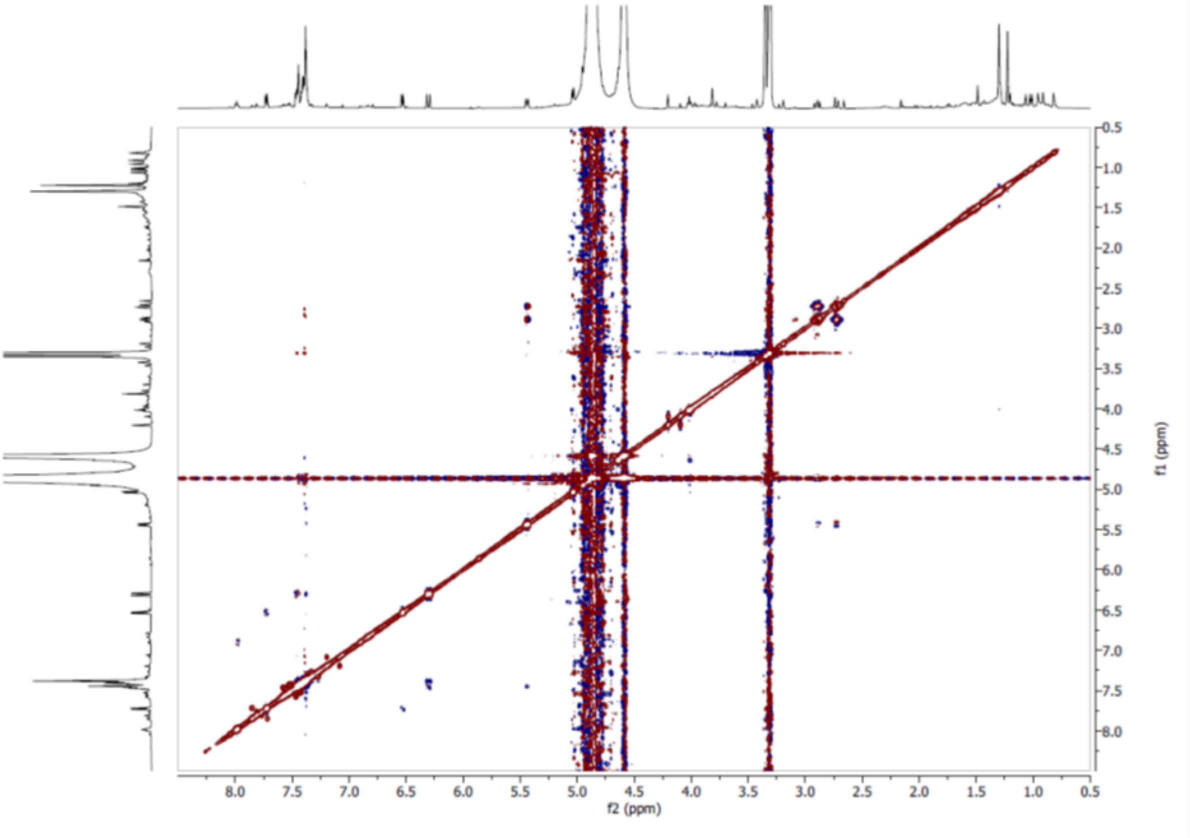

Figure S36: ROESY NMR spectrum of compound **14** in CD_3OD

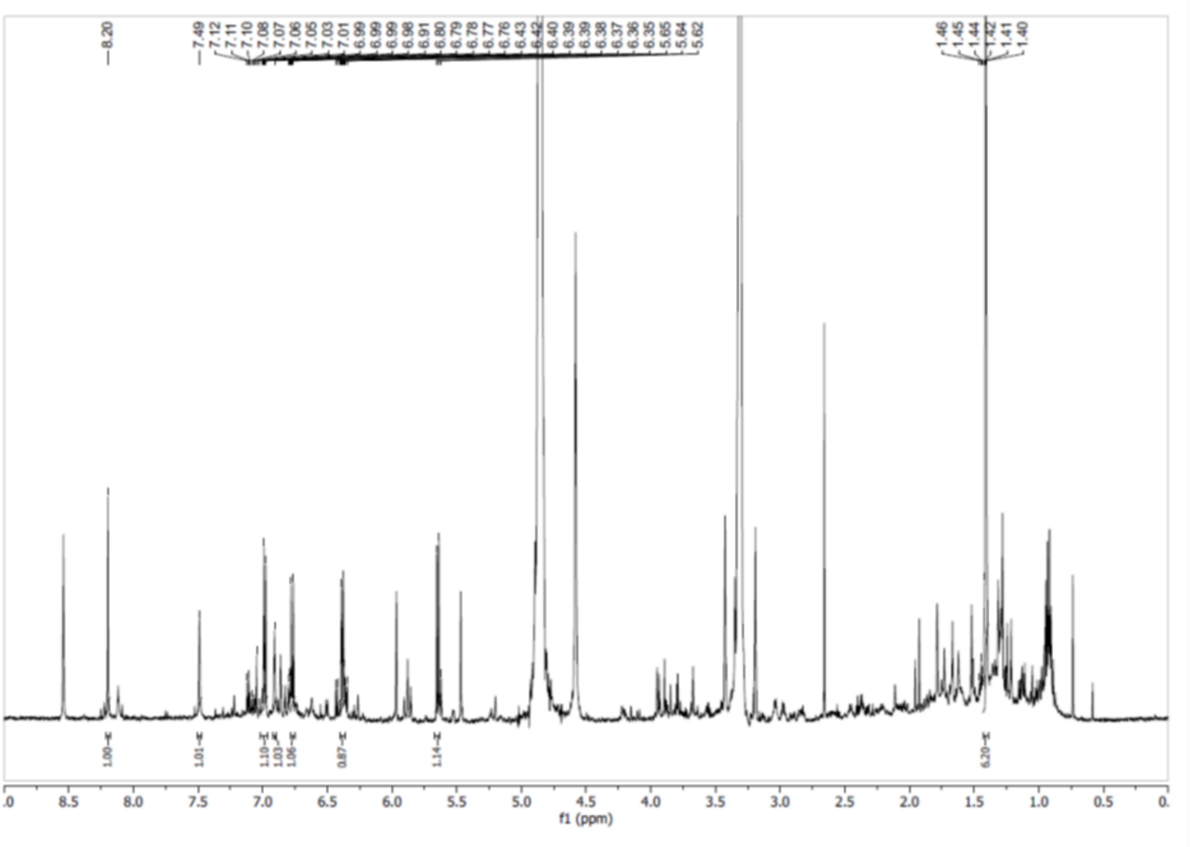

Figure S37: ^{1}H NMR spectrum of compound **21** in CD_3OD at 600 MHz

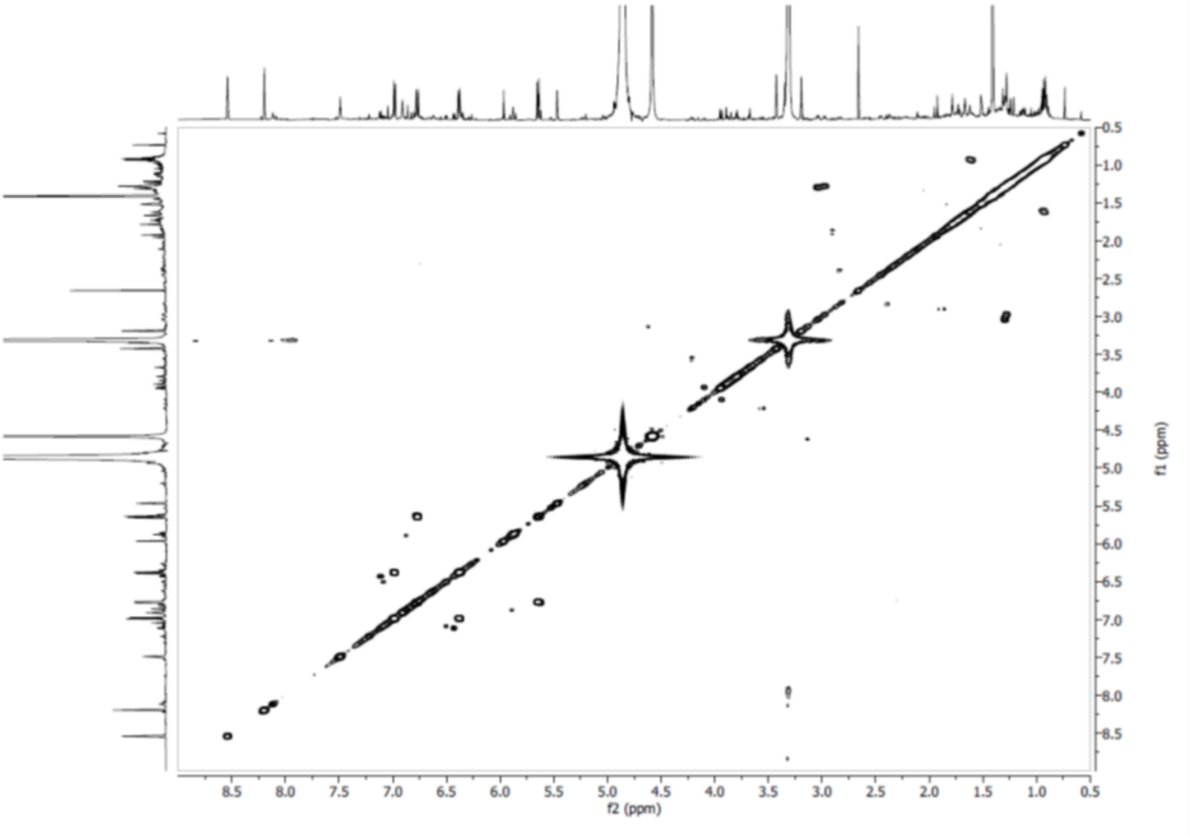

Figure S38: COSY NMR spectrum of compound **21** in CD_3OD

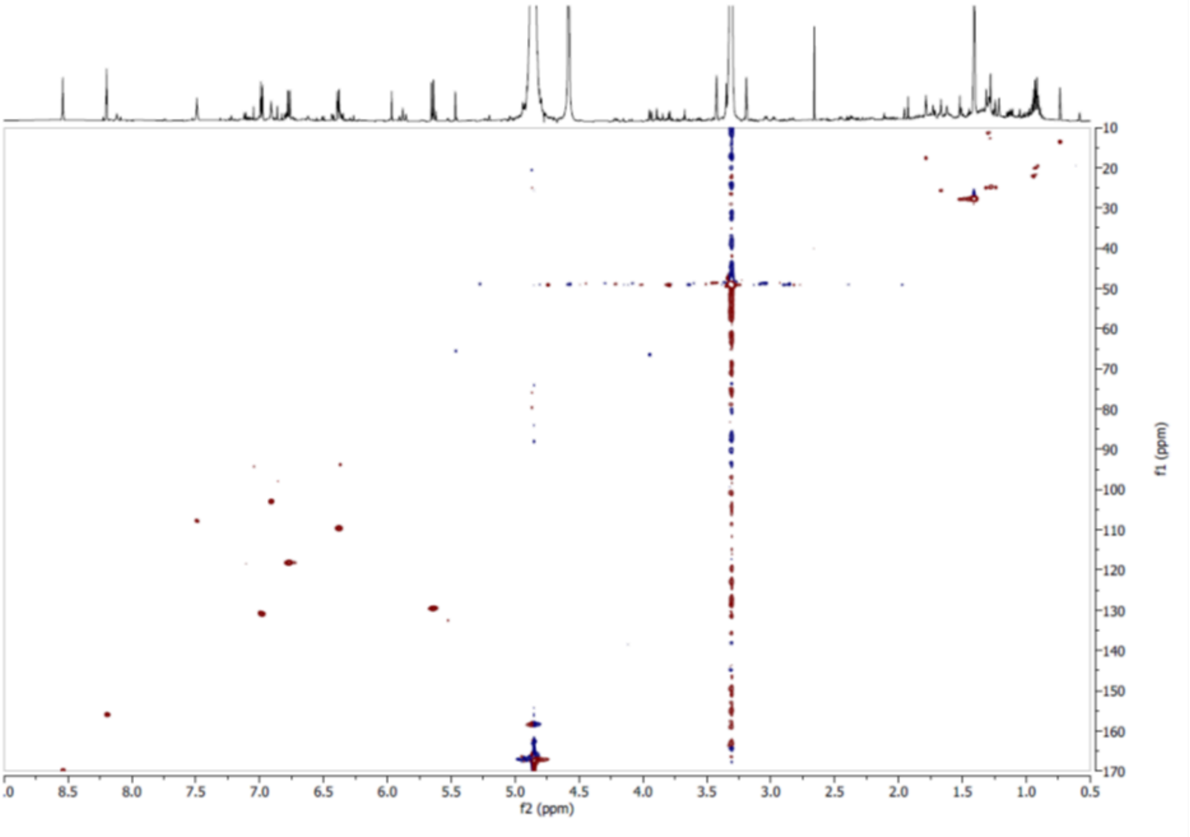

Figure S39: Edited HSQC NMR spectrum of compound **21** in CD_3OD

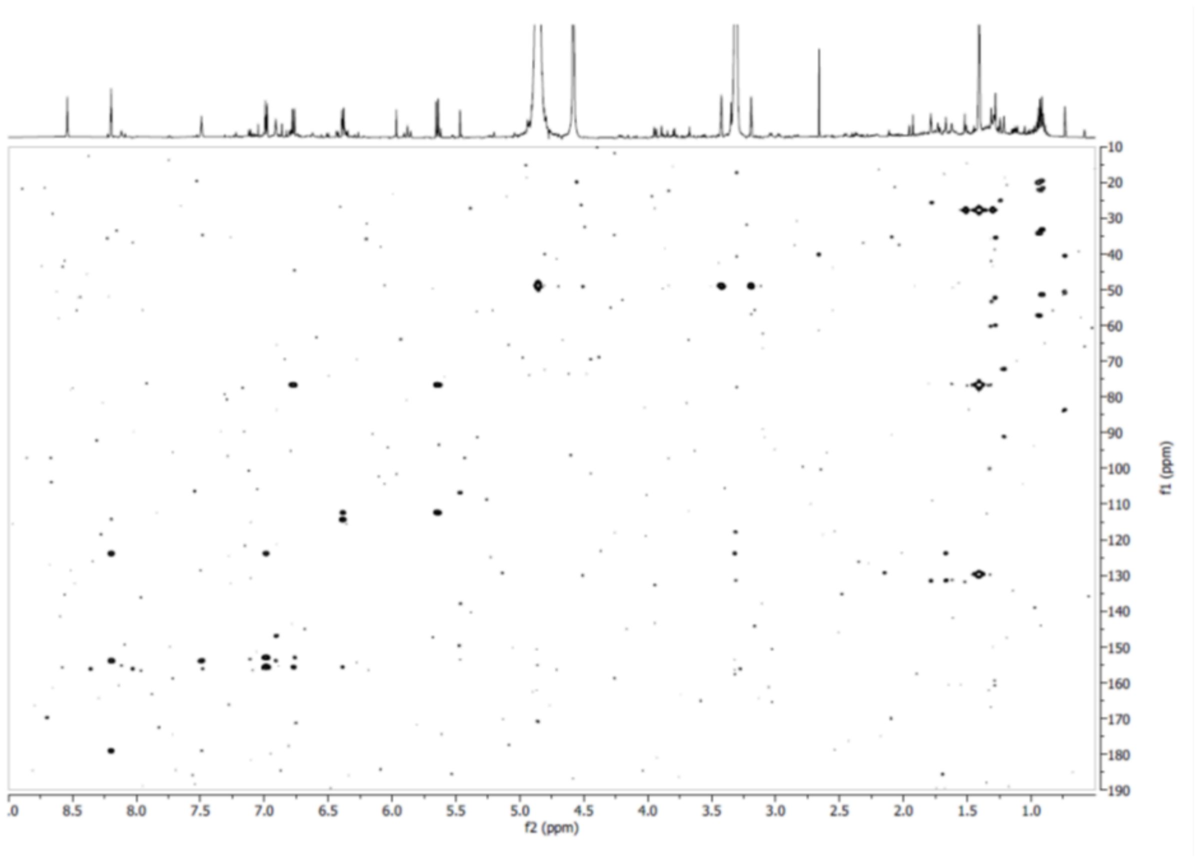

Figure S40: HMBC NMR spectrum of compound **21** in CD_3OD

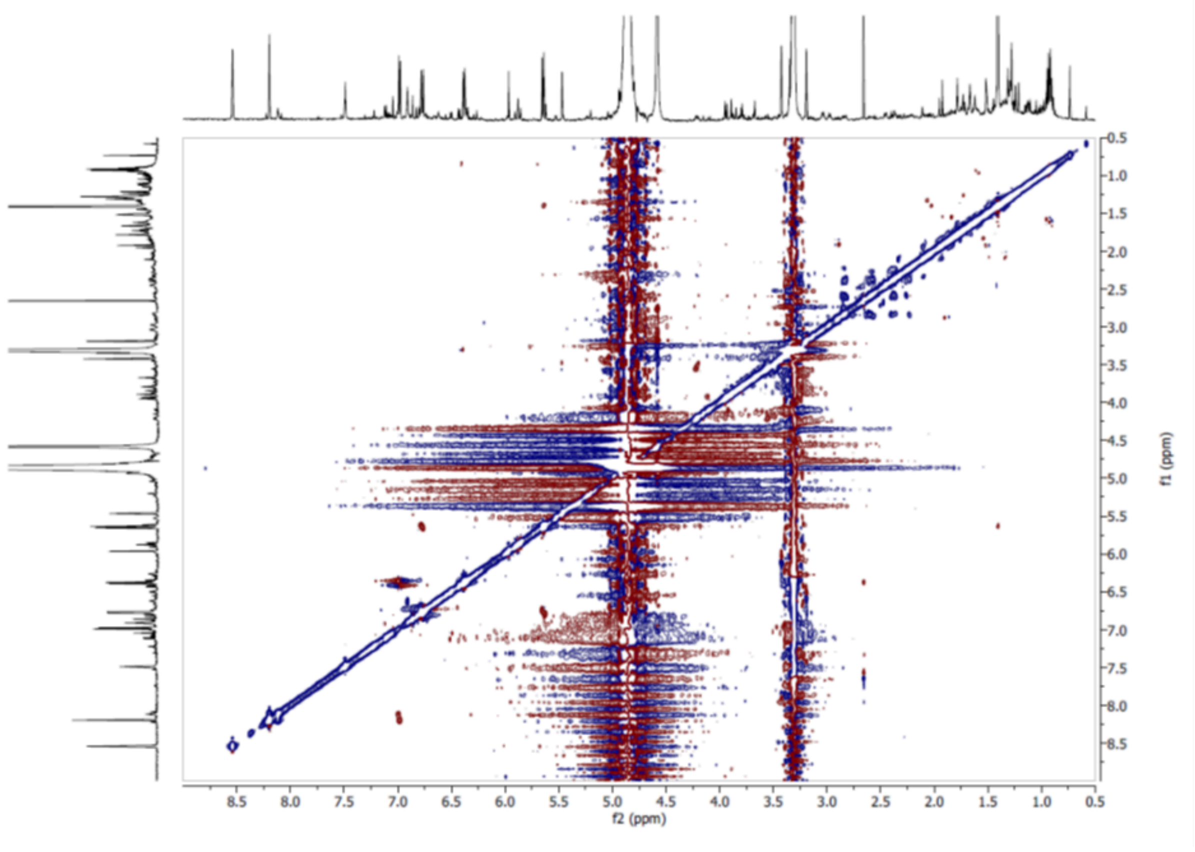

Figure S41: ROESY NMR spectrum of compound **21** in CD_3OD

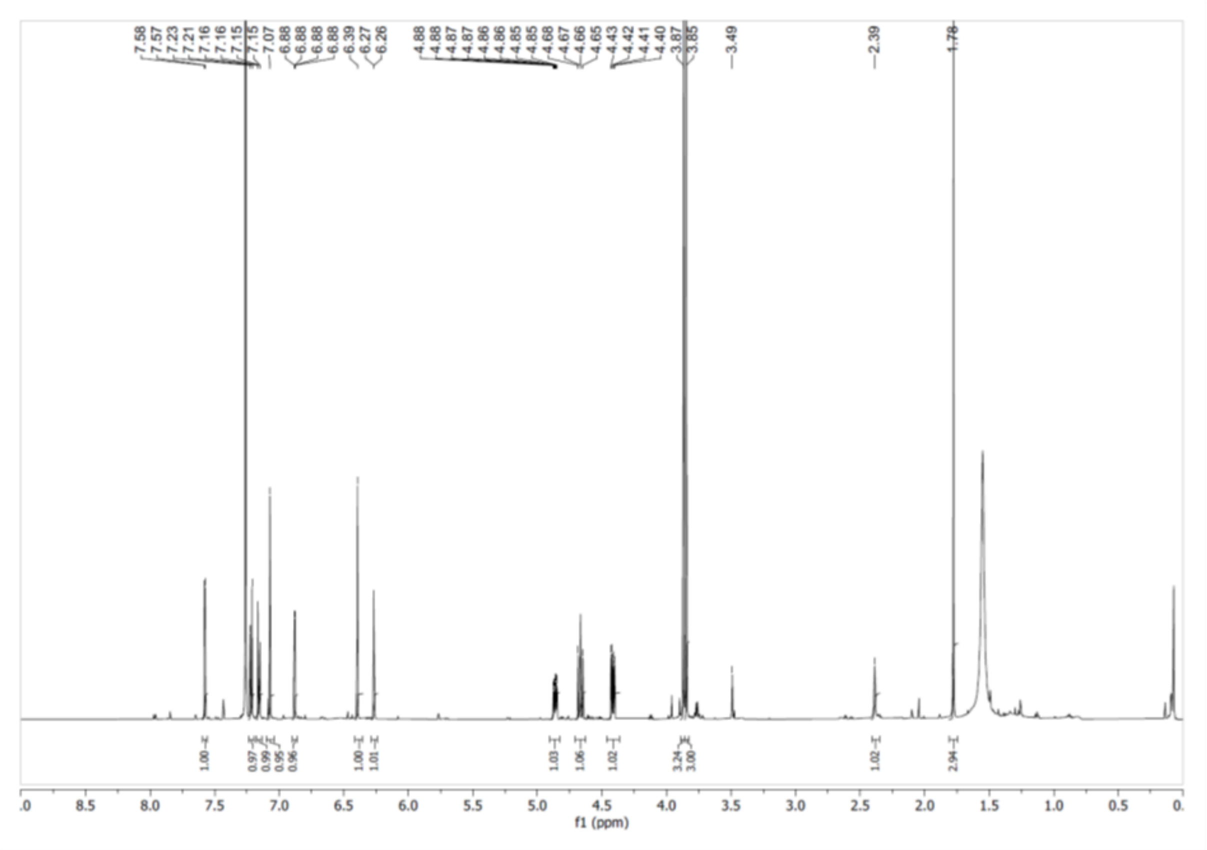

Figure S42: ^{1}H NMR spectrum of compound **33** in $CDCl_3$ at 600 MHz

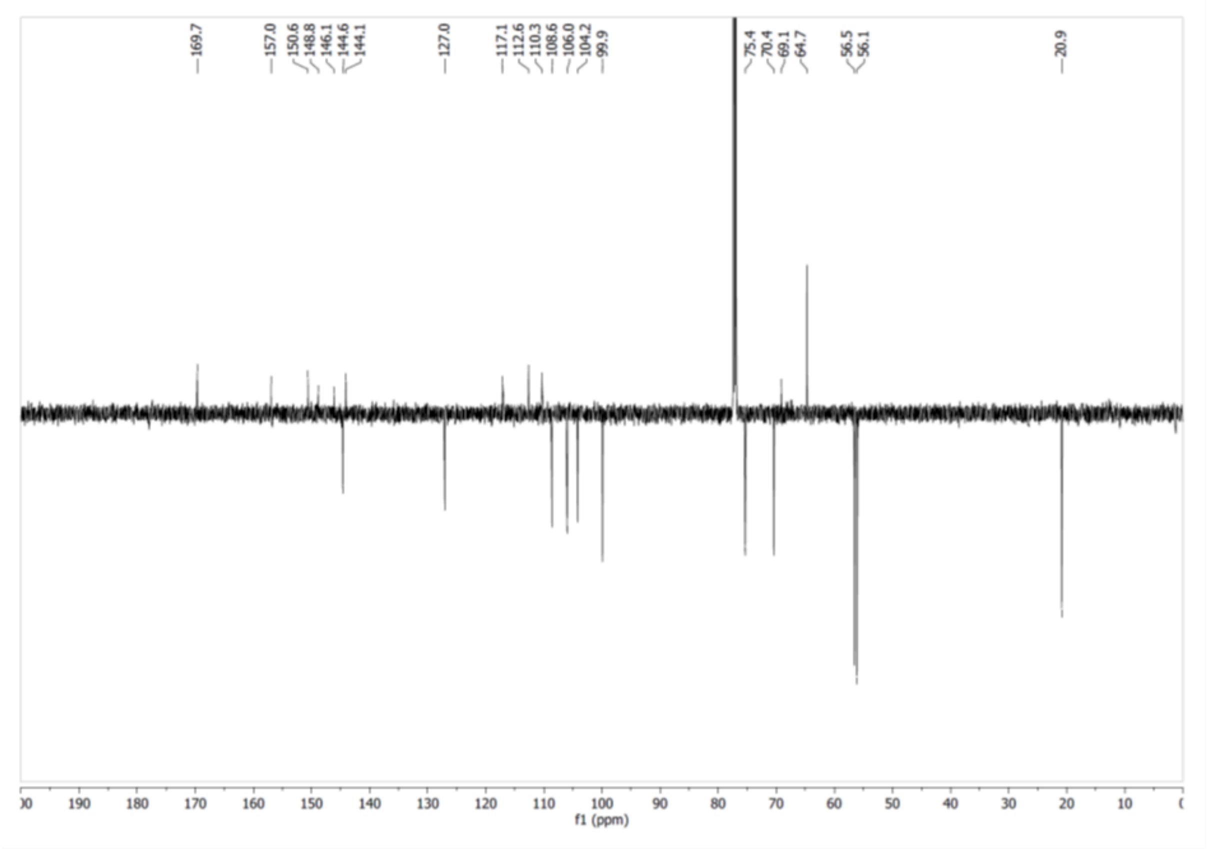

Figure S43: ^{13}C-DEPTQ NMR spectrum of compound **33** in $CDCl_3$ at 151 MHz

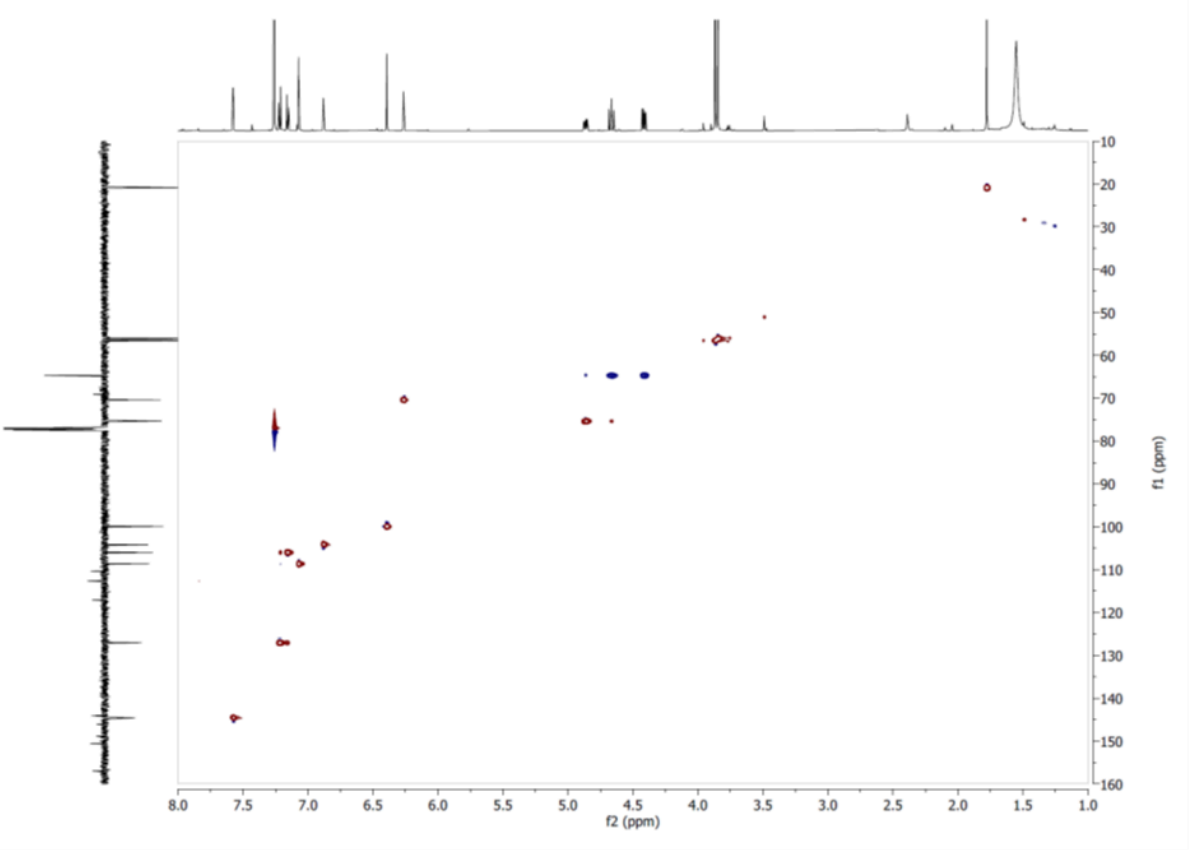

Figure S44: Edited HSQC NMR spectrum of compound **33** in $CDCl_3$

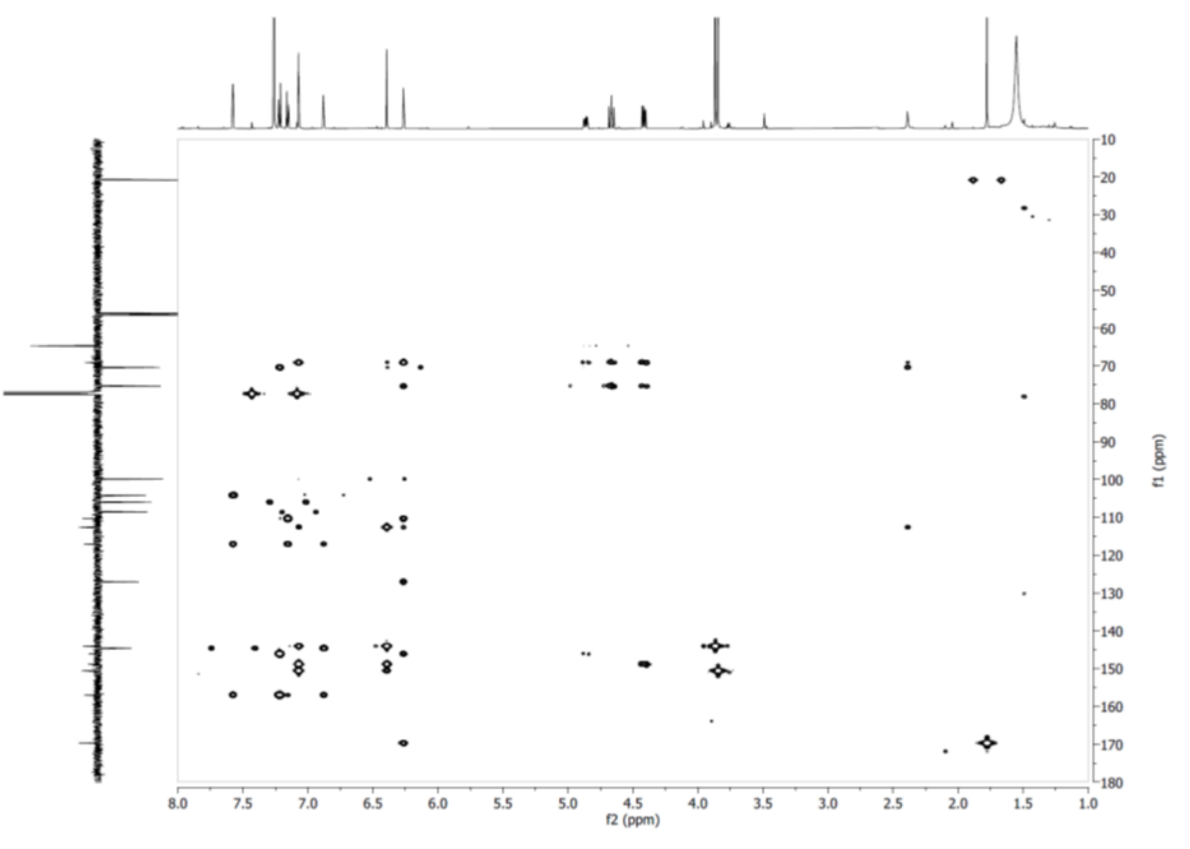

Figure S45: HMBC NMR spectrum of compound **33** in $CDCl_3$

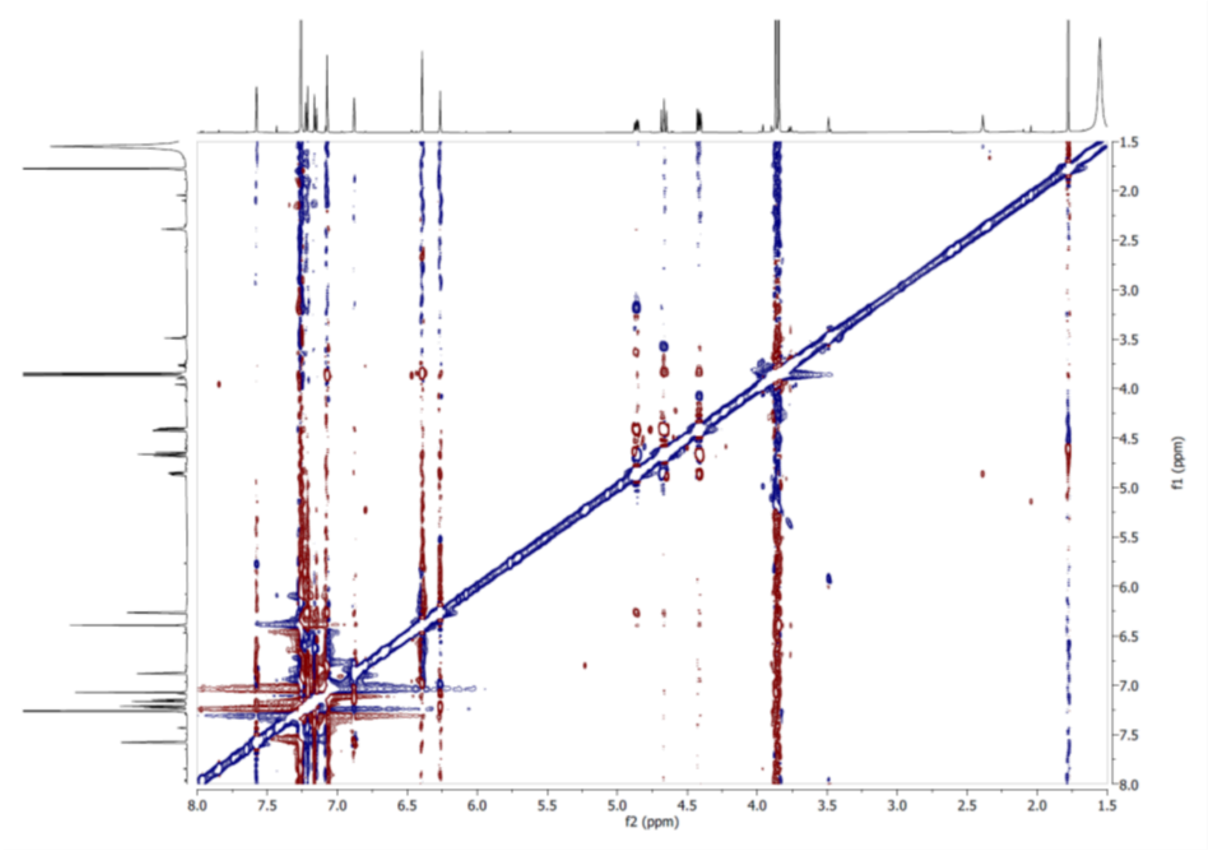

Figure S46: ROESY NMR spectrum of compound **33** in $CDCl_3$

Reference

1. Sakurai Y, Sakurai N, Taniguchi M, Nakanishi Y, Bastow KF, Wang X, et al. Rautandiols A and B, pterocarpans and cytotoxic constituents from *Neorautanenia mitis*. J Nat Prod. 2006;69: 397–399.
